# Multi‐Stimulus Triggered Programmable Transformation of Molecular Motor Based Chiral Supramolecular Polymers in Water

**DOI:** 10.1002/anie.202521360

**Published:** 2026-01-09

**Authors:** Jinghao Wang, Marc C. A. Stuart, Ben L. Feringa

**Affiliations:** ^1^ Stratingh Institute for Chemistry University of Groningen Nijenborgh 3 Groningen 9747 AG The Netherlands; ^2^ Groningen Biomolecular Sciences and Biotechnology Institute University of Groningen Nijenborgh 7 Groningen 9747 AG The Netherlands

**Keywords:** Chemical triggered transformation, Chirality, Light‐responsive materials, Molecular motors, Supramolecular polymers

## Abstract

A notable characteristic of living organisms is their capacity to adapt to environmental changes and transform external signals into distinct responsiveness, facilitating the execution of diverse functions with motility as a key parameter. To better mimic such lifelike behavior, researchers have developed various supramolecular assembled systems with responsive behavior toward a variety of stimuli. However, exploiting motion along length scales and achieving collective control over the responsiveness to multiple stimuli in supramolecular systems is still challenging. Here we present the development of molecular motor based supramolecular polymers that are responsive toward multi‐stimulus and exhibit multi‐state assembly and chirality. Taking advantages of aldehyde functionalized motors, we realized photo‐responsive supramolecular polymers featuring boosted photo‐efficiency, near quantitative photoconversions, programmable behavior and responsiveness to multiple stimuli in a reversible manner in aqueous media. The various stimuli including light and different chemicals could act on the motor building blocks and subsequently trigger the transformation of the supramolecular polymers toward reversible polymerization, direct post‐functionalization and chirality modulation. The interplay between the rotary molecular motion and the supramolecular systems assembly process, taking advantage of different external stimuli to govern the assembly state, provides a basis for multi‐responsive supramolecular materials

## Graphical abstract

We report aldehyde motor–based chiral supramolecular polymers that exhibit light and chemically driven dual responsiveness in water. The supramolecular polymers show transformation and amplification of multi‐state chirality into diverse geometries, offering dynamic control of supramolecular chirality and architectures.

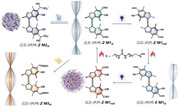

## Introduction

In nature, living systems typically show motility and assembly/disassembly processes, enabling them to achieve complex functions in order to regulate reproduction, growth, development, and evolution among others. One of the fundamental principles is to receive vast amounts of sensory data from the surrounding environment and transduce this signal information into precise, context‐dependent adaptive behavior.^[^ ^1^, ^2^, ^3^, ^4^ ^]^ In order to better understand such processes and mimic lifelike behavior, researchers in recent years have devoted tremendous efforts toward the design and manipulation of sophisticated (supra‐) molecular systems that respond to distinct stimuli in a variety of scenarios using light, chemicals, pH, or redox potentials.^[^ ^5^, ^6^, ^7^, ^8^, ^9^, ^10^, ^11^, ^12^, ^13^ ^]^ Molecular motors have gained much interest due to the controllable motion, unidirectionality and interconvertible inherent chirality, providing opportunities in, e.g., asymmetric catalysis, adaptive actuators, artificial muscles, transporters, responsive materials and nano devices.^[^ ^14^, ^15^, ^16^, ^17^, ^18^, ^19^, ^20^, ^21^, ^22^ ^]^ Despite significant advances toward artificial light‐ and chemical‐driven motor systems, the investigation of motion with a nonreciprocal and cyclic trajectory has largely been limited to the single‐molecule level.^[^ ^23^ ^]^ Exploring molecular motion from the nano‐to‐macroscopic dimensions is increasingly crucial, as it may not only help to amplify intrinsic molecular motor based dynamic processes along length scales but also induce dissipative transformations and cooperativity of motor function akin biomolecular machines.^[^ ^24^, ^25^ ^]^

Supramolecular polymers are a subset of polymers constructed by non‐covalent interactions between small functional monomers, showing broad applications in imaging, drug delivery and release, shape‐memory, recyclable, and bio‐mimetic materials.^[^ ^26^, ^27^, ^28^, ^29^, ^30^ ^]^ The reversibility and dynamicity of the supramolecular polymers empower the controllable expression of monomeric and polymeric functions and offer the path to the developments of responsive supramolecular materials, which employ one of the specific stimuli to switch small molecules and translate the effects into macroscopic changes.^[^ ^31^, ^32^, ^33^, ^34^, ^35^ ^]^ Recently, through hierarchical assembly of molecular motors, we have achieved macroscopic manifestation of the switching behavior and multi‐state chirality control in supramolecular polymers.^[^ ^36^, ^37^ ^]^ However, the traditional design of our molecular motor displayed a relative low switching quantum yield and poor photoconversion in aqueous media, which limited selectivity while prevented further functionalization.^[^ ^38^ ^]^ Meanwhile, chemical triggers have found widespread applications in responsive supramolecular polymers, where the underlying dynamic supramolecular mechanisms may differ from those controlled by light.^[^ ^39^, ^40^, ^41^, ^42^, ^43^, ^44^, ^45^, ^46^, ^47^ ^]^ However, the simultaneous introduction of chemical stimuli in light‐responsive systems remains scarce, which greatly limits the construction of multi‐responsive supramolecular polymers.^[^ ^48^, ^49^, ^50^, ^51^ ^]^ Therefore, we envisioned that integrating the use of molecular rotary motors that can operate through multiple states, as well as applying light and chemical stimuli into a single supramolecular system may allow for the orthogonal operation and facilitate the development of sophisticated artificial motor based assemblies to design future multifunctional systems reminiscent of motor proteins.

**Figure 1:**
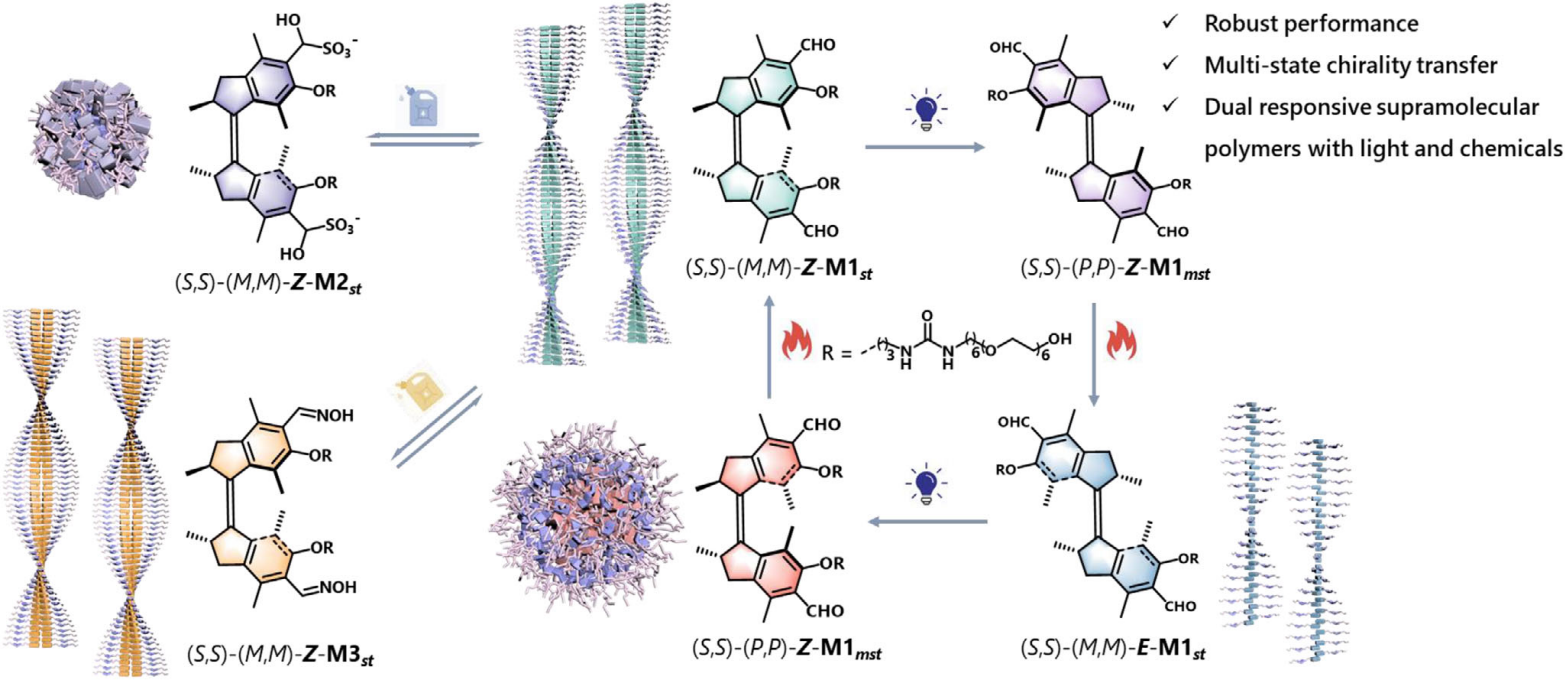
Molecular rotary motor based multi‐state supramolecular systems triggered with light and chemicals, manifesting molecular isomerization, chirality, and morphology control.

Toward this goal, we have constructed aldehyde motor based chiral supramolecular polymers that exhibit multi‐responsiveness toward both light and chemical stimulus (Figure 1). The synthetic aldehyde motor based amphiphiles self‐assemble in aqueous media with the preservation of robust photo‐responsive performance, resulting in the formation of chiral nano‐fiber with the molecular chirality expressed into the supramolecular polymers. Upon irradiation the molecular motors underwent unidirectional rotation via different states with distinct chirality, enabling a dissipative system with controlled transitions between supramolecular assemblies featuring different morphologies. Moreover, by taking advantages of the aldehyde groups, the motors could also react with different nucleophiles, resulting in either reversible disassembly or direct functionalization of the supramolecular polymers. The dynamic behavior provided insight into the orthogonal interplay between external stimuli, the molecular motor activity and the supramolecular assembled systems.

## Results and Discussion

### Molecular Design, Synthesis, and Photo‐Responsive Properties of Motor Monomers

**Figure 2:**
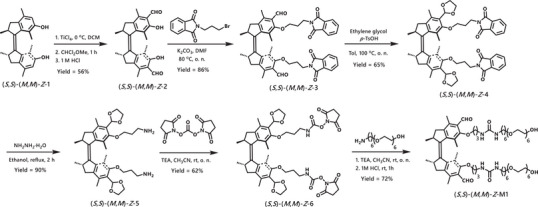
Synthetic route toward the monomer (***S***, ***S***)‐(***M***, ***M***)‐***Z***‐**M1**.

The motor monomers were designed by functionalization of our recently reported highly efficient first‐generation bis‐aldehyde motors with amphiphilic side chains and embedded bis‐urea groups to engage in multiple intermolecular hydrogen bonding (Figure 2).^[^ ^36^, ^38^ ^]^ The detailed synthesis route of (***S***, ***S***)‐(***M***, ***M***)‐***Z***‐**M1** is summarized in the Section S2. The C3 and C6 alkyl‐linkers are positioned aside the urea groups to create a hydrophobic pocket facilitating the formation of hydrogen bonds, which favors the formation of supramolecular polymers in the aqueous media.^[^ ^52^, ^53^ ^]^ Hexa‐ethylene glycol chains are introduced enabling water‐solubility. In the present study the (***S***, ***S***)‐(***M***, ***M***)‐***Z***‐**M1** enantiomer has exclusively been applied for the experiments, therefore the designation of the stable isomer (***S***, ***S***)‐(***M***, ***M***)‐***Z***‐**M1** is shortened as ***Z***‐**M1*_st_***. All the novel structures were fully characterized by ^1^H and ^13^C‐NMR, and high‐resolution ESI‐MS.

**Figure 3:**
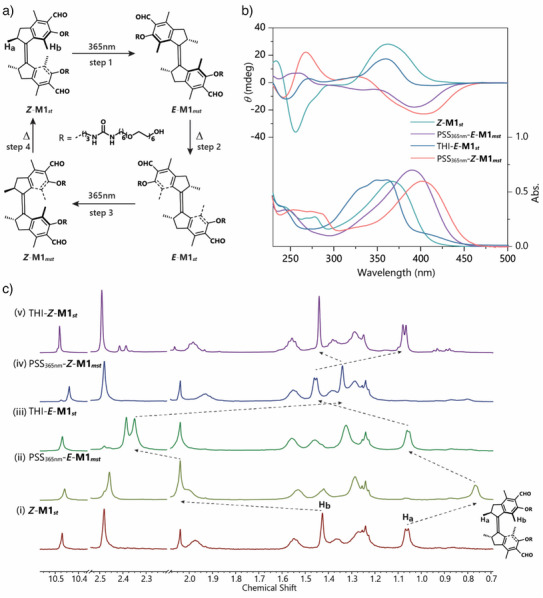
Photo‐responsive properties of aldehyde functionalized motor M1. a) Depicted are the structural changes during the four‐step unidirectional isomerization of a first‐generation aldehyde motors. b) Changes in the UV–vis (right axis) and CD (left axis) absorption spectra of ***Z***‐**M1*_st_*** (30 µM, methanol) upon irradiation with 365 nm light for 2 min to reach the PSS_365nm_‐***E***‐**M1*_mst_*** and subsequently removing the light source and warming at 258 K for 1 h to allow THI‐***E***‐**M1*_st_***, followed by re‐irradiation at 365 nm for 1 min to reached PSS_365nm_‐***Z***‐**M1*_mst_*** and finally removing the light source and warming at 328 K for 5 h to reach THI‐***Z***‐**M1*_st_***. c) Partial ^1^H NMR spectra monitoring the isomerization and showing unidirectional rotation through 4 steps of (i) ***Z***‐**M1*_st_*** (CD_2_Cl_2_, 2 mM), (ii) PSS_365nm_‐***E***‐**M1*_mst_***, (iii) THI‐***E***‐**M1*_st_***, (iv) PSS_365nm_‐***Z***‐**M1*_mst_***, (v) THI‐***Z***‐**M1*_st_***.

To explore the performance of the aldehyde motor based amphiphiles, we have first investigated the unidirectional rotary behavior of ***Z***‐**M1*_st_*** monomer in methanol by UV–vis, CD and ^1^H‐NMR spectroscopy. The fully 360° unidirectional rotation of molecular motors involves the generation of two photostationary states (PSS) (two photochemical steps; step 1 and step 3) and two subsequent thermal helix inversion (THI) steps (step 2 and step 4, Figure 3a). Initially, upon irradiation with a 365 nm LED at −15 °C, the characteristic absorption band of ***Z***‐**M1*_st_*** at 340–400 nm decreased and an absorption band at 370–450 nm appeared with an isosbestic point at 376 nm, which indicated the selective photochemical interconversion of ***Z***‐**M1*_st_*** to the photostationary state named PSS_365nm_‐***E***‐**M1*_mst_*** (Figure S2a). In the CD spectrum, the characteristic positive signal of ***Z***‐**M1*_st_*** at 330–400 nm disappeared with the generation of a negative band at 360–440 nm upon irradiation, which also reflects the formation of PSS_365nm_‐***E***‐**M1*_mst_*** with opposite chirality (Figure 3b). Subsequent keeping the solution in the dark at −15 °C resulted in the fading of the absorption band at 360–440 nm and formation of a new stable absorption band at 365 nm from the UV–vis spectra. Similarly, the transformation was observed in the CD spectra from a negative band at 360–440 nm to a positive signal at 340–390 nm (Figure 3b and Figure S2b), due to the thermal helix inversion from metastable state PSS_365nm_‐***E***‐**M1*_mst_*** to THI‐***E***‐**M1*_st_***.

Following the THI process, irradiation of THI‐***E***‐**M1*_st_*** induced the second photochemical isomerization (step 3), accompanying with the bathochromic maximum absorption band from 365 nm to 400 nm and inverted negative band of CD signals at 350–450 nm, which indicated the generation of PSS_365nm_‐***Z***‐**M1*_mst_*** (Figure 3b and Figure S2c). This ***E_st_*** → ***Z_mst_*** photoisomerization processes were drastically accelerated and completed in 1 min compared with our earlier functional first generation motors, which takes up to 30 min to finish the ***E*** → ***Z*** isomerization process.^[^ ^36^ ^]^ The accelerated photoisomerization process is attributed to the higher quantum yield of the aldehyde‐functionalized motor, reaching 80%, compared to around 37% for non‐aldehyde analogues.^[^ ^38^ ^]^

After warming the solution at 328 K for 5 h, nearly identical UV–vis and CD spectra were obtained as the initial isomer with the absorption at 365 nm and a positive band in CD spectrum and the recovery was attributed to the THI process from PSS_365nm_‐***Z***‐**M1*_mst_*** to ***Z***‐**M1*_st_*** (Figure 2b and Figure S2d). Eyring analysis of the THI processes in methanol in situ monitored by UV–vis spectroscopy, revealing a standard Gibbs free energy of activation of 79.1 and 104 kJ/mol at 293 K (Δ^⋕^ *G'* for the ***E***‐**M1*_mst_*** to ***E***‐**M1*_st_*** and Δ^⋕^ *G“”* for the metastable ***Z***‐**M1*_mst_*** to stable ***Z***‐**M1*_st_***, respectively, Figures S4, Figures 5 and 8, and Table S1); the values are comparable with those of the substituted first‐generation molecular motors.^[^ ^38^, ^54^ ^]^

Further ^1^H‐NMR experiments with in situ irradiation also demonstrated the unidirectional transformations of the motors (reflected by the shifts of the proton signals of Ha and Hb, Figure 3c). Proton signals of Ha (*δ* = 1.05 ppm) shift upfield to 0.76 ppm with irradiation and then shift downfield to 1.04 and 1.44 ppm upon further warming and irradiation processes, while Hb shift from 1.43 ppm to 2.04, 2.34 and 1.30 ppm upon alternatively light irradiation or heating (Figure 3c(i–iv)), in accordance with the conversion sequence (***Z***‐**M1*_st_*** →PSS_365nm_‐***E***‐**M1*_mst_*** →THI‐***E***‐**M1*_st_*** →PSS_365nm_‐***Z***‐**M1*_mst_***). Subsequently, warming the sample resulted in the recovery of the initial ^1^H NMR spectrum of ***Z***‐**M1*_st_*** (Figure 3c(v)). It should be noted that the ratio of the THI‐***Z***‐**M1*_st_*** to THI‐***E***‐**M1*_st_*** after 360° rotation is around 90:10 (from integrations of NMR, respectively), manifesting the high conversion at each step reaching the PSS or THI states, which were dramatically increased compared with our earlier functional 1st generation motors owing to high quantum yields and nearly quantitative photoconversions of the aldehyde motor.^[^ ^36^ ^]^

### Chiral Supramolecular Polymers in Water

**Figure 4:**
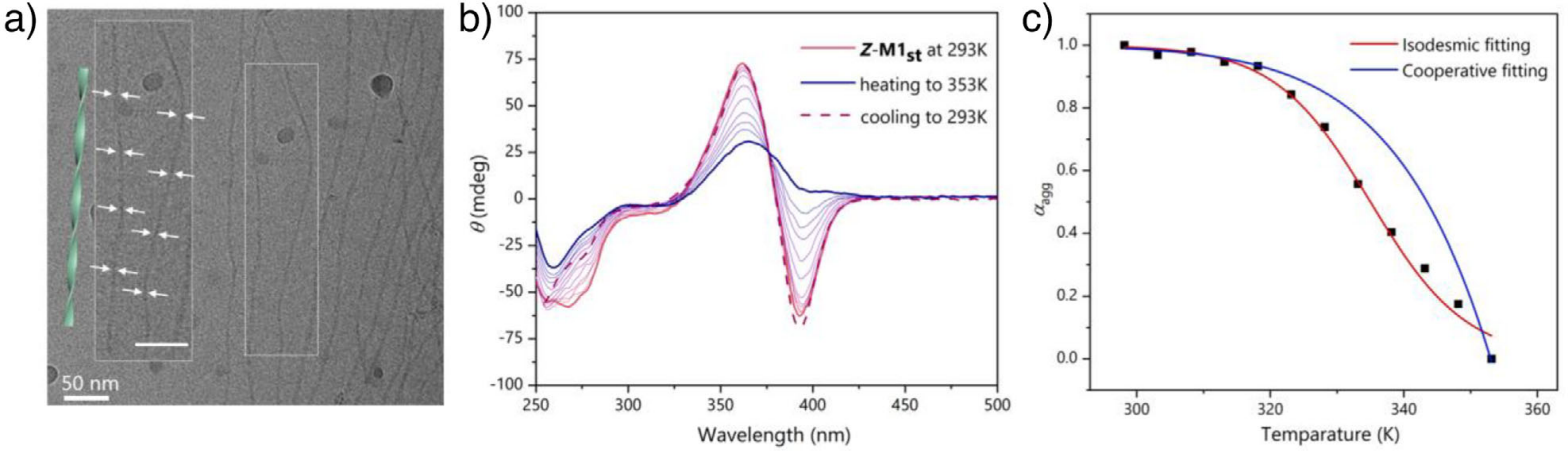
Formation of chiral supramolecular polymers in water. a) Cryo‐TEM image of the assembly structure in water of ***Z***‐**M1*_st_*** (640 µM), with locally magnified area (white box, scale bar: 50 nm) showing helical structure. b) Temperature‐dependent CD spectra of ***Z***‐**M1*_st_*** (30 µM, from 298 K to 353 K) in water. c) Temperature dependent degree of aggregation, *α*_agg_ of ***Z***‐**M1*_st_*** in water based on the CD spectra. The curves show fits calculated according to the isodesmic (red) and cooperative (blue) models.

To investigate whether the motor amphiphiles ***Z***‐**M1*_st_*** show self‐assembly in water, an aqueous solution of ***Z***‐**M1*_st_*** at a concentration of 5 mg/mL was prepared and the aggregates were subsequently characterized by cryogenic transmission electron microscopy (cryo‐TEM), and uniform fibers were found with a diameter of 7.0 ± 0.8 nm and micrometers in length (Figure S12). Next, by diluting the solution of ***Z***‐**M1*_st_*** to 1 mg/mL, a clear regular variation in the width along the axis of the individual fibers was observed, indicating the helical structure of the supramolecular polymer with a 1/2 pitch around 70 nm (Figure 4a and Figure S13). For further investigation of the chirality transfer and polymerization mechanism, we have conducted temperature‐dependent CD spectroscopy measurement by heating the aqueous solution of ***Z***‐**M1*_st_*** (30 µM in water) from 298 K to 353 K (Figure 4b). Different from the CD spectra obtained in methanol, a characteristic negative Cotton effect was observed at relatively lower temperature, indicating the presence of chiral supramolecular polymers.^[^ ^55^ ^]^ Upon temperature increase, the negative Cotton effect gradually diminished with the appearance of a positive band at 330–400 nm in accordance with the CD spectra obtained in MeOH, implying a disorder of the supramolecular polymer and the disappearance of the supramolecular chirality. By monitoring the ellipticity at 400 nm, correlating the values to the formation of helical aggregates and analyzing the data as the temperature‐dependent degree of aggregation, *α*_agg_, a good fitting was observed with the isodesmic model proposed by Meijer et.al. (Figure 4c and Figure S11).^[^ ^56^, ^57^, ^58^ ^]^ After cooling and stabilizing the solution at 298 K for 1d, the positive Cotton effect was recovered, indicating the reformation of the chiral supramolecular polymer. The Cryo‐TEM analysis together with the reversible CD data confirmed transfer and amplification of the chirality from the amphiphilic molecular motor ***Z***‐**M1*_st_*** to the supramolecular polymer in aqueous media.

### Light Driven Dynamics of Molecular Motor Based Supramolecular Polymers in Water

**Figure 5:**
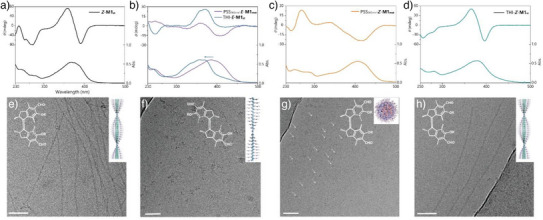
Spectral and morphologies changes of light‐driven rotation of supramolecular polymers. UV–vis and CD absorption spectra of a) ***Z***‐**M1*_st_*** (320 µM, 1 mm cuvette), b) after irradiation with 365 nm light for 2 min at 278 K (PSS_365nm_‐***E***‐**M1*_mst_***) and keeping the sample in the dark for 2 h to reach THI‐***E***‐**M1*_st_***. c) after subsequent irradiation with 365 nm light for 1 min to obtain PSS_365nm_‐***Z***‐**M1*_mst_*** in water, and d) warming in the dark at 343 K for 5 h in water and subsequently cooling to recover THI‐***Z***‐**M1*_st_***. (e–h) Cryo‐TEM images and corresponding structures of the identical samples (a–d), respectively (micelles were pointed out with arrows for clearance, and not all the micelles were indicated). The scale bar in panels e–h) is 50 nm.

It is well known in the field of supramolecular polymers that amphiphiles would arrange in aqueous media with the hydrophilic parts oriented toward the water phase and the hydrophobic heads aggregated, allowing for the formation of self‐assembled polymers.^[^ ^56^, ^59^, ^60^ ^]^ With the introduction of the molecular motors into supramolecular polymers, it is important to understand how the motor moieties rotating upon irradiation operate in the confined assembly state, and how the chirality changes in the multistate system, arising from the conformational changes, influences the expression of supramolecular polymers. Therefore, combined UV–vis, CD spectroscopy and Cryo‐TEM analysis have been applied to investigate the nature of the supramolecular polymers in the aqueous media during the full trajectory of the rotation process of the motor units. Initially, ***Z***‐**M1*_st_*** formed helical fibers and showed a negative Cotton effect at 340–420 nm in the CD spectra and nearly identical UV–vis spectra as observed in methanol (Figure 5a,e). Upon irradiation with 365 nm light for 2 min, the characteristic absorption band of ***Z***‐**M1*_st_*** at 365 nm decreased with the increase of an absorption band at 390 nm. Additionally, the negative Cotton effect attributed to the supramolecular polymer structures disappeared while a negative signal appeared at 380–450 nm in the CD spectra, in accordance with the selective photochemical interconversion of ***Z***‐**M1*_st_*** to PSS_365nm_‐***E***‐**M1*_mst_*** (Figure 5b and Figure S3a). Due to the short half‐life of PSS_365nm_‐***E***‐**M1*_mst_*** in water (3.5 min, Table S1), the real morphologies of PSS_365nm_‐***E***‐**M1*_mst_*** were difficult to establish by Cryo‐TEM. Subsequently keeping the sample in the dark for 2 h resulted in the THI process and the formation of THI‐***E***‐**M1*_st_***, evident from the inverted CD signal from a negative band at 380–450 nm to a positive band at 350–400 nm as well as the blue‐shift of the maximum absorption band to 365 nm (Figure 5b and Figure S3b). After irradiation and subsequently warming to reach THI‐***E***‐**M1*_st_***, the fibre formed by ***Z***‐**M1*_st_*** disappeared and short worm‐like micelles were observed in the cryo‐TEM images featuring about 2 nm width and around 50 nm length (Figure 5f and Figure S14). Next, upon irradiation at 365 nm for 1 min, the absorption band and positive CD signal at 370–410 nm of the THI‐***E***‐**M1*_st_*** diminished with the generation of a bathochromic absorption band and negative CD signal at 390–450 nm, reflecting the photoirradiation process from THI‐***E***‐**M1*_st_*** to PSS_365nm_‐***Z***‐**M1*_mst_***, which contributed to the transformation of short worm‐like micelles into sphere micelles with an average diameter of 5 nm in the aggregated states (Figure 5c,g and Figures S3c and S15).

Interestingly, we could directly observe the recovery of the supramolecular polymers by warming the aqueous sample of PSS_365nm_‐***Z***‐**M1*_mst_*** at 343 K for 5 h and subsequent cooling processes. In our previous studies the recovery of ***Z***‐**M1*_st_*** was difficult to achieve due of the low photoconversion yield of motors in water. As testimony to the remarkable high photoconversion efficiency of the aldehyde motors and the full reversibility achieved in controlling self‐assembly, the samples obtained after the THI processes (THI‐***Z***‐**M1*_st_***) displayed almost similar morphologies and nearly identical UV–vis and CD spectra (Figure 5d,h and Figures S3d,S16,S17) as those of the initial system. Furthermore, ^1^H NMR analysis of the supramolecular polymers at different states revealed a nearly full conversion from ***Z***‐**M1*_st_*** to THI‐***E***‐**M1*_st_*** and a ratio of THI‐***Z***‐**M1*_st_*** and THI‐***E***‐**M1*_st_*** as high as 87:13 after 360° rotation, which is similar to the values obtained in methanol (Figures S9 and S10). The quantum yields for two photo‐isomerization steps was revealed to be 17.3% and 47.6% in water, suggesting the relatively high efficiency of photoisomerization compared with the non‐aldehyde analogues (4.9% for ***Z_st_*** → ***E_mst_***, 20.7% for ***E_st_*** → ***Z_mst_***, respectively) in the aggregated state, indicating that the aldehyde group enhances the efficiency of the photochemical steps. (Figures S18–24 and Table S2). By following the Eyring analysis of the THI in water, both THI processes in the aqueous media revealed higher Gibbs free energy of activation than monomeric state (Δ^⋕^ *G'* of 85.7 kJ mol^−1^ from the ***E***‐**M1*_mst_*** to ***E***‐**M1*_st_*** and Δ^⋕^ *G“”* of 109.6 kJ mol^−1^ from the metastable ***Z***‐**M1*_mst_*** to stable ***Z***‐**M1*_st_***, respectively, Figures S6 and S7), which could be attributed to the generation of aggregations (Figure S8 and Table S1).

### Chemical Triggered Transformation of Molecular Motor Based Supramolecular Polymers in Water

**Figure 6:**
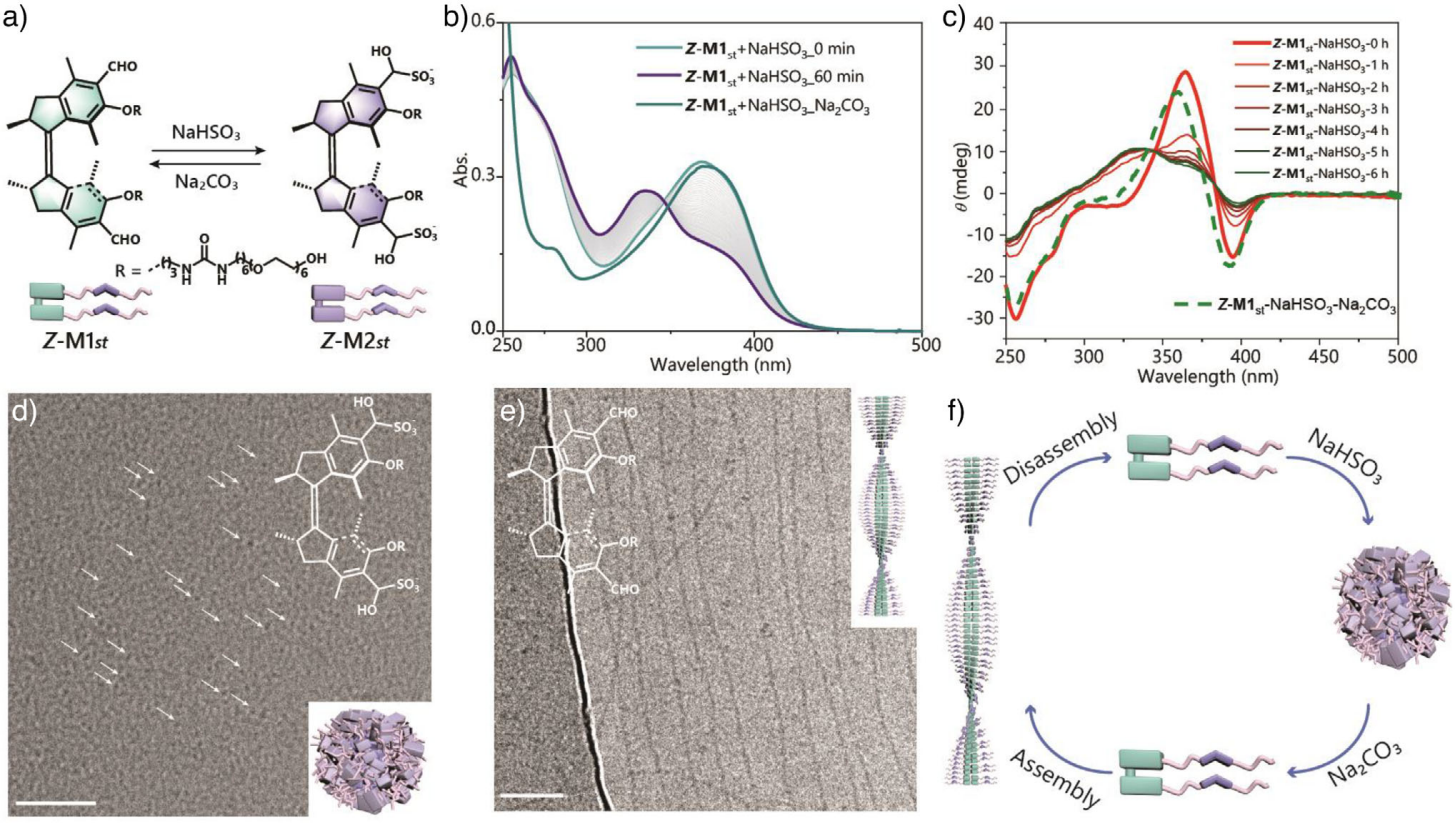
Chemical triggered reversible disassembly of supramolecular polymers. a) Reversible reaction of ***Z***‐**M1*_st_*** with NaHSO_3_ and Na_2_CO_3_ in water. b) UV–vis absorption spectra of ***Z***‐**M1*_st_*** (20 µM in water) upon addition of NaHSO_3_ during 60 min and recovery with addition of Na_2_CO_3_. c) CD spectra of ***Z***‐**M1*_st_*** (20 µM) upon the addition of NaHSO_3_ during 6 h and recovery upon addition of Na_2_CO_3_. Cryo‐TEM images of d) ***Z***‐**M2*_st_*** (1.2 mM in water, after addition) and e) ***Z***‐**M1*_st_*** (recovery from ***Z***‐**M2*_st_*** by addition of Na_2_CO_3_). The scale bar in panels is 50 nm. f) Proposed mechanisms of the dissipative disassembly and re‐assembly of supramolecular‐polymers reaction with NaHSO_3_ and Na_2_CO_3_.

Apart from the robust light‐responsive performance, the aldehyde groups, owing to their ability to undergo reversible nucleophilic addition reactions, might also bring chemical triggered stimuli‐responsiveness, a feature which has recently been incorporated into various responsive supramolecular systems.^[^ ^47^, ^61^, ^62^, ^63^, ^64^, ^65^ ^]^ Inspired by these reports and aiming for integrated light and chemical responsive systems, we have exposed the aldehyde motor based supramolecular polymers in the aqueous environment with different nucleophiles to explore additional dynamic behavior. Sodium bisulfite was initially employed due to its ability to reversibly convert aldehyde groups into α‐hydroxy sulfonate adducts, with the original aldehyde functionality recoverable upon base treatment.^[^ ^66^, ^67^ ^]^ In order to demonstrate the feasibility and identify the products of the bisulfite adducts (Figure 6a), we selected the unfunctionalized aldehyde motor as a preliminary model and confirmed the reversibility of its addition reaction with sodium bisulfite using ^1^H‐NMR spectroscopy (Figures S25–S27).

To further explore the reaction between the supramolecular polymers and bisulfate, UV–vis absorption, CD, and cryo‐TEM measurements were performed at different states. Upon addition of bisulfite to the aqueous ***Z***‐**M1*_st_*** solution, the characteristic absorption of the aldehyde motor unit at 365 nm gradually decreased with the generation of a new band at 310–350 nm, suggesting the conversion of the aldehyde motor ***Z***‐**M1*_st_*** to the ***Z***‐**M2*_st_*** with α‐hydroxy sulfonate substituents (Figure 6b and Figure S28). Consistent with the conversion observed in the UV–vis spectra, the negative Cotton band at 330–400 nm attributed to the supramolecular polymers also diminished with the consistent formation of the positive band at 310–350 nm, suggesting the stepwise disassembly of the supramolecular polymers upon formation of bisulfite adduct (Figure 6c). Consequently, the regular chiral fiber disappeared with the formation of small sphere micelles (∼ 3 nm) as revealed by the Cryo‐TEM analysis (Figure 6d and Figure S39). To understand the mechanism for the chemically triggered process, kinetics analysis of the supramolecular polymers with different equivalents of the NaHSO_3_ solutions as well as in the buffer with pH values were conducted by titration experiments followed by UV–vis and CD spectroscopy (Figures S29–S31,S34). The maximum absorption intensity at 370 and 330 nm revealed that both the extent of transformation and the reaction rate increased with higher concentrations of NaHSO_3_ and low pH values, which could be attributed to the nature of the nucleophilic reaction (Figures S30 and S31d). In the CD spectra, the ellipticity change at 365 nm and 330 nm reflects the appearance of the supramolecular polymers and the generated α‐hydroxy sulfonate. The spontaneous change indicated that the disassembly processes of the supramolecular polymer are accompanying the addition reaction, which reflects the dynamic nature of the triggered system (Figure 6f and Figure S35). Furthermore, the photo‐responsiveness performance of the ***Z***‐**M2*_st_*** with α‐hydroxy sulfonate substituents were investigated, however, the high photo efficiency was lost after sulfonation reaction, contributing to the incomplete rotatory cycle of the motor functions (Figures S37 and S38).

Moreover, the recovery of the aldehyde molecules could be achieved by addition of aq. Na_2_CO_3_ solutions to the α‐hydroxy sulfonate, evident from the re‐generation of UV–vis absorption bands with 4 bisulfite/base cycles as well as the re‐formation of regular long fibers observed by Cryo‐TEM analysis (Figure 6e and Figures S32,S33, S36,S40). Distinct from the gradual transformation process triggered by NaHSO_3_, the recovery of the aldehyde upon base treatment (excess amount Na_2_CO_3_) displayed more rapid processes in the UV–vis and CD spectra (Figure 6b,c). The immediate re‐assembly process could be attributed to the instant transformation of the hydrophilic sulfonate to the recovered aldehyde. The recovered CD spectrum after the bisulfite/base cycles indicated the slightly incomplete recovery of the original fibers, thus accounting for the minor spectral deviation from the initial state (Figure 6c).

**Figure 7:**
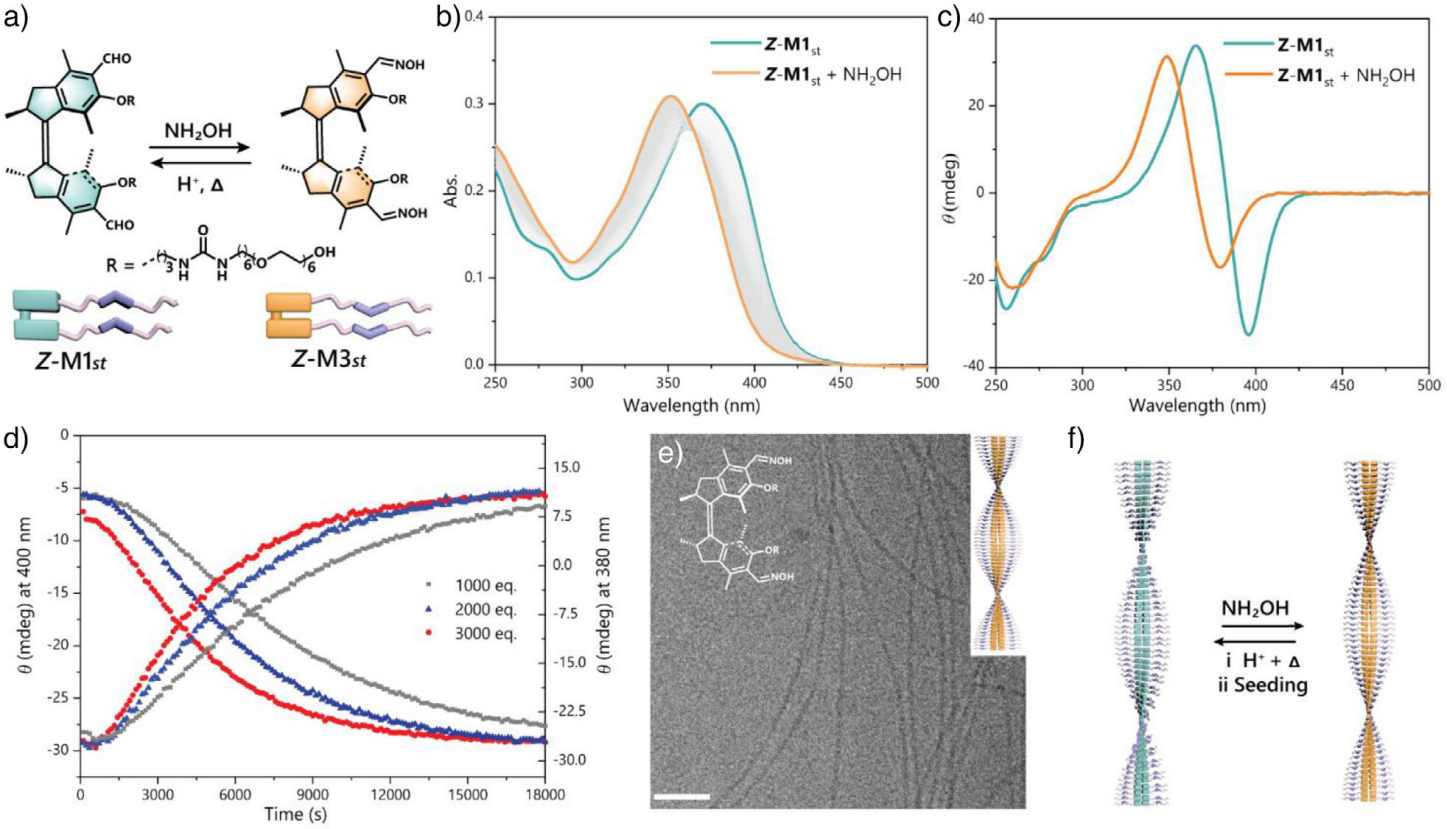
Functionalization of supramolecular polymers in water. a) Reaction of the ***Z***‐**M1*_st_*** with NH_2_OH. b) Change in UV–vis absorption spectra of ***Z***‐**M1*_st_*** (20 µM) upon addition of NH_2_OH during 5 h period. c) CD spectra of ***Z***‐**M1*_st_*** (20 µM) and after addition of NH_2_OH. d) Ellipticity at 380 nm (right axis) and 400 nm (left axis) monitored upon the addition of different equivalents of NH_2_OH. e) Cryo‐TEM images of ***Z***‐**M3*_st_*** (1.2 mM) obtained by addition of NH_2_OH in water, the scale bar in panel is 50 nm. f) Reversible transformation of supramolecular polymers upon reaction with NH_2_OH and hydrolysis process.

In addition to the reversible bisulfite addition, we envisioned the formation of imine bonds as another promising strategy toward the modulation of the motors functionalized with aldehyde groups.^[^ ^68^, ^69^, ^70^ ^]^ In order to demonstrate the tailored responsiveness, hydroxylamine was selected as the amine source for further exploration the reversible functionalization of the aldehyde groups embedded in the supramolecular polymers (Figure 7a). A model reaction between the aldehyde motor and hydroxylamine in aqueous media confirmed efficient oxime formation (Figure S41). The reaction proceeded without acid catalysis at room temperature, suggesting that mild conditions are suitable for investigating imine formation in aldehyde motor‐based supramolecular polymers. To explore the supramolecular chirality and morphology changes of the assemblies upon reaction with hydroxylamine, Cryo‐TEM, CD and UV–vis absorption measurements were performed at different stages. In the UV–vis spectra, upon the addition of NH_2_OH, the characterized absorption band at 365 nm belonging to the ***Z***‐**M1*_st_*** gradually decreased with the generation of a new band at 350 nm, indicating the formation of oxime ***Z***‐**M3*_st_*** (Figure 7b and Figure S42). We further characterized the transformations by using Fourier transform infrared (FTIR) spectroscopy. Upon completion of the reaction, the characteristic vibrational band of the aldehyde functional groups at 1678 cm^−1^ disappeared. Concurrently, new absorption bands emerged at 3600 and 950 cm^−1^, corresponding to O─H and N─O stretching vibrations of the oxime moiety, respectively (Figure S44, black curve), indicating the conversion of aldehyde groups to oxime functionalities. However, distinct from the gradual diminished absorptions in the CD spectra upon reaction of ***Z***‐**M1*_st_*** with bisulfite (vide supra), upon addition of hydroxylamine, ***Z***‐**M3*_st_*** displayed a negative Cotton effect in the CD spectra with about 20 nm blue shift corresponding with the change in UV–vis spectra, indicating the preservation of the ordered packing as well as the supramolecular chirality (Figure 7c). Cryo‐TEM measurements confirmed newly formed regular fibers with over 1 um length and about 7 nm in width, almost identical as those displayed by ***Z***‐**M1*_st_*** (Figure 7e and Figure S49). The supramolecular chirality and nearly identical morphologies indicated that NH_2_OH might react with the supramolecular polymers to facilitate the direct post‐functionalization of ***Z***‐**M1*_st_*** through oxime formation without undergoing disassembly processes. Additionally, the ***Z***‐**M3*_st_*** retained the light responsive properties in water after oxime modification. Upon light irradiation, the molecular motors underwent unidirectional rotation through different states with distinct chirality, inducing the morphological transformation from the nanofibers to the large aggregates and subsequent recovery after warming (Figures S45 and 46). These features closely parallel the photochemical responsiveness and aggregation behavior observed for the corresponding aldehyde motor (Figure 5 and Figure S50).

To get further insight into the transition process, we conducted CD spectra under similar conditions using varying amounts of hydroxylamine. In the CD spectra, regardless of the amount added, the diminishing signal of ***Z***‐**M1*_st_*** is consistently accompanied by an increasing signal of ***Z***‐**M3*_st_*** (Figure S43). As expected, the rise in ellipticity at 400 nm follows the same trend as the decline at 380 nm, indicating that the transformation of ***Z***‐**M1*_st_*** supramolecular polymers is directly associated with the formation of new supramolecular polymers composed of ***Z***‐**M3*_st_*** (Figure 7d). Although a disassembly mechanism cannot be excluded, the experimental data indicate the direct modification of the fibers without disrupting the chirality or altering the morphologies (Figure 7f), i.e., the supramolecular polymers seem to remain intact during the oxime formation. Moreover, the oxime motor ***Z***‐**M3*_st_*** demonstrated good modification reversibility, showing recover back to the aldehyde motor under acid‐catalysed hydrolysis both in monomeric and aggregated states (Figure 7f and Figures S47 and S48). These remarkable features open up new opportunities for the bottom‐up fabrication of non‐covalently driven supramolecular polymers with reversible chemically triggered modifications.

## Conclusion

In summary, a new design of multistate supramolecular polymers based on a molecular rotary motor has been developed for dynamic chirality control and dual responsiveness to light and chemical stimuli. Coupling light‐driven rotary motion and self‐assembly in water several distinct supramolecular states can be reached in a fully reversible manner. Taking advantage of the unidirectional rotation and multistate chirality of novel aldehyde‐functionalized motor, high efficiency in controlling multiple assemblies is demonstrated. The molecular chirality is transferred to achieve supramolecular chirality in a fully reversible self‐assembly process and the resulting chiral assemblies were successfully utilized to construct a multi‐responsive system capable of responding to both light and chemical stimuli. Upon irradiation with light, the supramolecular polymers exhibited multi‐state chirality, originating from the motion of the embedded molecular motor. When exposed to different nucleophiles, the polymers demonstrated either a reversible transformation from fibres to micelles or direct functionalization of the fibres, depending on the specific reaction pathways involving the aldehyde groups. Overall, this system successfully integrates orthogonal chemical/light responsiveness together with chirality in a single platform providing control of extended supramolecular systems via dynamic covalent modifications. The construction of these dynamic, multi‐responsive supramolecular polymers in water offers promising opportunities for future smart and adaptive materials.

## Supporting Information

The authors have cited additional references within the Supporting Information.^[^ ^71^, ^72^, ^73^, ^74^, ^75^, ^76^ ^]^

## Conflict of Interests

The authors declare no conflict of interest.

## Supporting information



Supporting Information

## Data Availability

The data that support the findings of this study are available in the supplementary material of this article.
