# Supplementary material for: Multi‐Stimulus Triggered Programmable Transformation of Molecular Motor Based Chiral Supramolecular Polymers in Water
Source: Angew Chem Int Ed Engl. 2026 Jan 9;65(7):e21360. doi: 10.1002/anie.202521360 (PMC12887618; doi:10.1002/anie.202521360)
Supplement: Supplementary file 1 — Supporting Information [file ANIE-65-e21360-s001.docx]

Multi-stimulus Triggered Programmable Transformation of Molecular Motor based Chiral Supramolecular Polymers in Water

Jinghao Wang^1^, Marc C. A. Stuart^2^, Ben L. Feringa^1^*

1. Stratingh Institute for Chemistry, University of Groningen, Nijenborgh 3, 9747 AG Groningen, The Netherlands

2. Groningen Biomolecular Sciences and Biotechnology Institute, University of Groningen, Nijenborgh 7, 9747 AG Groningen, The Netherlands

*To whom correspondence should be addressed.

E-mail: b.l.feringa@rug.nl (B.L.F.)

**Table of Content**

[**1.** **Materials and Methods** 3](#_Toc216097795)

[**2.** **Synthesis** 4](#_Toc216097796)

[**3.** **UV-vis spectroscopic study of the rotation of molecular motor M1 in methanol** 7](#_Toc216097797)

[**4.** **UV-vis spectroscopic study of the rotation of molecular motor M1 in water** 8](#_Toc216097798)

[**5.** **Eyring analysis of the thermal helix inversion of molecular motor M1** 9](#_Toc216097799)

[**6.** **^1^H NMR study of the photo-isomerization of molecular motor M1** 11](#_Toc216097800)

[**7.** **Temperature-dependent circular dichroism (CD) measurements** 13](#_Toc216097801)

[**8.** **Cryo-TEM analysis of the assembly and light-driven disassembly** 14](#_Toc216097802)

[**9.** **Quantum yield determination** 19](#_Toc216097803)

[**10.** **Model reaction of MOMe with NaHSO_3_** 24](#_Toc216097804)

[**11.** **UV-vis spectroscopic study of the reaction of molecular motors M1 with NaHSO_3_** 25](#_Toc216097805)

[**12.** **UV-Vis spectroscopic study of the recovery of molecular motors M1 with Na_2_CO_3_** 28](#_Toc216097806)

[**13.** **CD spectroscopic study of the reaction of molecular motors M1 with NaHSO_3_** 29](#_Toc216097807)

[**14.** **CD spectroscopic study of the recovery of molecular motors M1 with Na_2_CO_3_** 30](#_Toc216097808)

[**15.** **Rotary cycle of *Z-*M1_st_+NaHSO_3_ upon irradiation** 31](#_Toc216097809)

[**16.** **Cryo-TEM analysis of *Z*-M2_st_ and recovery** 33](#_Toc216097810)

[**17.** **Model reaction of MOMe with NH_2_OH** 34](#_Toc216097811)

[**18.** **UV-vis spectroscopic study of the reaction of molecular motors M1 with NH_2_OH** 35](#_Toc216097812)

[**19.** **CD spectroscopic study of the reaction of molecular motors M1 with NH_2_OH** 36](#_Toc216097813)

[**20.** **Fourier transform infrared spectroscopic study of *Z*-M1st and *Z*-M3st** 36](#_Toc216097814)

[**21.** **Rotary cycle of *Z-*M3_st_ upon irradiation** 37](#_Toc216097815)

[**22.** **Rotary cycle of *Z-*M3_st_ upon irradiation** 40](#_Toc216097816)

[**23.** **Cryo-TEM analysis of *Z*-M3_st_** 42](#_Toc216097817)

[**24.** **NMR and HRMS data** 44](#_Toc216097818)

[**25.** **Reference** 54](#_Toc216097819)

1. **Materials and Methods**

All chemicals were purchased from Sigma-Aldrich or TCI, and used without further purification. All the used organic solvents were analytically pure and dried or redistilled before use. The water (ULC/MS grade) used in sample preparation was purchased from Biosolve. Solvents used in the irradiation experiments were degassed by purging with argon for 30 min. For column chromatography, silica gel (Silicycles Siliaflash P60, 40–60 μm, 230–400 mesh) was used in all cases. Separation was carried out on silica gel 60 (silicon dioxide, SiO_2_; Merck, Germany) and kieselguhr F254 (Celite; Merck, Germany) for thin-layer chromatography (TLC), and visualization was achieved either by potassium permanganate (KMnO₄) staining or under UV light. All reactions were performed under an N_2_ atmosphere.

NMR spectra were recorded at 25 °C on Varian AMX400 (^1^H: 400 MHz, ^13^C: 101 MHz) and Varian Unity Plus (^1^H: 500 MHz, ^13^C: 125 MHz) spectrometers. Chemical shifts (*δ*) are expressed relative to the resonances of the residual non-deuterated solvent for ^1^H NMR [CDCl_3_: ^1^H(*δ*) = 7.26 ppm, CD_2_Cl_2_: ^1^H(*δ*) = 5.32 ppm] and ^13^C NMR [CDCl_3_: ^13^C(*δ*) = 77.2 ppm, CD_2_Cl_2_: ^13^C (*δ*) = 53.84 ppm]. Absolute values of the coupling constants are given in Hertz (Hz), regardless of their sign. Multiplicities are abbreviated as singlet (s), doublet (d), doublet of doublets (dd), triplet (t), triplet of doublets (td), quartet (q), multiplet (m). High-resolution mass spectrometry (HRMS) was performed on an LTQ Orbitrap XL spectrometer with ESI ionization. UV-vis spectra were recorded on a HewlettPackard HP 8543 spectrometer in a quartz cuvette with 1 cm path length. CD spectra were recorded on Jasco J-815 Circular Dichroism Spectrometer. IR spectra were measured on a PerkinElmer Spectrum 400. Cryo-TEM samples were placed on holey carbon-coated copper grids (Quantifoil 3.5/1, Quantifoil Micro Tools, Jena, Germany). Grids with samples were vitrified in liquid nitrogen (Vitrobot, FEI, Eindhoven, The Netherlands) and transferred to a FEI Talos Arctica cryo-electron microscope operating at 200 keV with a postcolumn energy filter (Gatan) in zero-loss mode, with a 20-eV slit. Irradiation experiments were performed using LED lamps (M365F1, Throlab) at 365 nm.

1. **Synthesis**


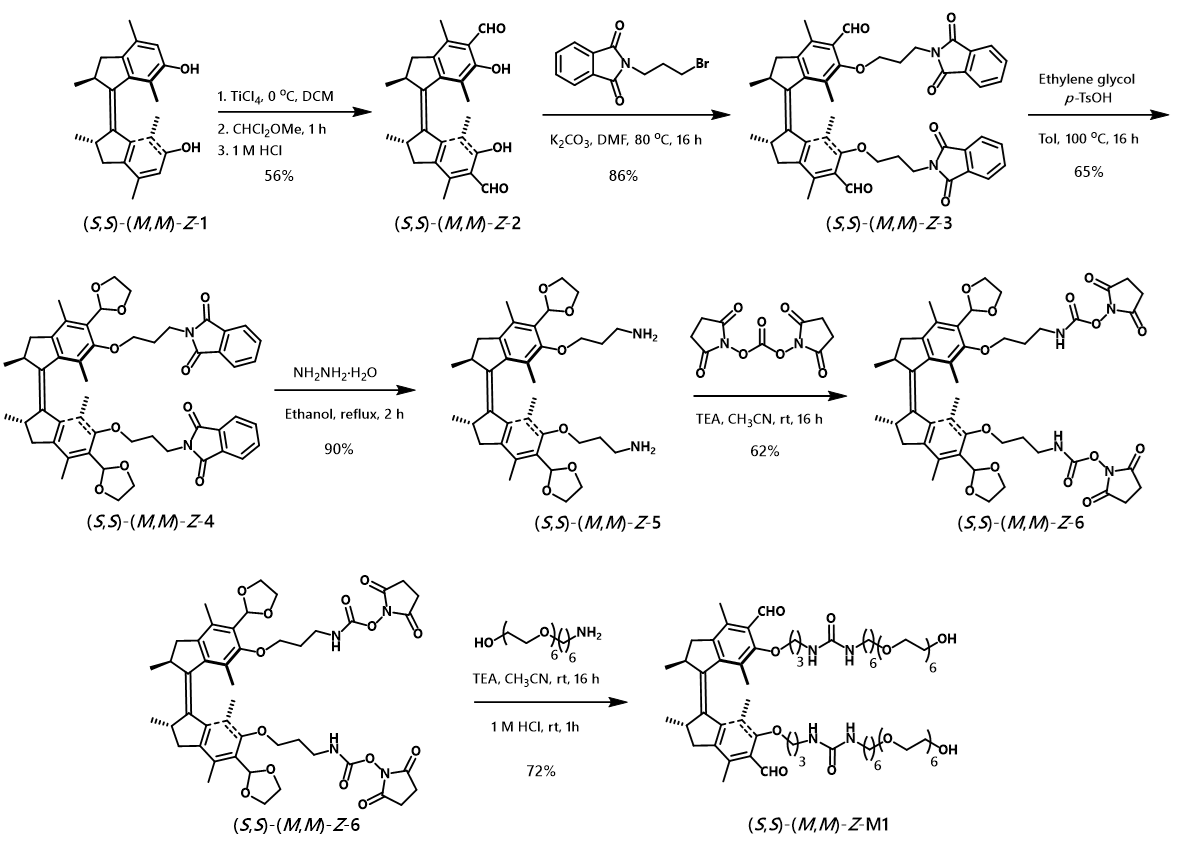


Figure **S1**. Synthesis scheme of (*S*,*S*)-(*M*,*M*)-***Z***-**M1**

(*S*,*S*)-(*M*,*M*)-***Z***-**2**

To a suspension of (*S*,*S*)-(*M*,*M*)-***Z***-**1**^[1]^ (209 mg, 0.6 mmol) in DCM (20 mL) at 0 ºC was slowly added TiCl_4_ (435.0 µL, 4.0 mmol). After stirring under a N_2_ atmosphere for 30 min, dichloro(methoxy)methane (543.0 µL, 6.0 mmol) was added to the suspension, which was subsequently allowed to warm up to room temperature. The mixture was stirring for another 45 min and then quenched with aq. 1 M HCl (10 mL). The aqueous phase was extracted with EtOAc (3*25 mL) and the combined organic layers were washed with brine, dried over Na_2_SO_4_ and concentrated under vacuo. The crude product was purified by column chromatography (SiO_2_, pentane:EtOAc = 20:1 to 10:1) to afford compound (*S*,*S*)-(*M*,*M*)-***Z***-**2** (137.6 mg, 0.34 mmol, 56%) as a yellow solid. ^1^H NMR (400 MHz, CDCl_3_) δ 12.47 (s, 2H), 10.29 (s, 2H), 3.39 (p, *J* = 6.8 Hz, 2H), 3.08 (dd, *J* = 14.6, 6.4 Hz, 2H), 2.53 – 2.48 (m, 8H), 1.37 (s, 6H), 1.10 (d, *J* = 6.8 Hz, 6H). ^13^C NMR (101 MHz, CDCl_3_) δ 194.48, 161.50, 149.25, 144.34, 134.60, 132.96, 122.33, 116.72, 41.40, 37.87, 20.26, 13.82, 13.61. HRMS (ESI pos) calcd. for [M+Na]^+^: 427.18798, found: 427.18847.

(*S*,*S*)-(*M*,*M*)-***Z***-**3**

Under a N_2_ atmosphere, to a suspension of (*S*,*S*)-(*M*,*M*)-***Z***-**2** (80.8 mg, 0.2 mmol) in dry DMF (10 mL) was added 2-(3-bromopropyl) isoindoline-1,3-dione (160.8 g, 0.6 mmol) and K_2_CO_3_ (165.6 g, 1.2 mmol). The mixture was stirred at 80 ºC overnight followed by concentrating in vacuo. To the concentrated mixture was added 20 mL deionized water. The aqueous phase was extracted with EtOAc and the combined organic layers were washed with brine, dried over Na_2_SO_4,_ and concentrated under reduced pressure. The crude product was purified by column chromatography (SiO_2_, pentane:EtOAc = 5:1) to afford compound (*S*,*S*)-(*M*,*M*)-***Z***-**3** (134.0 mg, 0.17 mmol, 86%) as a yellow solid. ^1^H NMR (400 MHz, CDCl_3_) δ 10.51 (s, 2H), 7.78 (dd, *J* = 5.4, 3.0 Hz, 4H), 7.68 (dd, *J* = 5.5, 3.0 Hz, 4H), 3.99 (dt, *J* = 9.5, 6.3 Hz, 2H), 3.91 – 3.83 (m, 6H), 3.39 (p, *J* = 6.7 Hz, 2H), 3.10 (dd, *J* = 14.9, 6.3 Hz, 2H), 2.55 (d, *J* = 14.9 Hz, 2H), 2.50 (s, 6H), 2.19 (dq, *J* = 7.8, 6.3 Hz, 4H), 1.47 (s, 6H), 1.08 (d, *J* = 6.7 Hz, 6H). ^13^C NMR (101 MHz, CDCl_3_) δ 193.08, 168.17, 161.13, 147.40, 143.67, 141.44, 133.92, 133.83, 132.05, 126.71, 126.49, 123.22, 72.90, 41.61, 38.54, 35.23, 29.23, 20.24, 16.50, 14.41. HRMS (ESI pos) calcd. for [M+Na]^+^: 801.31464, found: 801.31446.

(*S*,*S*)-(*M*,*M*)-***Z***-**4**

Under a N_2_ atmosphere, to a suspension of (*S*,*S*)-(*M*,*M*)-***Z***-**3** (155.6 mg, 0.2 mmol) in dry toluene (20 mL) was added ethylene glycol (124 mg, 2.0 mmol) and a catalytic amount of *p*-toluenesulfonic acid monohydrate (3.8 mg, 0.02 mmol), and the mixture was stirring at 100 ºC for 16 h. The reaction was quenched with water (20 mL) and the mixture was extracted with EtOAc (3 × 25 mL). The combined organic layers were washed with brine, dried over Na_2_SO_4_ and concentrated under reduced pressure. The crude product was purified by column chromatography (SiO_2_, pentane:EtOAc = 4:1) to afford compound (*S*,*S*)-(*M*,*M*)-***Z***-**4** (112.8 mg, 0.13 mmol, 65%) as a yellow solid. ^1^H NMR (400 MHz, CD_2_Cl_2_) δ 7.78 (dd, *J* = 5.5, 3.1 Hz, 4H), 7.70 (dd, *J* = 5.6, 3.1 Hz, 4H), 6.15 (s, 2H), 4.15 –4.10 (m, 4H), 4.06 – 3.98 (m, 4H), 3.90 – 3.70 (m, 8H), 3.35 (p, *J* = 6.7 Hz, 2H), 3.06 (dd, *J* = 14.8, 6.3 Hz, 2H), 2.48 (d, *J* = 14.8 Hz, 2H), 2.30 (s, 6H), 2.11 (q, *J* = 7.3 Hz, 4H), 1.44 (s, 6H), 1.06 (d, *J* = 6.7 Hz, 6H). ^13^C NMR (101 MHz, CD_2_Cl_2_) δ 168.11, 156.10, 143.24, 141.79, 141.46, 133.80, 132.39, 132.19, 125.85, 124.81, 122.93, 100.58, 71.72, 65.01, 64.99, 41.63, 38.69, 35.34, 29.08, 20.01, 15.05, 14.54. HRMS (ESI pos) calcd. for [M+H]^+^: 867.38513, found: 867.38432.

(*S*,*S*)-(*M*,*M*)-***Z***-**5**

Under a N_2_ atmosphere, to a suspension of (*S*,*S*)-(*M*,*M*)-***Z***-**4** (86.7 mg, 0.1 mmol) in ethanol (20 mL) was added hydrazine hydrate (50–60%, 15.4 mmol, 0.9 mL), following by heating the mixture at reflux for 2 h. After cooling and concentrating in vacuo, the mixture was dissolved in 15% aq. NaOH (20 mL), and extracted with DCM. The combined organic layers were washed with brine, dried over Na_2_SO_4_ and concentrated under vacuo to afford (*S*,*S*)-(*M*,*M*)-***Z***-**5** (55 mg, 0.09 mmol, 90%) as a yellow solid. ^1^H NMR (400 MHz, CDCl_3_) δ 6.19 (s, 2H), 4.29 – 4.00 (m, 8H), 3.87 – 3.70 (m, 4H), 3.31 (p, *J* = 6.7 Hz, 2H), 3.03 (dd, *J* = 14.7, 6.3 Hz, 2H), 2.87 (td, *J* = 6.9, 1.7 Hz, 4H), 2.45 (d, *J* = 14.7 Hz, 2H), 2.32 (s, 6H), 1.85 (p, *J* = 6.6 Hz, 6H), 1.40 (s, 6H), 1.03 (d, *J* = 6.8 Hz, 6H). ^13^C NMR (101 MHz, CDCl_3_) δ 156.18, 143.38, 141.71, 141.35, 132.13, 126.01, 124.31, 100.96, 72.16, 65.11, 41.52, 39.45, 38.83, 34.00, 20.90, 20.33, 15.34, 14.74. HRMS (ESI pos) calcd. for [M+Na]^+^: 629.35611, found: 629.35844.

(*S*,*S*)-(*M*,*M*)-***Z***-**6**

Under a N_2_ atmosphere, to a suspension of (*S*,*S*)-(*M*,*M*)-***Z***-**5** (61 mg, 0.1 mmol) in acetonitrile (20 mL) was added *N*,*N*′-disuccinimidyl carbonate (30.7 mg, 0.12 mmol) and triethylamine (16.5 μL, 0.12 mmol), following by stirring the mixture for 16 h. The reaction was quenched with water (20 mL) and the mixture was extracted with EtOAc (3 × 25 mL). The combined organic layers were washed with brine, dried over Na_2_SO_4_ and concentrated under vacuo. The crude product was purified by column chromatography (SiO_2_, DCM:MeOH = 20:1) to afford compound (*S*,*S*)-(*M*,*M*)-***Z***-**6** (55.2 mg, 0.06 mmol, 62%) as a yellow solid. ^1^H NMR (401 MHz, CD_2_Cl_2_) δ 6.11 (m, 3H), 4.22 – 3.98 (m, 8H), 3.82 (ddt, *J* = 37.4, 9.8, 5.9 Hz, 4H), 3.39 (m, 6H), 3.08 (dd, *J* = 14.8, 6.4 Hz, 2H), 2.78 (s, 8H), 2.49 (d, *J* = 14.8 Hz, 2H), 2.31 (s, 6H), 1.98 (m, 4H), 1.43 (s, 6H), 1.06 (d, *J* = 6.7 Hz, 6H). ^13^C NMR (101 MHz, CD_2_Cl_2_) δ 170.06, 155.75, 151.54, 143.35, 141.80, 141.57, 132.49, 125.71, 124.75, 105.00, 100.66, 71.73, 65.09, 65.03, 41.56, 39.92, 38.74, 29.51, 25.46, 19.98, 15.13, 14.53. HRMS (ESI pos) calcd. for [M+H]^+^: 889.38658, found: 889.38676.

(*S*,*S*)-(*M*,*M*)-**M1**

To a solution of (*S*,*S*)-(*M*,*M*)-***Z***-**6** (50 mg, 0.056 mmol) in acetonitrile (10 mL) was added triethylamine (16.5 μL, 0.12 mmol) and compound **1**^[2]^ (46 mg, 0.12 mmol). After stirring for 16 h at room temperature, the mixture was quenched with water (10 mL), followed by extraction with EtOAc. The organic layer was dried with Na_2_SO_4_ and concentrated *in vacuo*. The resulting viscous oil was dissolved in acetonitrile (5 mL) and aq. 1 M HCl solution was added dropwise into the mixture (16.5 μL, 0.12 mmol). After stirring at room temperature for 1 h, the mixture was quenched with an aq. NaHCO_3_ solution and the mixture was extracted with EtOAc (3 × 25 mL). The combined organic layer was washed with brine, dried over Na_2_SO_4_ and concentrated under vacuo. The crude product was purified by column chromatography (SiO_2_, DCM:MeOH = 10:1) to afford compound (*S*,*S*)-(*M*,*M*)-***Z***-**M1** (76.0 mg, 0.06 mmol, 72% over 2 steps) as a yellow solid. ^1^H NMR (401 MHz, CD_2_Cl_2_) δ 10.48 (s, 2H), 5.74 (s, 2H), 5.42 (s, 2H), 4.02 – 3.81 (m, 4H), 3.73 – 3.48 (m, 55H), 3.41 (q, *J* = 6.2 Hz, 7H), 3.27 (s, 4H), 3.16 – 2.93 (m, 8H), 2.56 (d, *J* = 14.9 Hz, 2H), 2.48 (s, 6H), 1.97 (t, *J* = 6.5 Hz, 5H), 1.54 (t, *J* = 6.9 Hz, 5H), 1.07 (d, *J* = 6.7 Hz, 6H). ^13^C NMR (101 MHz, CD_2_Cl_2_) δ 192.87, 161.10, 147.49, 143.72, 141.42, 133.57, 126.69, 126.46, 124.82, 72.82, 72.44, 71.10, 70.47, 70.36, 70.32, 70.12, 69.93, 61.43, 41.59, 40.01, 38.45, 37.07, 30.86, 30.17, 29.48, 26.61, 25.78, 19.88, 16.16, 14.21. HRMS (ESI pos) calcd. for [M+Na]^+^: 1355.80752, found: 1355.80715.

1. **UV-vis spectroscopic study of the rotation of molecular motor M1 in methanol**


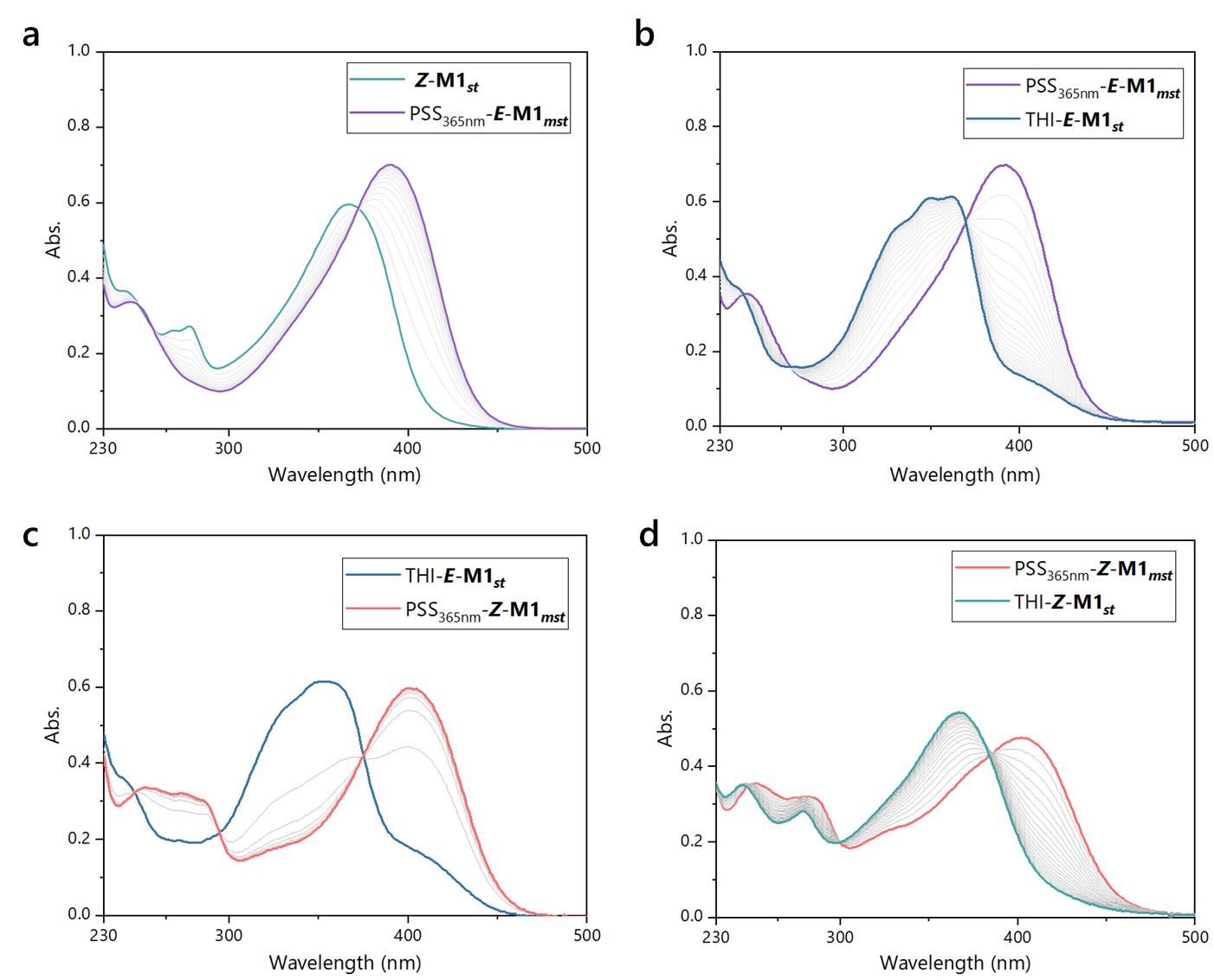


Figure **S2**. UV-vis absorption spectra of ***Z***-**M1_st_** (30 μM) in MeOH (a) upon 365 nm light irradiation for 2 min at −15 ºC to form PSS_365nm_-***E***-**M1_mst_**, (b) maintaining the solution in the dark at −15 ºC for 1 h to reach THI-***E***-**M1_st_**, (c) subsequent irradiating with 365 nm light at −15 ºC for 1 min to yield PSS_365nm_-***Z***-**M1_mst_**, (d) finally keeping in the dark at 55 ºC for 5 h to recover THI-***Z***-**M1_st_**.

1. **UV-vis spectroscopic study of the rotation of molecular motor M1 in water**


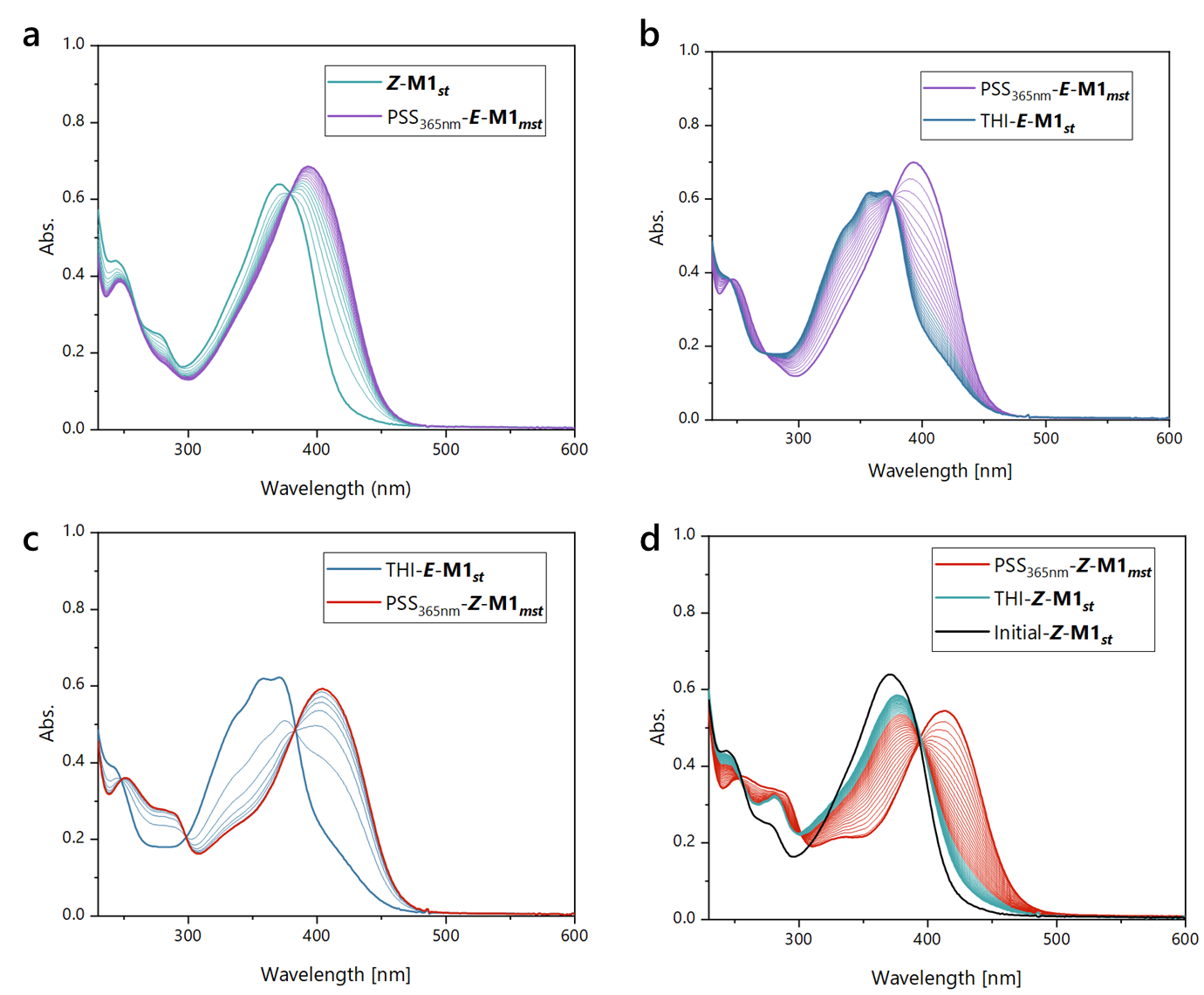


Figure **S3**. UV-vis absorption spectra of ***Z***-**M1_st_** (30 μM) in water. (a) upon 365 nm light irradiation for 2 min at 5 ºC to form PSS_365nm_-***E***-**M1_mst_**, (b) maintaining the solution in the dark at 5 ºC for 2 h to reach THI-***E***-**M1_st_**, (c) subsequent irradiating with 365 nm light at 5 ºC for 1 min to yield PSS_365nm_-***Z***-**M1_mst_**, (d) finally keeping in the dark at 70 ºC for 5 h to recover THI-***Z***-**M1_st_**.

1. **Eyring analysis of the thermal helix inversion of molecular motor M1**


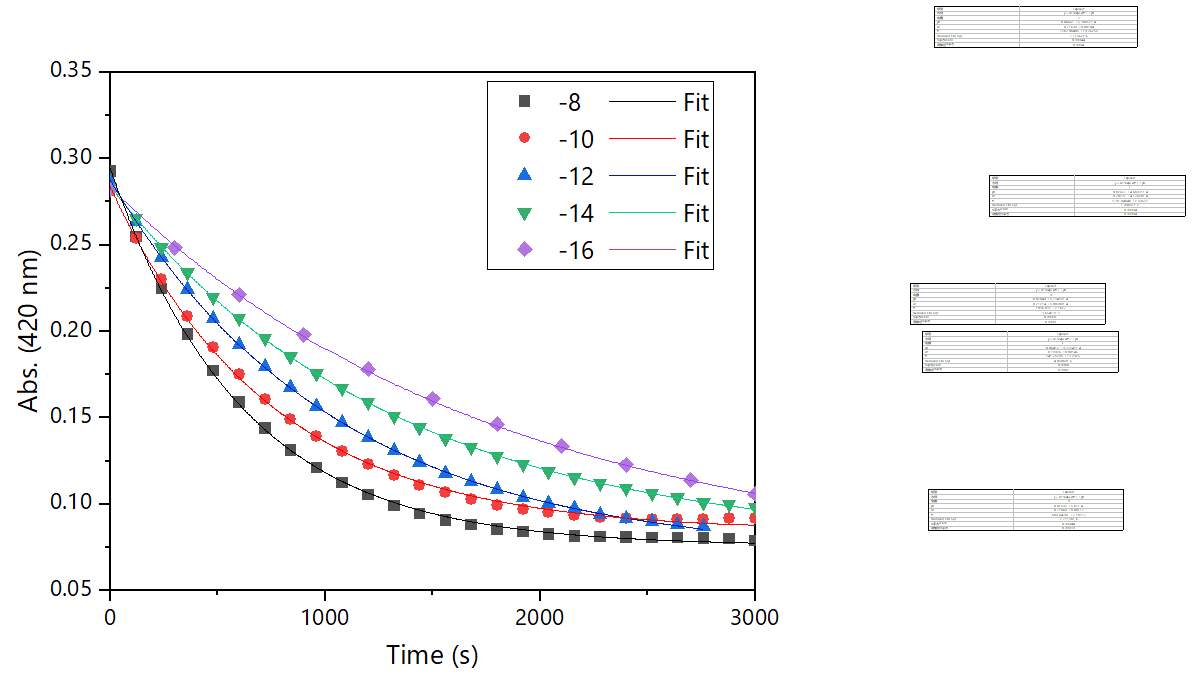

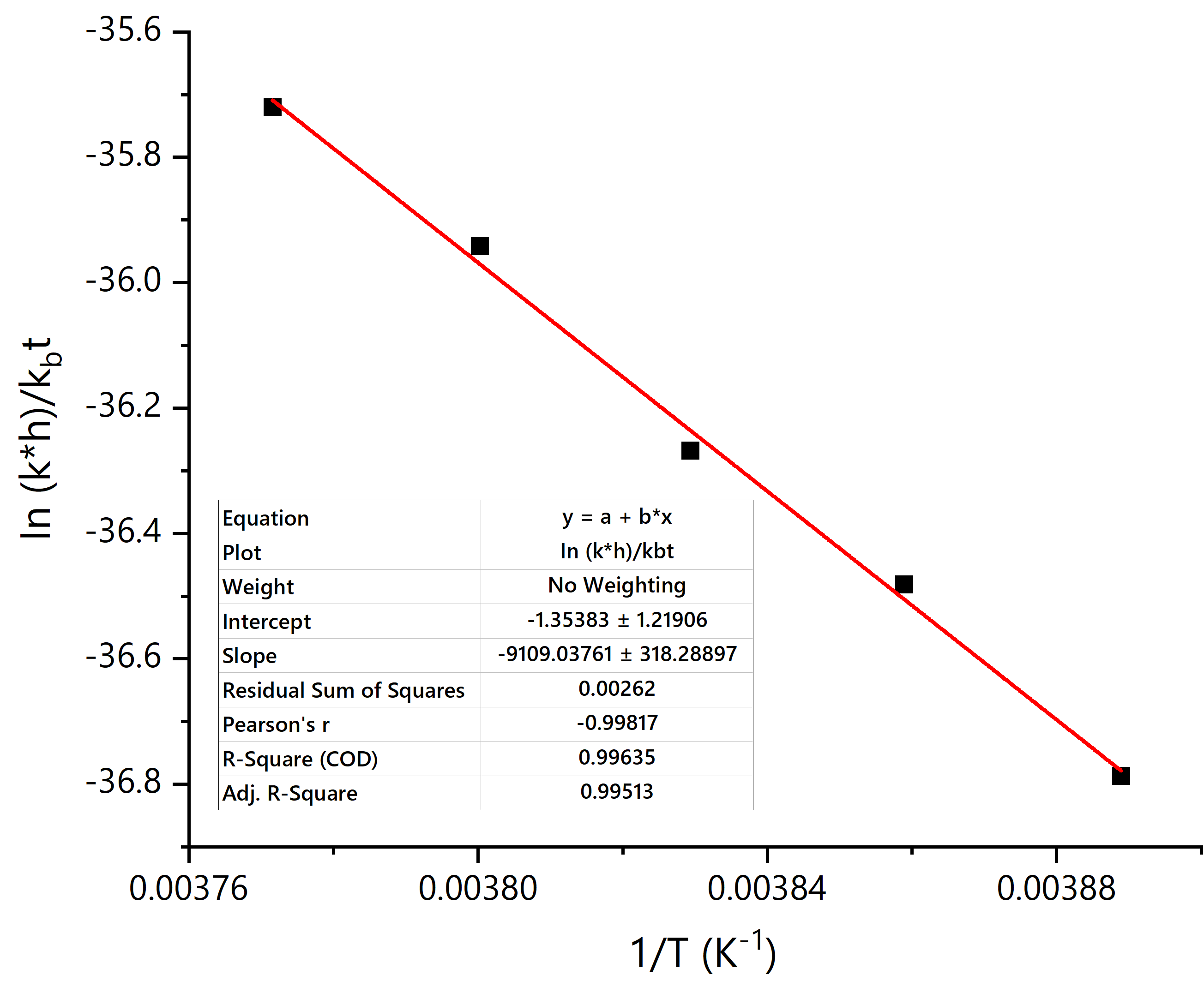


Figure **S4**. (a) Time-dependent absorption changes at 420 nm during the THI of ***E***-**M1_mst_** in methanol at different temperatures (ºC). (b) Eyring analysis with a linear fit of the THI of ***E***-**M1_mst_** in methanol.


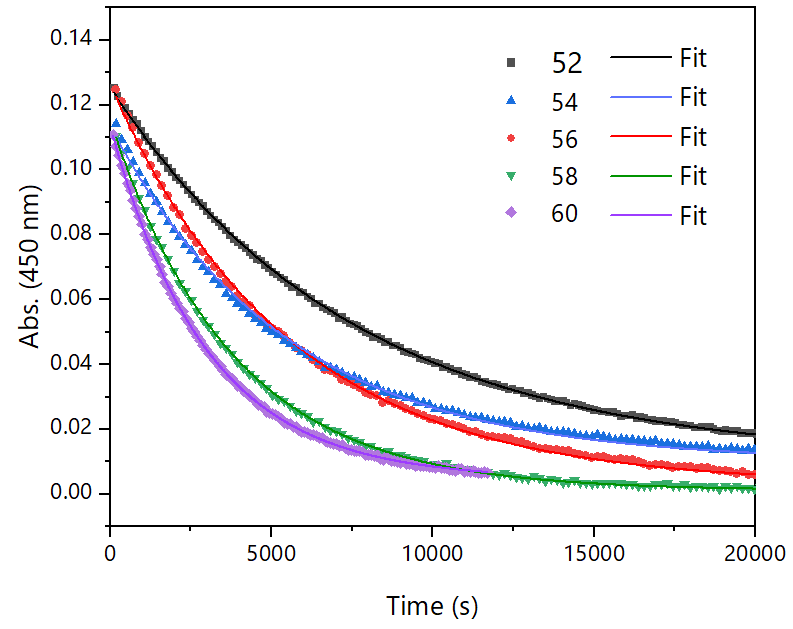

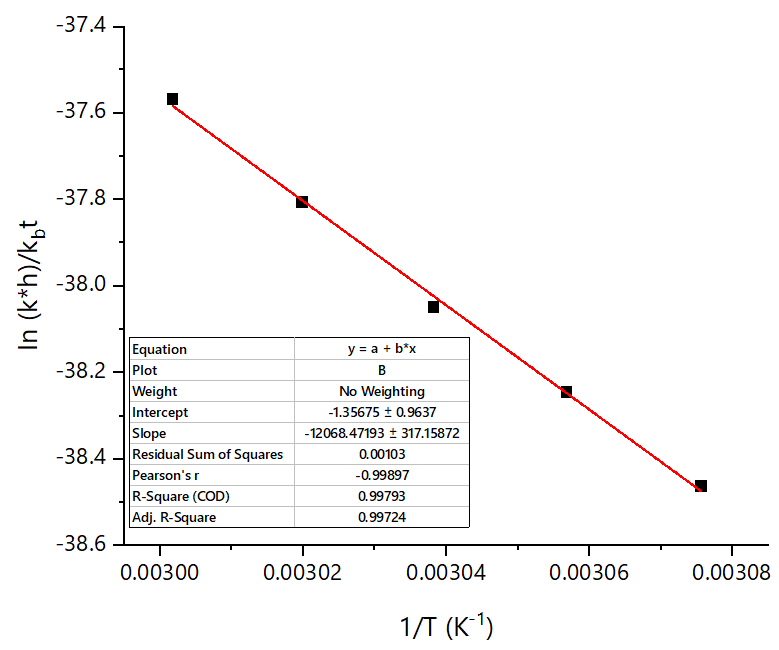


Figure **S5**. (a) Time-dependent absorption changes at 450 nm during the THI of ***Z***-**M1_mst_** in methanol at different temperatures (ºC). (b) Eyring analysis with a linear fit of the THI of ***Z***-**M1_mst_** in methanol.


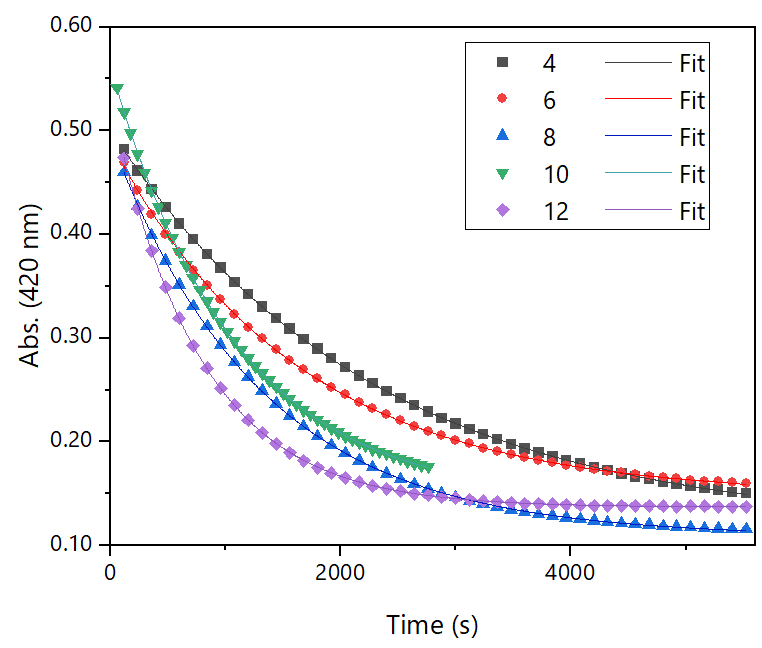

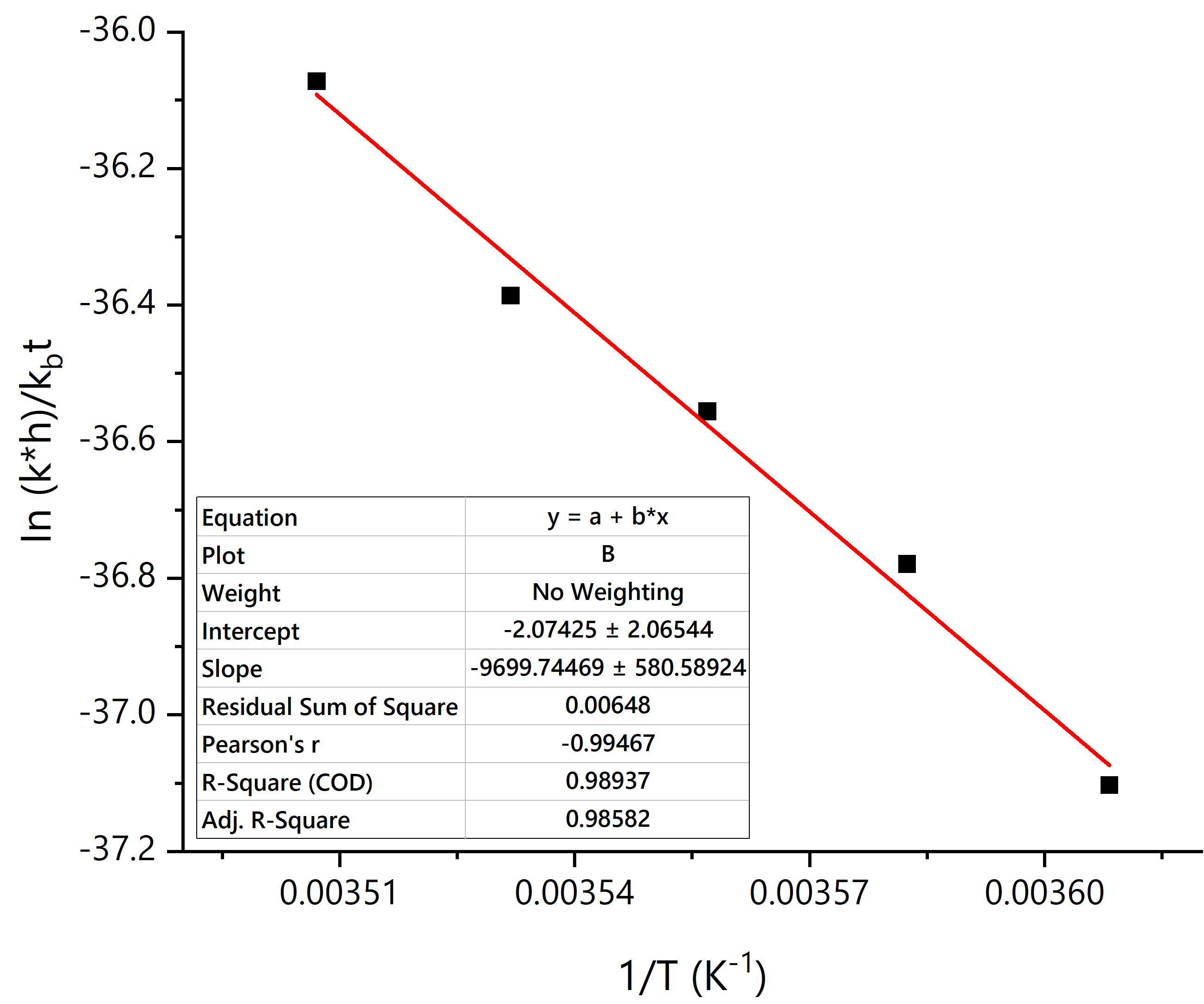


Figure **S6**. (a) Time-dependent absorption changes at 420 nm during the THI of ***E***-**M1_mst_** in water at different temperatures (ºC). (b) Eyring analysis with a linear fit of the THI of ***E***-**M1_mst_** in water.


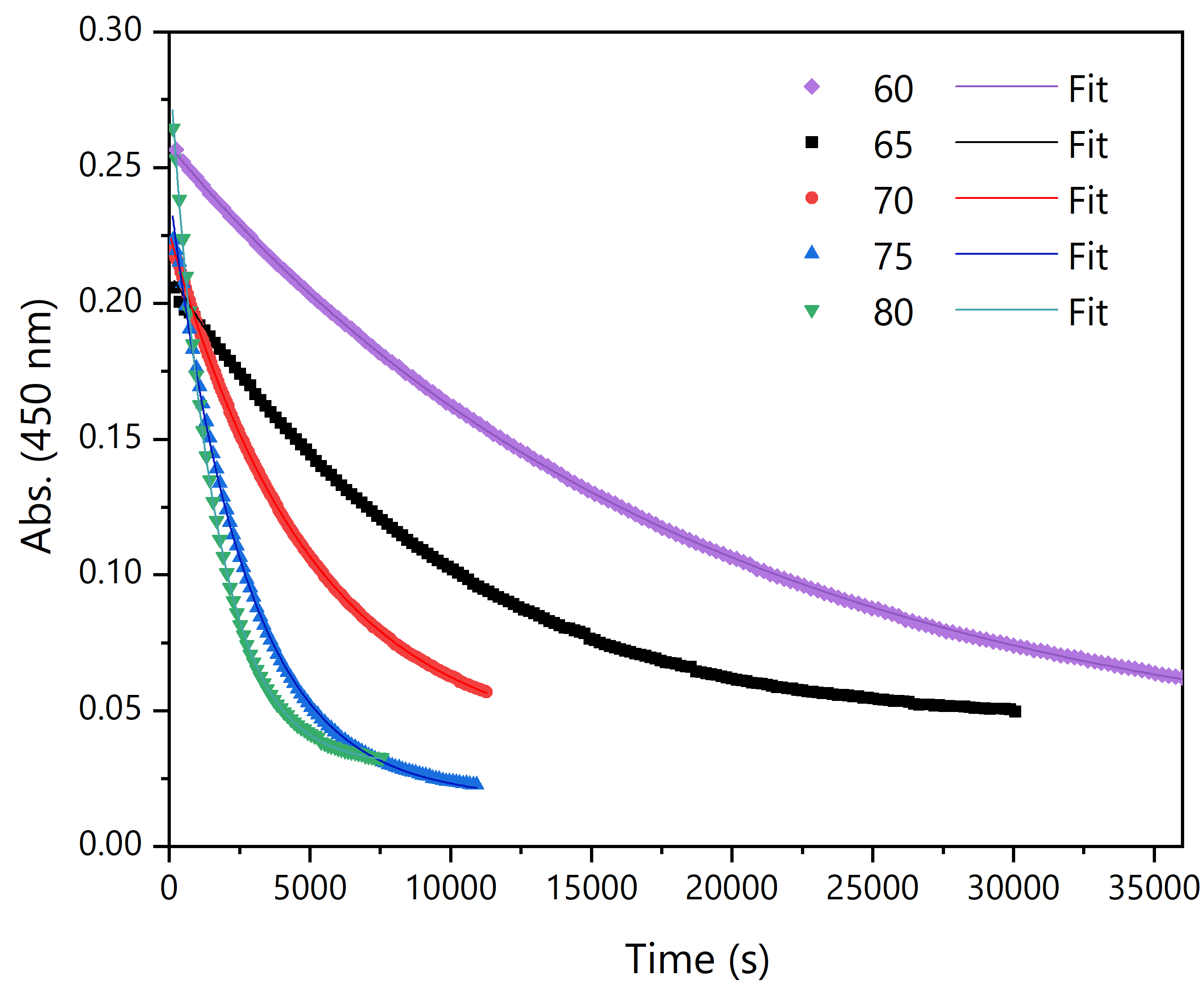

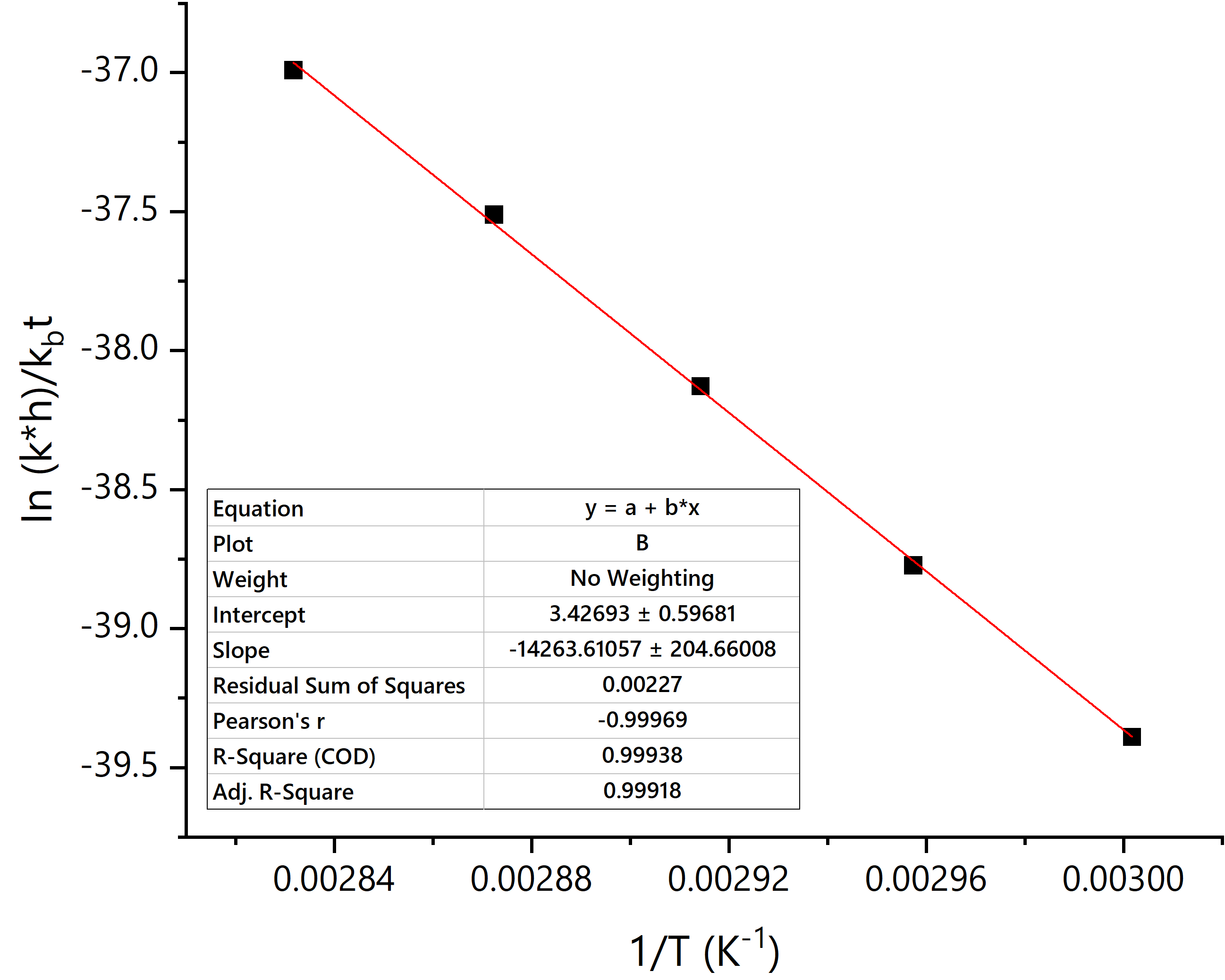


Figure **S7**. (a) Time-dependent absorption changes at 450 nm during the THI of ***Z***-**M1_mst_** in water at different temperatures (ºC). (b) Eyring analysis with a linear fit of the THI of ***Z***-**M1_mst_** in water.


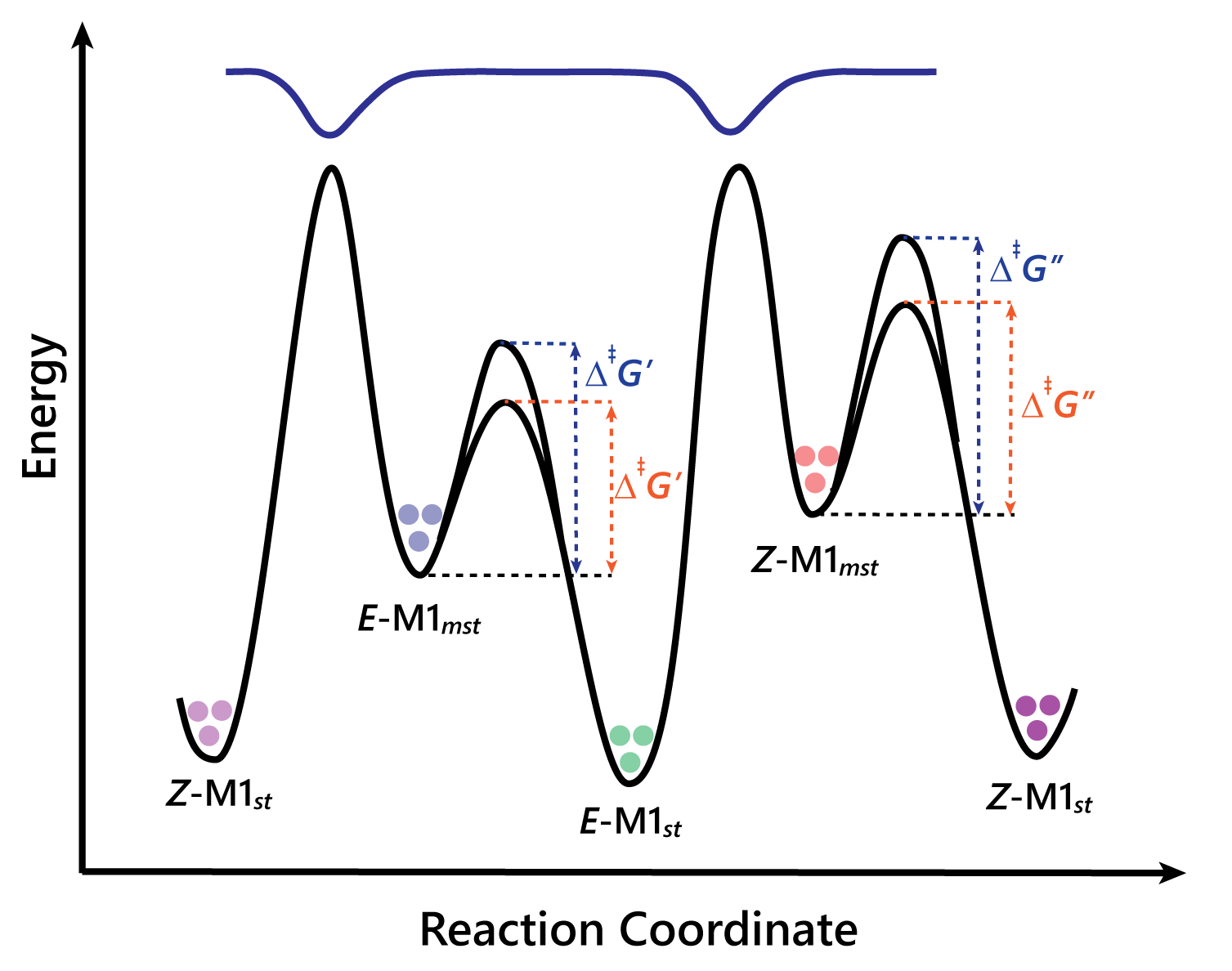


Figure **S8**. Energy diagram of the rotation of molecular motor in methanol (orange) and water (blue), respectively.^[3]^

Table **S1**. Gibbs free energy of activation (Δ^‡^*G*) and corresponding thermal half-lives (t_1/2_) of helix inversion of **M1** and CM motor.^[1]^ (Δ^‡^*G'* for ***E***_mst_ to ***E***_st_ and *Δ^‡^G''* for ***Z***_mst_ to ***Z***_st_; t*'*_1/2_ for ***E***_mst_ to ***E***_st_ and t*''*_1/2_ for ***Z***_mst_ to ***Z***_st_, respectively, defined as ln(2)/k at 20 °C).

|  | Δ^‡^*G'* (kJ/mol) | t*'*_1/2_ (sec) | Δ^‡^*G''* (kJ/mol) | t*''*_1/2_ (h) |
| --- | --- | --- | --- | --- |
| **M1**-methanol | 79.1 | 13.8 | 104 | 94 |
| **M1**-water | 85.7 | 210 | 110 | 1403 |
| **CM**-water | 85.0 | 156 | 109.6 | 1068 |

1. **^1^H NMR study of the photo-isomerization of molecular motor M1**

Due to the formation of supramolecular polymers in water, precisely quantifying the conversion at each step of the isomerization of **M1** in D_2_O from ^1^H NMR has been demonstrated not possible.^[2]^ Therefore, we have measured the isomers as the result of the photo-isomerization processes by freezing-drying the assembly in water and re-dissolving in methanol. The nearly quantitive conversion of ***Z***-**M1_st_** to ***E***-**M1_st_** after THI (thermal helix inversion) was demonstrated clearly by the disappearance of the signals belonging to the ***Z***-**M1**_st_ in ^1^H NMR spectra (Figure S9a, b).^[3]^ The samples after the secondary photoisomerization step were revealed as the mixture of ***Z***-**M1_mst_** and ***E***-**M1_st_** with a ratio of 86:14 obtained by integration (Figure S9c, S10a). After warming for 5 h, the NMR results displayed a ratio of the newly generated Re-***Z***-**M1**_st_ and ***E***-**M1**_st_, which remained similar i. e. 87:13 (Figure S9d, S10b), suggesting a fully conversion of ***Z***-**M1**_mst_ to ***Z***-**M1**_st_ in water during the THI steps. The results from the NMR study are consistent with those from the CD measurements.


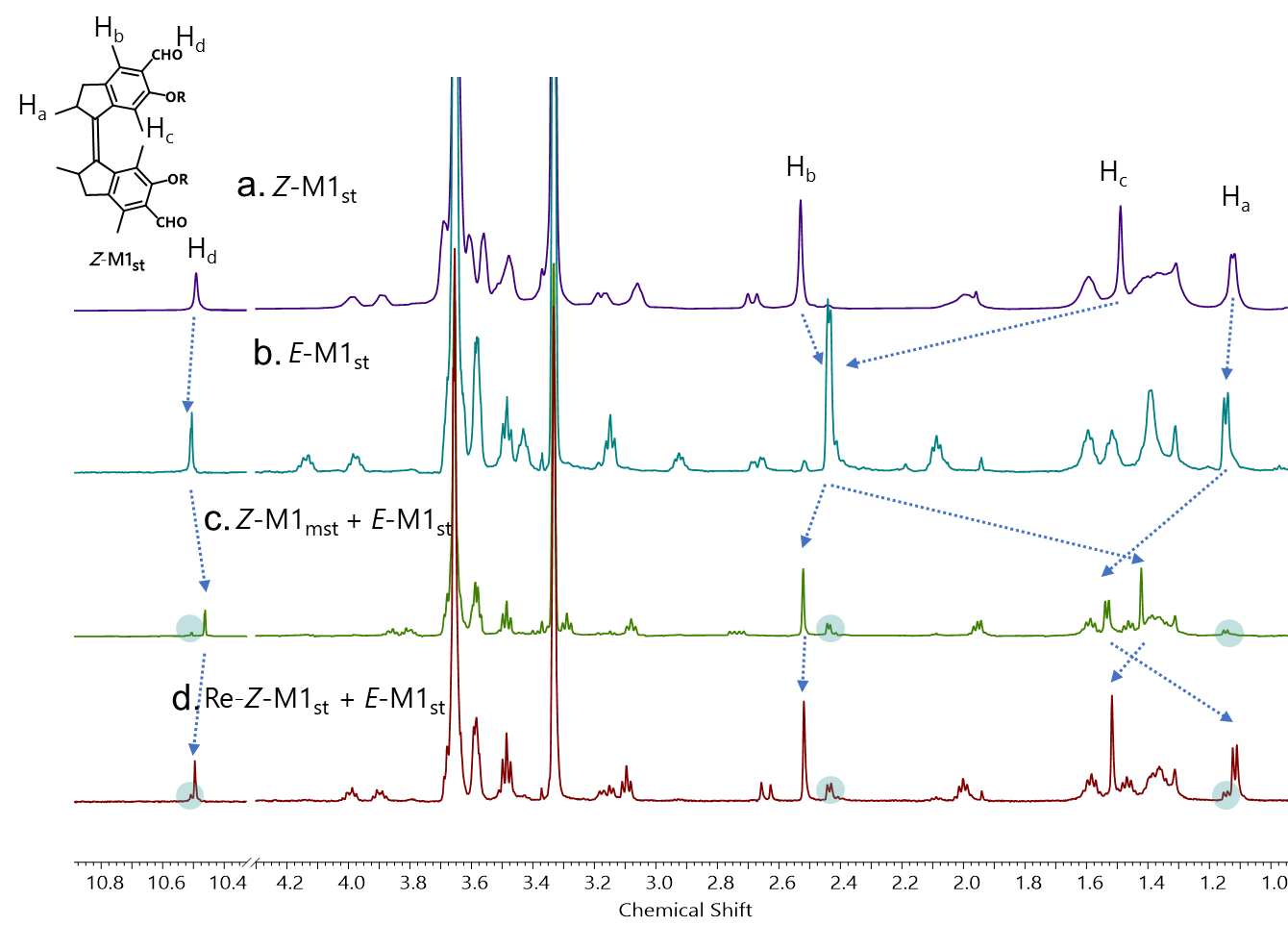


Figure **S9**. ^1^H NMR spectra (CD3OD, 298 K, 500 MHz) of (a) stable ***Z***-**M1_st_**, (b) after irradiation with 365 nm light for 2 min and subsequently maintaining the sample in the dark at room temperature for 10 min in water to get stable ***E***-**M1_st_**, (c) followed by irradiating with 365 nm light for 1 min to reach a PSS mixture of ***Z***-**M1_mst_** and ***E***-**M1_st_** with a ratio of 87:13, (d) finally keeping the sample in the dark at 55 ºC for 5 h to achieve the THI of ***Z***-**M1_mst_** to **Re**-***Z***-**M1_st_**. The compound in the samples were characterized after removal of water by freeze-drying and re-dissolving them in CD3OD.


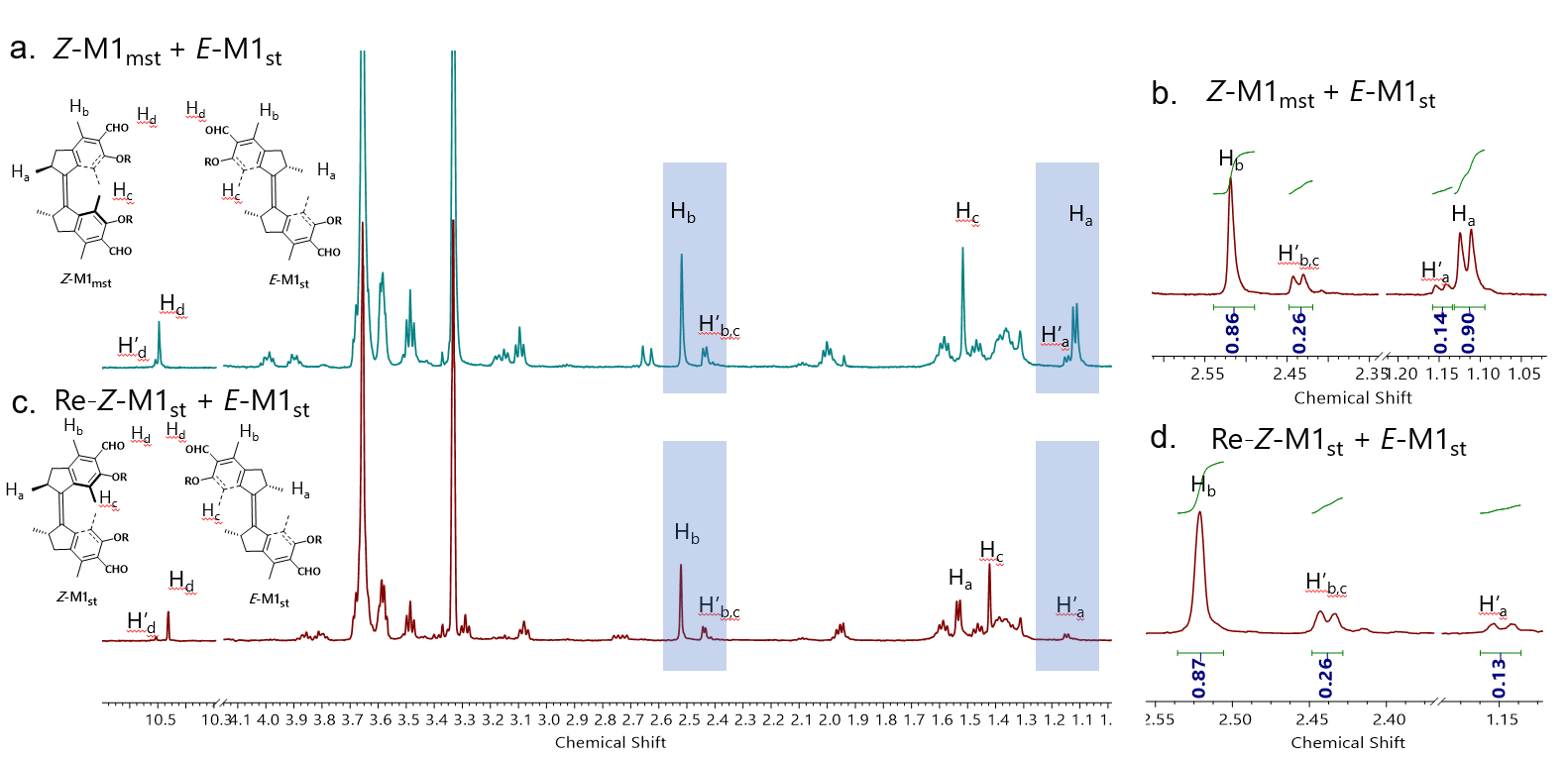


Figure **S10**. (a) ^1^H NMR spectra (CD3OD, 298 K, 500 MHz) of the PSS mixture of ***Z***-**M1_mst_** and ***E***-**M1_st_**, with the characteristic peaks labeled (highlighted with a blue background), (b) integration of the signals from ***Z***-**M1_mst_** and ***E***-**M1_st,_** indicating a ratio of 86:14 (***Z***: ***E***), (c) ^1^H NMR spectra (CD3OD, 298 K, 500 MHz) of the mixture of ***Z***-**M1_mst_** and ***E***-**M1_st_** after THI process, with the characteristic peaks labeled (highlighted with a blue background), (b) integration of the signals after THI**_,_** with a Re-***Z***-**M1_st_** to ***E***-**M1_st_** ratio of 87:13 **_._**

1. **Temperature-dependent circular dichroism (CD) measurements**

In order to investigate the polymerization mechanism, we monitored the molar ellipticity at 400 nm relative to the formation of helical aggregates upon heating ***Z***-**M1_st_** (35 μM, aqueous solution) from 298 K to 353 K (Figure S11). In the current system, no obvious LCST phenomenon was observed up to 353 K. This absence of the LCST-driven phase transition allows the system to remain within a well-defined thermodynamic regime over the investigated temperature range. Increasing the temperature, the CD signal for helical aggregates decreased gradually, suggesting an increase in the disorder of the helical packing of the molecules. Here, we have calculated the temperature-dependent degree of aggregation (*α*_agg_) based on the molar ellipticity data and found that the supramolecular polymerization of ***Z***-**M1_st_** in water fits more closely with the isodesmic model than with the cooperative models (Figure 3c). ^[4-6]^

Figure **S11**. Temperature-dependent molar ellipticity (*ϴ*) at 310 nm upon heating an aqueous solution of the supramolecular polymer formed by ***Z***-**M1_st_** (35 μM in water) from 293 K to 353 K.

1. **Cryo-TEM analysis of the assembly and light-driven disassembly**

Sample preparation: The colloidal suspension of ***Z***-**M1_st_** was obtained by dissolving the sticky solids into MilliQ water using ultrasound to be dispersed and kept in the dark at room temperature overnight. At different concentrations, the supramolecular polymers exhibit a homogeneous nanofiber structure with a radius of about 7 nm (Figure 3a, 4e, S12, 13). All irradiations were performed with a Thermolab UV lamp (λ = 365 nm) positioned 2 cm from the sample with the collimator. The sample was first irradiated with 365 nm light for 2 min at 5 ºC and kept in the dark at room temperature for 60 min to obtain ***E***-**M1_st_** in a 1 mm path length quartz cuvette (Figure 4f, S9b, 14). The sample was then irradiated with 365 nm light for 1 min to obtain a PSS mixture of ***Z***-**M1_mst_** and ***E***-**M1_st_** with a ratio of 86:14. (Figure 4g, S15). Finally, the sample was kept in the dark at 55 ºC for 6 h to get a mixture of ***Z***-**M1_st_** and ***E***-**M1_st_** in a ratio of 86:14 (Figure S16). The ratio of isomers was determined by integrating the NMR signals (Figure S10). A few microliters of each sample solution were placed on holey carbon-coated copper grids (Quantifoil 3.5/1, Quantifoil Micro Tools, Jena, Germany). Grids with samples were vitrified in liquid nitrogen (Vitrobot, FEI, Eindhoven, The Netherlands) and transferred to a FEI Talos Arctica cryo-electron microscope operating at 200 keV with a postcolumn energy filter (Gatan) in zero-loss mode, with a 20-eV slit.


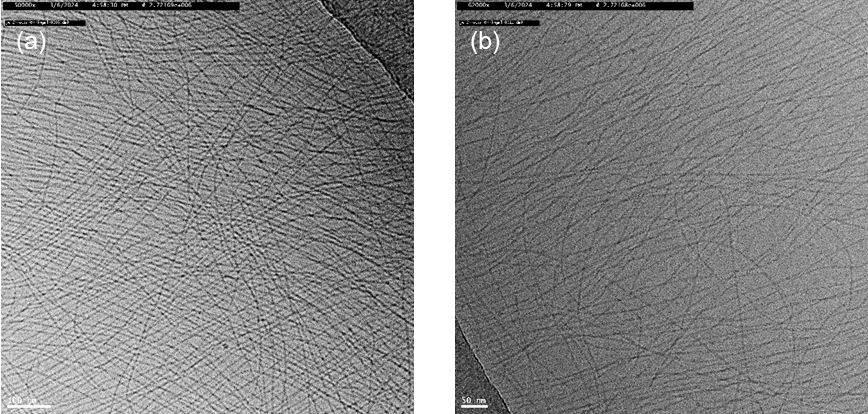


Figure **S12**. Cryo-TEM images of the ***Z***-**M1_st_** (5mg/ml) in water.


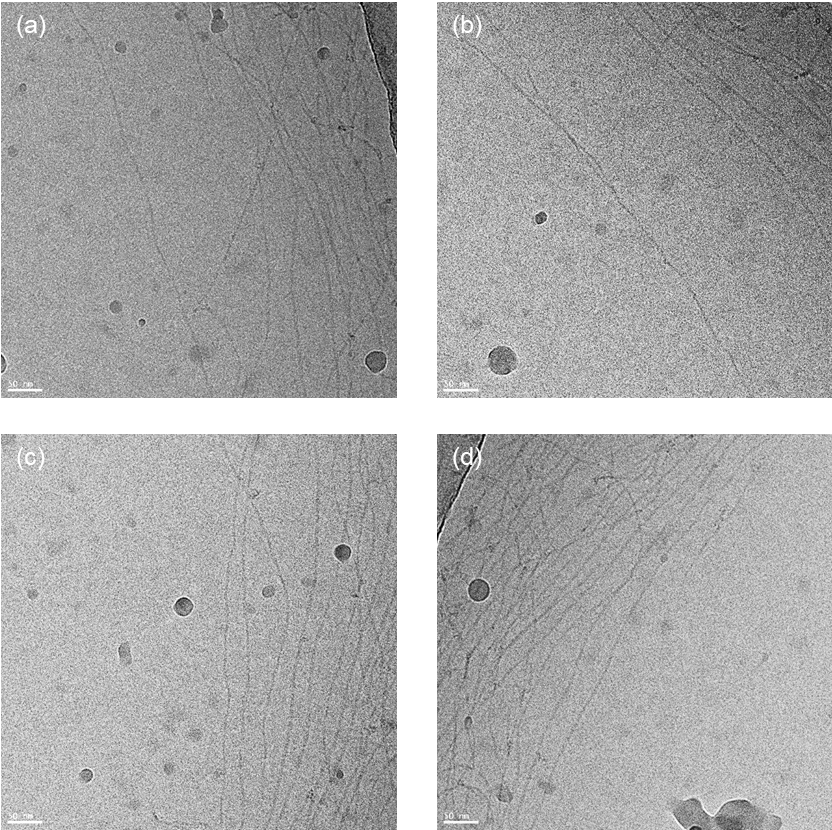


**Figure S13**. Cryo-TEM images of the ***Z***-**M1_st_** (1mg/ml) in water.


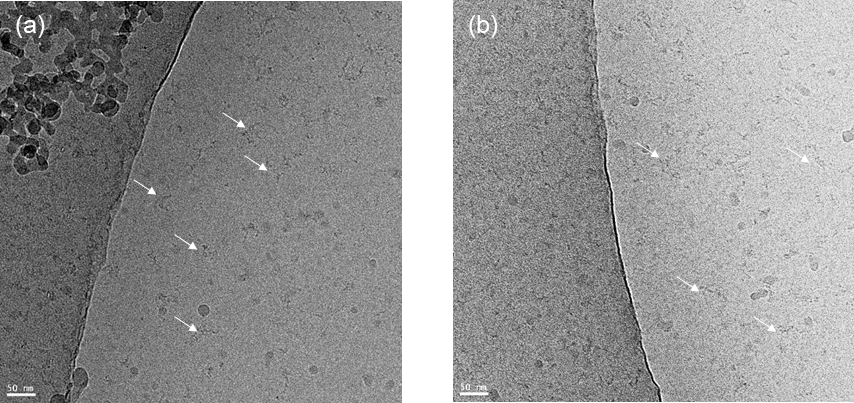


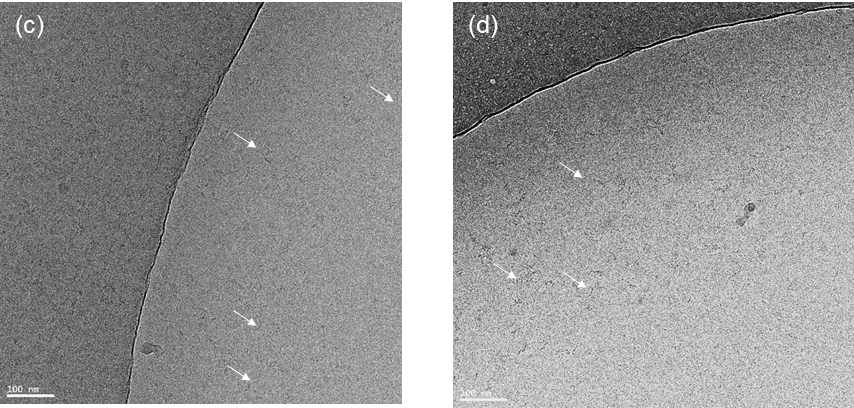


Figure **S14**. Cryo-TEM images of ***E***-**M1_st_** (1mg/ml) in water, obtained by irradiating ***Z***-**M1_st_** (1 mg/ml) with 365 nm light for 2 min at 5 ºC and maintaining the sample in the dark for 1 h in water. Worm-like micelles are identified with arrows for clearance, and not all are indicated. Similar morphology aggregates without white arrows are also micelles.


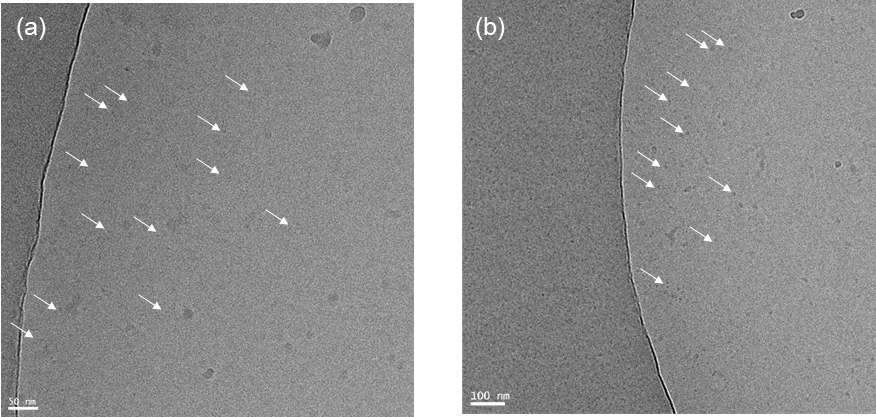


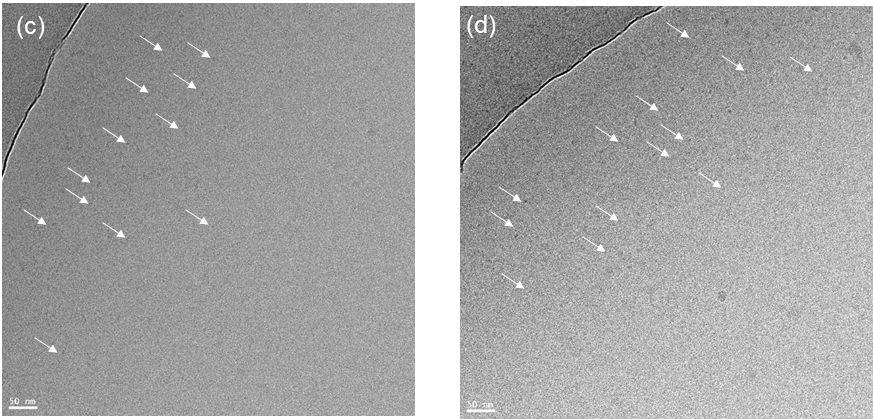


Figure **S15**. Cryo-TEM images of a PSS mixture of ***Z***-**M1_mst_** and ***E***-**M1_st_**, obtained by irradiating (***Z***-**M1_st_** (1mg/ml) with 365 nm light for 2 min at 5 ºC and maintaining the sample in the dark for 1 h in water, followed by subsequent irradiation with 365 nm light for 1 min in water, showing the micelles structures. Micelles are identified with arrows for clearance, and not all are indicated. Similar morphology aggregates without white arrows are also micelles.


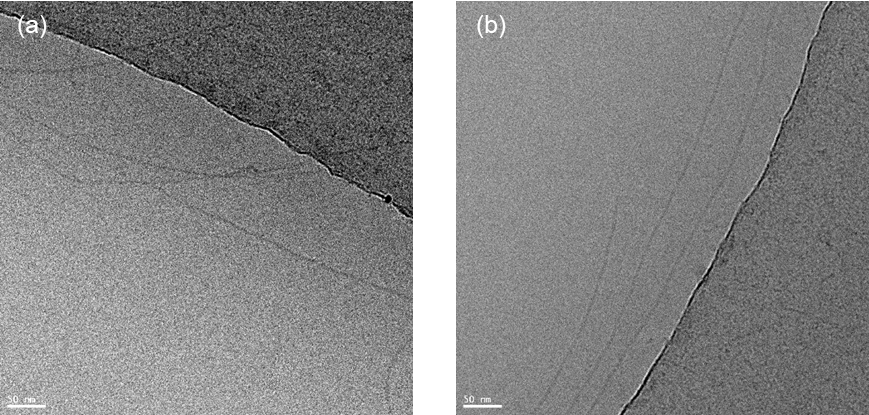


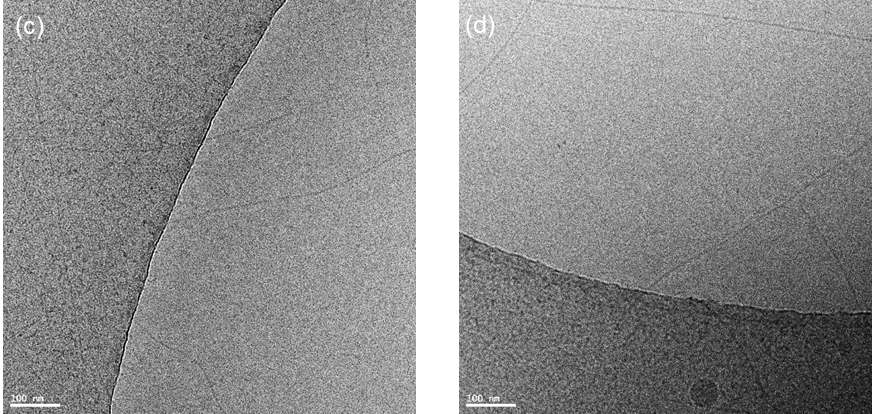


Figure **S16**. Cryo-TEM images of a mixture of ***Z***-**M1_st_** and ***E***-**M1_st_**, obtained by warming the samples of the PSS mixture shown above of ***Z***-**M1_mst_** and ***E***-**M1_st_** at 70 ºC for 5 h in water and subsequently aging overnight, showing the recovery of chiral fibers.


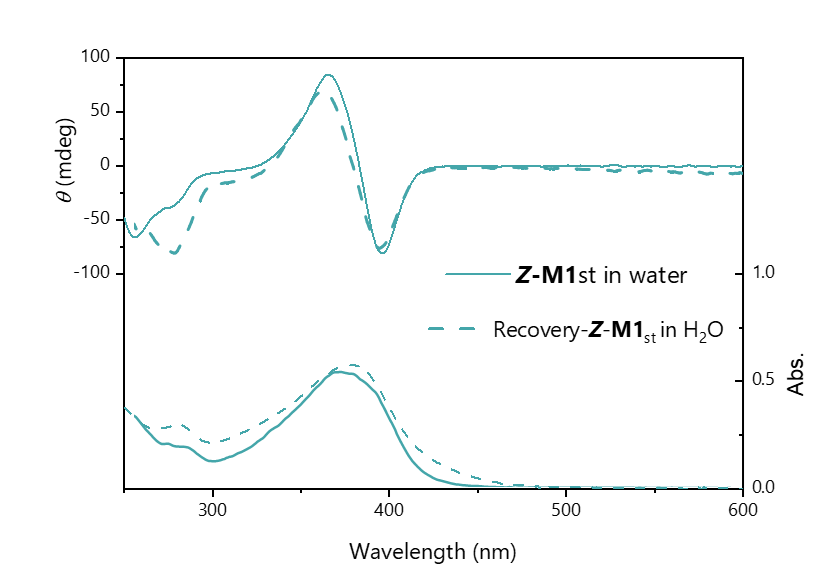


Figure **S17**. CD and UV-vis absorption spectra of the sample shown in Figure S16 (a mixture of ***Z***-**M1_st_** and ***E***-**M1_st_** in water, dotted line) compared with the initial spectra of ***Z***-**M1_st_** (solid line).

1. **Quantum yield determination**

To better investigate the function of the aldehydes for photoisomerization steps in water, we directly compared the photochemical behavior of the aldehyde functionalized motors with non-aldehyde analogues (Figure S21a).^[1]^ Our measurements show that the quantum yields are higher for the aldehyde-functionalized motors for both photoisomerization steps (for **M1**, 17.3% for ***Z*_st_**→***E*_mst_**, 47.6% for ***E*_st_**→***Z*_mst_**; for **CM**, 4.9% for ***Z*_st_**→***E*_mst_**, 20.3% for ***E*_st_**→***Z*_mst_**), indicating that the aldehyde group enhances the efficiency of the photochemical steps. Furthermore, the aldehyde-functionalized motor exhibits a comparatively low back-switching quantum yield, which contributes to the high PSS ratios and the direct recovery observed over a rotatory cycle.

**Chemical actinometry.** A modification of a standard protocol was applied for the determination of the photon flux. ^[7-8]^ An aqueous H_2_SO_4_ solution (0.05 M) containing freshly recrystallized K_3_[Fe(C_2_O_4_)_3_] (41 mM, 2 mL, 1 cm quartz cuvette) was irradiated at 20 °C for a given period in the dark with a 325/340/365 nm LED. The solution was then diluted with 1.0 mL of an aqueous H_2_SO_4_ solution (0.5 M) containing phenanthroline (1 g/) and NaOAc (122.5 g/L) and left to react for 10 min. The absorption at λ= 510 nm was measured and compared to an identically prepared non-irradiated sample. The concentration of [Fe(phenanthroline)_3_]^2+^ complex was calculated using its molar absorptivity (*ε* = 11100 M^‑1^ cm^‑1^) and considering the dilution. The quantity of Fe^2+^ ions expressed in mol was plotted versus time (expressed in seconds) and the slope, obtained by linear fitting the data points to the equation y = ax +b, equals the rate of formation of the Fe^2+^ ion at the given wavelength. This rate can be converted into the photon flux (I) by dividing it by the quantum yield of [Fe(phenanthroline)_3_]^2+^ complex (Φ^325nm^ = 1.22, Φ^365nm^ = 1.21, Φ^365nm^ = 1.20) ^[9]^ at the corresponding wavelength and by the probability of photon absorption of the Fe^3+^ complex (approximated to 1 as we were working in the total absorption regime). The obtained photon flux was determined (I^325nm^ = 1.736×10^-6^ mmol s^-1^, I^340nm^ = 6.4519×10^-6^ mmol s^-1^, I^365nm^ = 9.04737×10^-5^ mmol s^-1^).

Figure **S18**. Linear fitting of the Fe^2+^ moles generated upon irradiation of the [Fe(C_2_O_4_)_3_]^2+^ complex 325 nm at different irradiation times.

Figure **S19**. Linear fitting of the Fe^2+^ moles generated upon irradiation of the [Fe(C_2_O_4_)_3_]^2+^ complex 340 nm at different irradiation times.


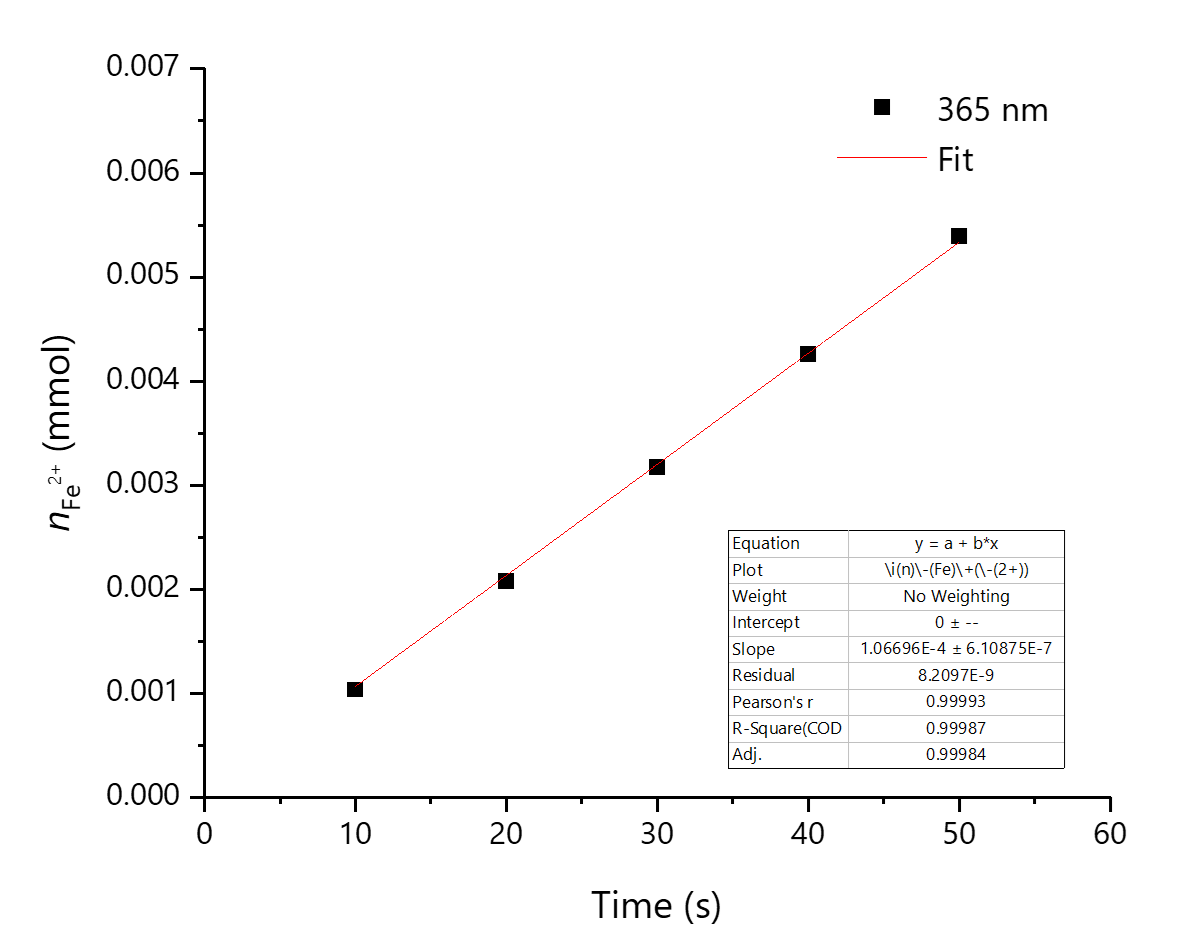


Figure **S20**. Linear fitting of the Fe^2+^ moles generated upon irradiation of the [Fe(C_2_O_4_)_3_]^2+^ complex 365 nm at different irradiation times.

**Quantum yield.** Solutions of the stable state of ***Z*-M1_st_** (~ 30 μM) in water were irradiated with an LED of a selected wavelength (365nm) to lead to the formation of the metastable state (M). The spectra were collected following the evolution of the absorption at the wavelength of irradiation. The data was subsequently fitted using COPASI 4.29^[10]^ following the same approach developed by Stranius & Börjesson.^[7]^ Equiv. 14 in the original article

$$\frac{ⅆ\left[ S \right]}{ⅆt}=-\frac{{QY}_{SM}\cdot I\cdot\beta_{S}\left( t \right)}{N_{A}\cdot V}+\frac{QY_{MS}\cdot I\cdot\beta_{M}\left( t \right)}{N_{A}\cdot V}+k_{MS^{'}}\left[ MS \right]$$

was used to determine both approximated QYs (QY*_SM_* for the formation of the metastable state form the stable and QY*_MS_* for the opposite photochemical reaction). *I* is the photon flux, previously determined with ferrioxalate actinometry, *N_A_* the Avogadro number, *V* the total volume of the irradiated solution (2 mL) and *β* the fractions of photons absorbed by either the stable or the metastable state. The decay was fitted by the ODE solver present in COPASI, using a Levenberg-Marquardt algorithm with randomized initial conditions. To obtain physically sound results, the boundaries for the QYs values were fixed between 10^-6^ and 1. The measurements were triplicated and averaged to afford the values presented in Table S2.


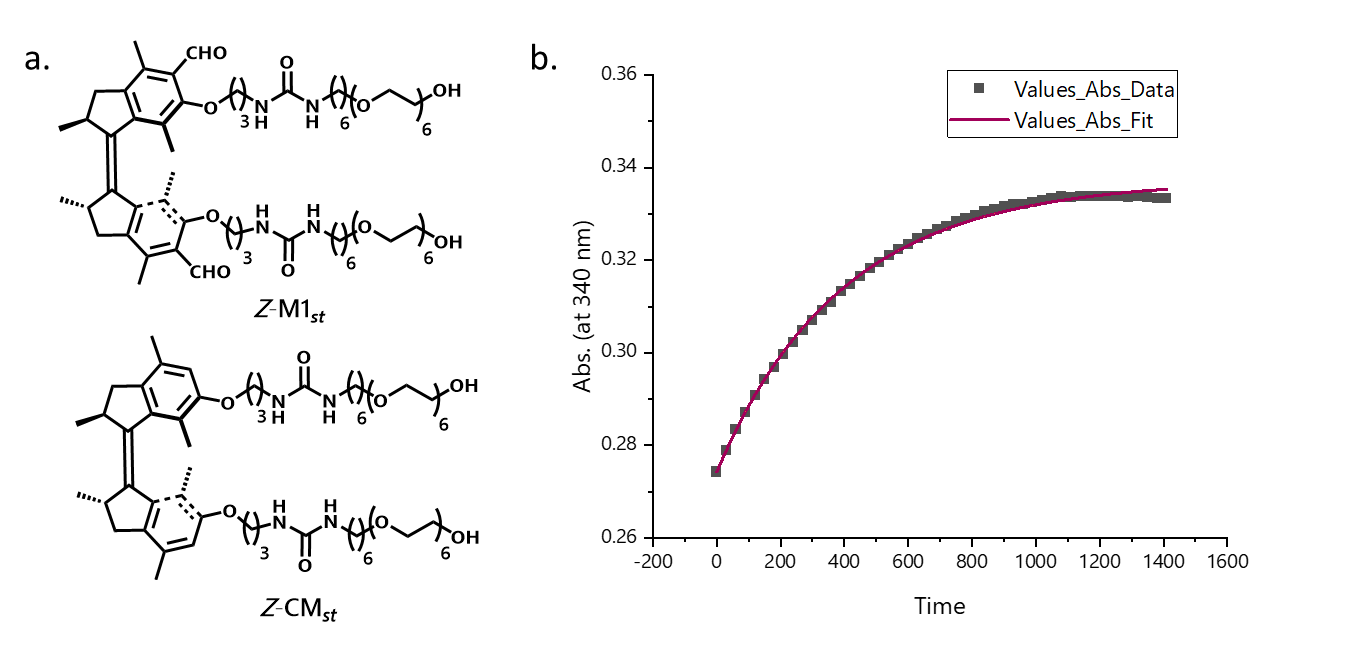


Figure **S21**. a. Chemical structure of the aldehyde-functionalized motor ***Z***-**M1*_st_*** and non-aldehyde analogues ***Z***-**CM*_st_***. b. Evolution of the absorption at 340 nm during the irradiation of ***Z***-**CM*_st_*** in water at 340 nm (The maxim absorption is around 325 nm for CMst, however, the isosbestic point is also near 325nm, therefore 340 nm is applied for investigation of the quantum yield). The red line represents the fit obtained with the ODE solver from COPASI.

Figure **S22**. Evolution of the absorption at 325 nm during the irradiation of ***E***-**CM*_st_*** in water at 325 nm. The red line represents the fit obtained with the ODE solver from COPASI.


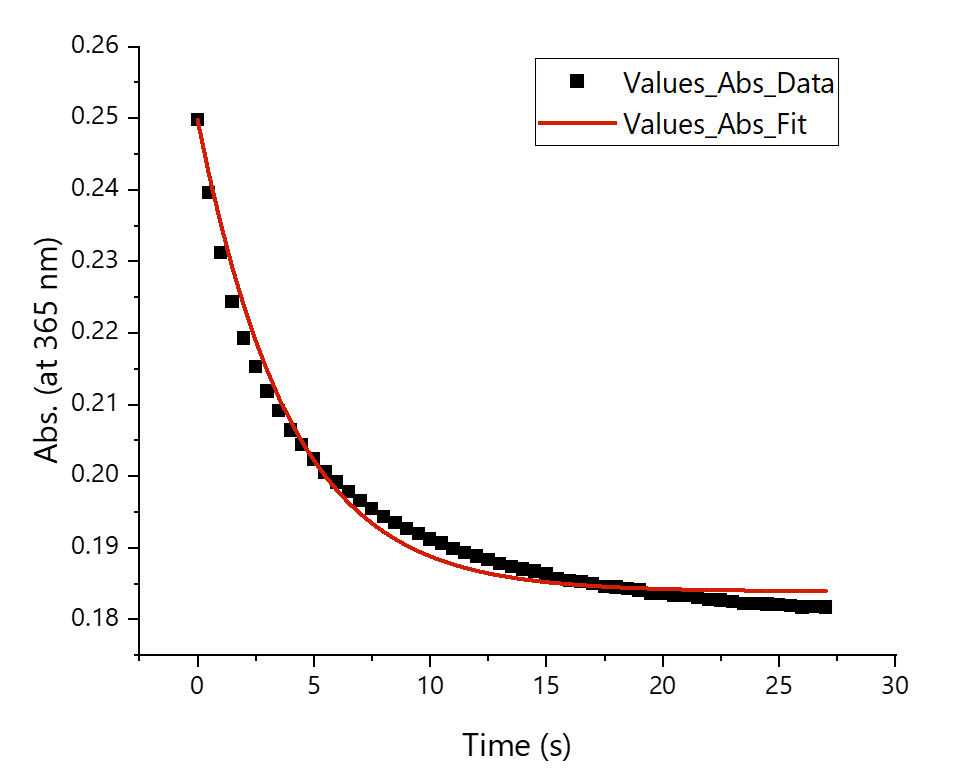


Figure **S23**. Evolution of the absorption at 365 nm during the irradiation of ***Z***-**M1_st_** in water at 365 nm. The red line represents the fit obtained with the ODE solver from COPASI.


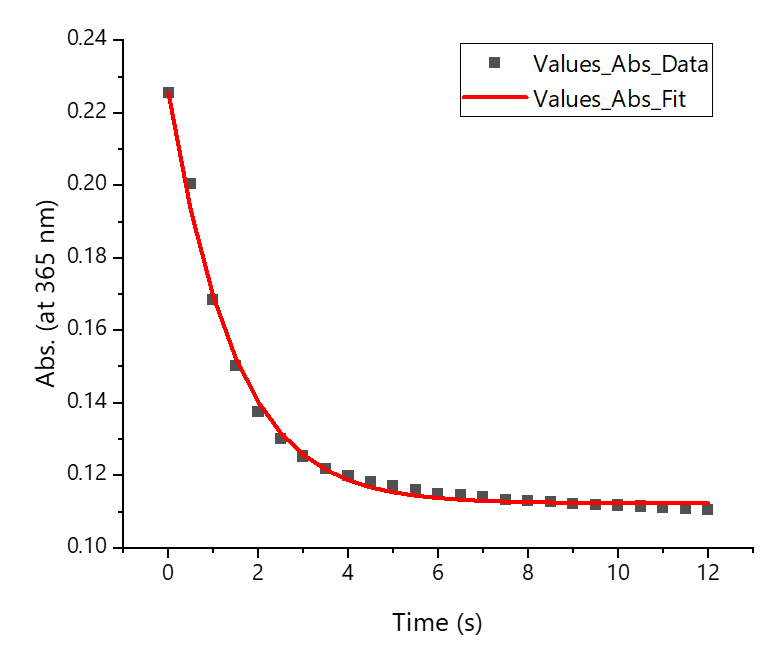


Figure **S24**. Evolution of the absorption at 365 nm during the irradiation of ***E***-**M1_st_** in water at 365 nm. The red line represents the fit obtained with the ODE solver from COPASI.

Table **S2**. Quantum yield and photo-stationary state ratio of stable and metastable diastereomers of **M1** and **CM** in water. ^[a]^

| Motor | Photo-isomerization | PSS ratio  (mst : st) | *Φ*  st → mst (%) | *Φ*  mst → st (%) |
| --- | --- | --- | --- | --- |
| **M1** | ***Z*_st_ → *E*_mst_** | 99:1 | 17.3±0.3 | 2.1±0.1 |
| **CM** | ***Z*_st_ → *E*_mst_** | 99:1^[b]^ | 4.9±0.5 | 0.2±0.1 |
| **M1** | ***E*_st_ → *Z*_mst_**^[c]^ | 87:13 | 47.6±0.8 | 2.5±0.4 |
| **CM** | ***E*_st_ → *Z*_mst_** | 68:32^[b]^ | 20.7±1.6 | 9.2±0.9 |

[a] The molar absorption coefficients for the stable states were obtained by UV-Vis measurements. The ones for the metastable-states were obtained by extracting the values from the UV-Vis spectrum at the PSS, knowing the concentration of stable and metastable species as obtained by ^1^H-NMR. [b] PSS ration extracted from the previous reports.^[1]^ [c] ***E*_st_** was generated by irradiation of ***Z*_st_** at 365nm and waiting for 2h at room temperature in the dark.

1. **Model reaction of MOMe with NaHSO_3_**

Figure **S25**. The reversible nucleophilic addition between **MOMe** and **MOMe-NaHSO_3_**.

In order to investigate the reversible reaction between aldehyde and sodium bisulfite in water, we have taken the small molecule MOMe as a demonstration for evidencing the reactions as well as the in-situ analysis of the transformation of aldehyde group to an α-hydroxy sulfonate products via NMR spectroscopy (Figure S18). ^[11-12]^ Before addition, the products displayed a bright yellow color in the NMR tube in DMSO-*d*6 (0.5 mM, Figure S19a). On addition of NaHSO_3_ (1M in D_2_O, 0.05 mL), the characterized absorption for aldehyde (~ 10.5 ppm) disappeared with the generation of absorptions which could be attributed to the signal of hemi-acetal (~ 5.6-5.7 ppm, Figure S19b, S20). Once the system was treated with Na_2_CO_3_ (1M in D_2_O, 0.1 mL), the precipitation of yellowish solid was observed immediately, which could be identified by the ^1^H-NMR analysis as the recovery of **MOMe** (Figure S19c).


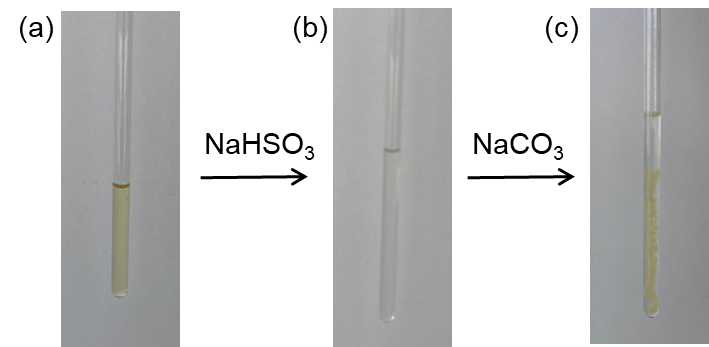


Figure **S26**. Photograph of the color changes during the addition processes in the NMR tube.

Figure **S27**. ^1^H-NMR spectra of the **MOMe-NaHSO_3_** adduct (D_2_O:DMSO = 9:1).

1. **UV-vis spectroscopic study of the reaction of molecular motors M1 with NaHSO_3_**


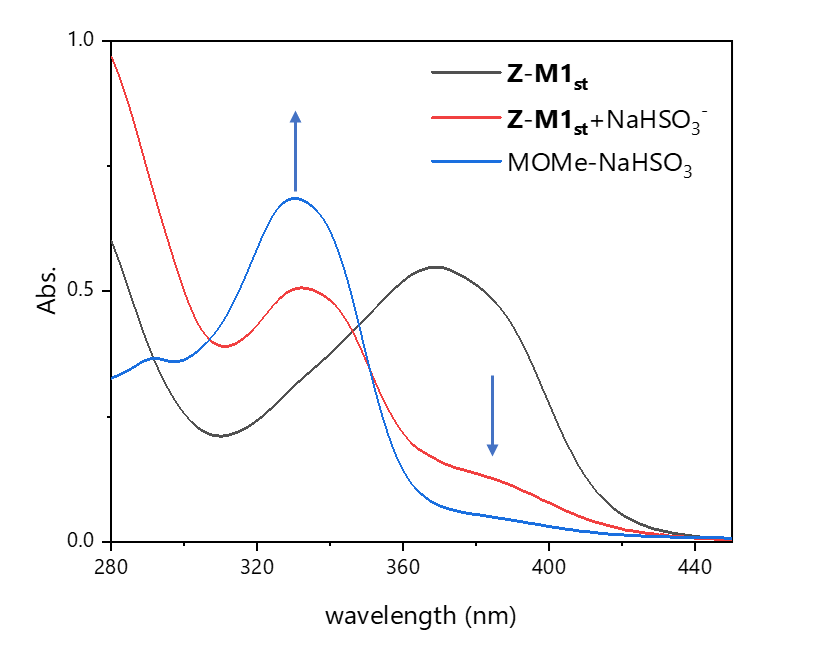


Figure **S28**. UV-vis absorption spectra of the MOMe-NaHSO3 (40 µM, water), ***Z***-**M1st** (35 μM, water) and ***Z***-**M1st** upon the addition of NaHSO_3_ (2000 eq.).

Different from the initial aldehyde motor, which exhibited a maximum absorption at 365 nm, the bisulfite addition products (**MOMe-NaHSO_3_**) displayed a blue-shift maximum absorption at 330 nm with a shoulder at 380 nm. This distinct property offered us a possibility for in-situ analysis of the aldehyde-sulfonate transformation reactions as well as the behavior of the supramolecular polymer ***Z***-**M1st** in aqueous media (Figure S21). Based on these observations, titration experiments were conducted with different equivalents of NaHSO_3_ added to the aqueous solution of ***Z***-**M1st** and performed the kinetic analysis in the UV-Vis spectral changes.

General Method for ***Z***-**M1st**: To the 1cm quartz cell with ***Z***-**M1st** (20 μM, 2.5 mL) and 1 mm stirring bar was added NaHSO_3_ (1M, 5 to 75 μL in water), and the reaction was monitored with the UV-Vis spectra every 120 s.


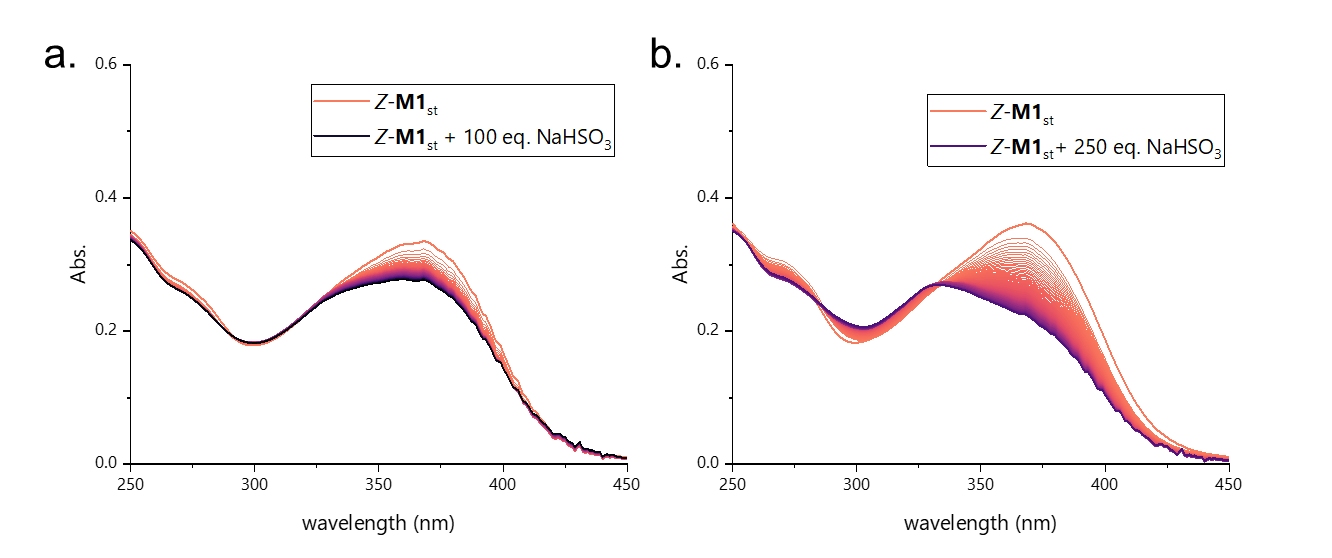


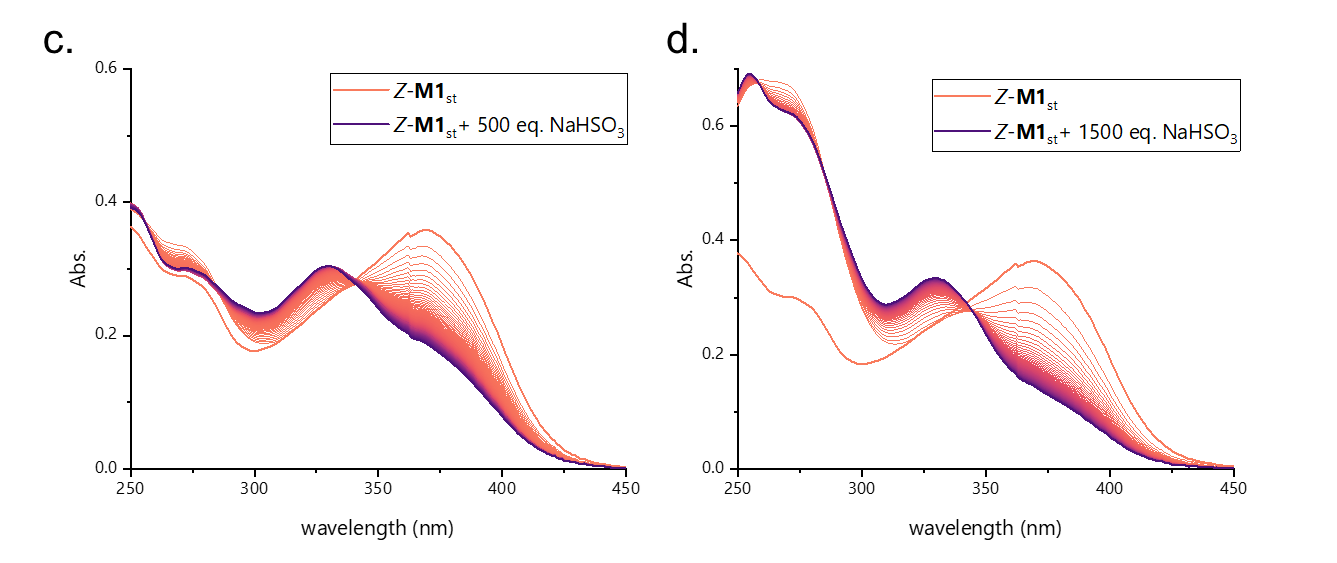


Figure **S29**. UV-vis absorption spectra of the ***Z***-**M1st** (20 μM in water) upon the addition of NaHSO_3_ (a. 100 eq., b. 200eq., c. 500 eq. and d. 1500 eq.).


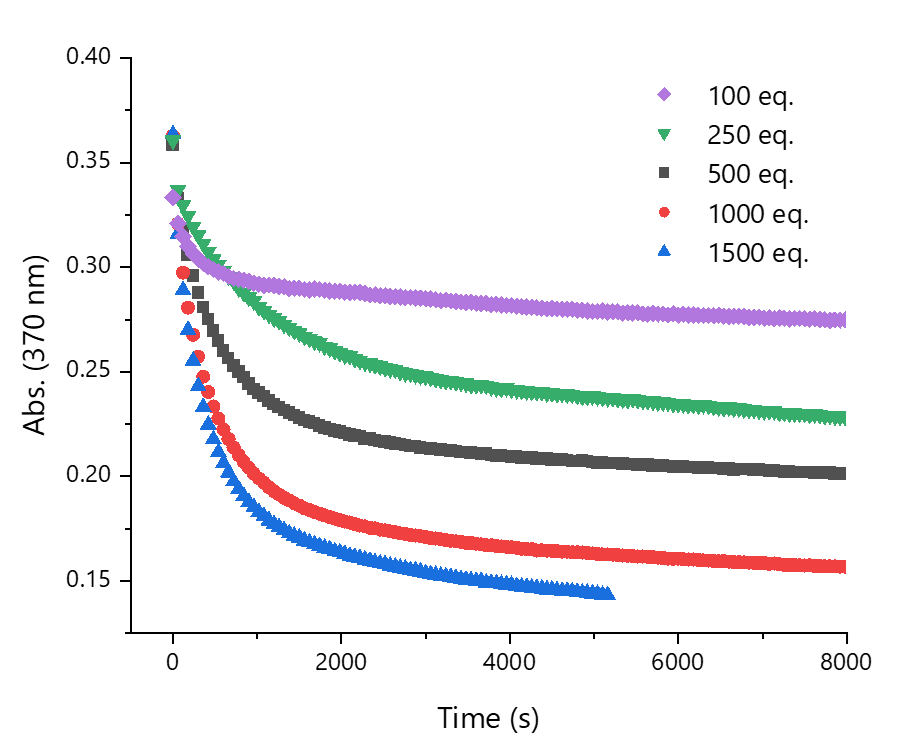


Figure **S30**. Kinetic analysis of the ***Z***-**M1st** (20 μM in water) upon the reaction with NaHSO_3_, monitored by the UV/Vis absorption at 370nm.

Given the pH sensitivity of the nucleophilic reaction, buffer systems with varying pH values were employed alongside pure water to evaluate their influence on reaction kinetics. Upon treatment with 1000 equivalents of sodium bisulfite (NaHSO₃), the reaction performed in citrate buffer (0.1 M, pH 5.3) exhibited a significantly higher rate and more efficient conversion compared to those in pure water and phosphate-buffered saline (PBS, 0.1 M, pH 7.4), both of which displayed reduced reaction rates and lower overall conversion. This trend corresponds with increasing pH, indicating an inverse correlation between reaction efficiency and buffer pH under the tested conditions, likely attributable to the underlying mechanistic characteristics of nucleophilic substitution processes.


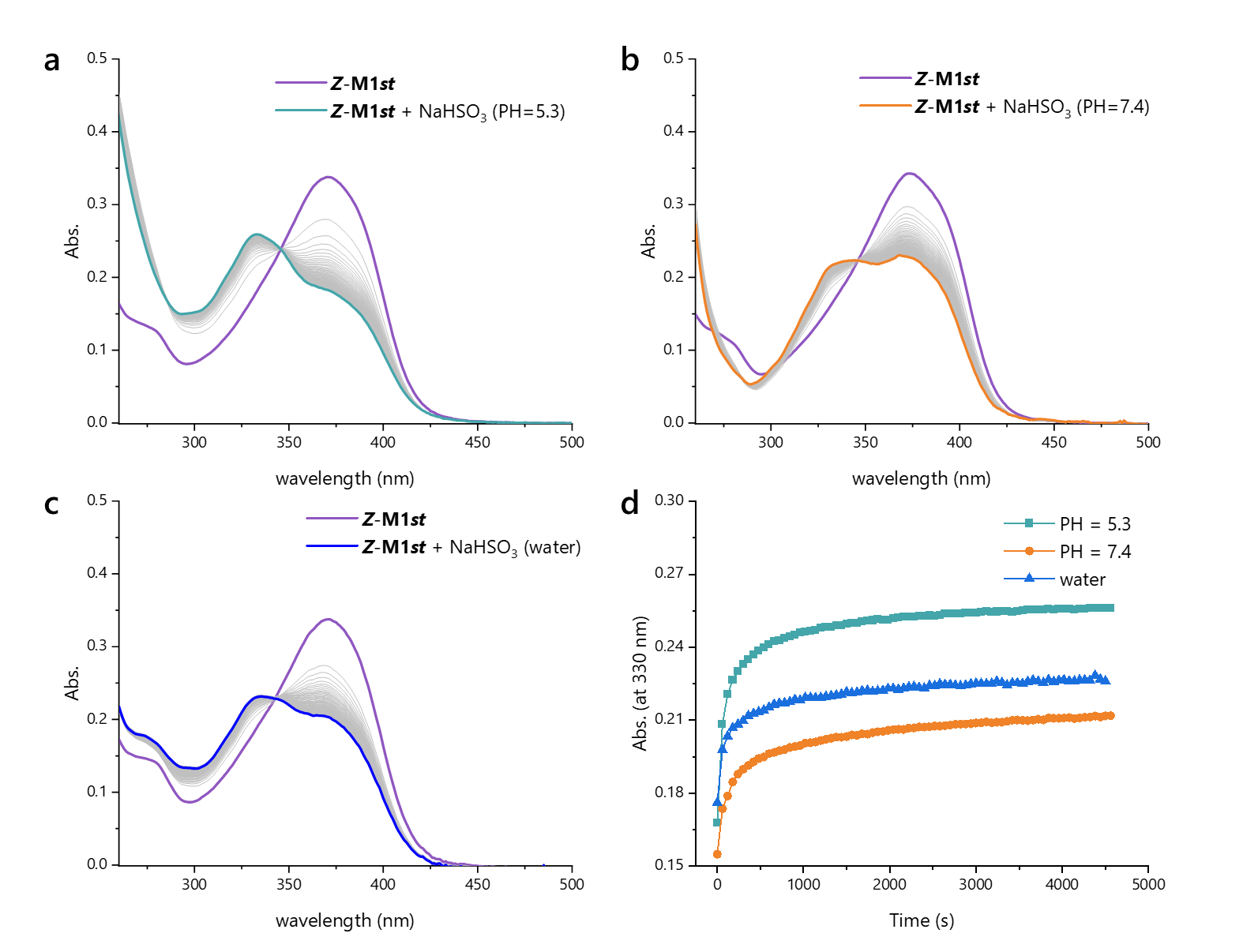


Figure **S31**. UV-vis absorption spectra of the ***Z***-**M1st** (20 μM, 2 ml) upon the reaction with NaHSO_3_ (1000 eq.) in different buffer (a. citrate buffer (0.1 M, pH 5.3), b. PBS buffer (0.1 M, pH 7.4), c. water). (d) Kinetic analysis monitored by the UV/Vis absorption at 330nm, the reaction was monitored with the UV-Vis spectra every 60 s

1. **UV-Vis** **spectroscopic study of the recovery of molecular motors M1 with Na_2_CO_3_**

For the recovery processes, 20 μL of Na_2_CO_3_ solution (2M in aq. solution) was directly added into the reaction mixture, which consumed the sulfonate products and rapidly regenerated the original ***Z***-**M1st** molecules.


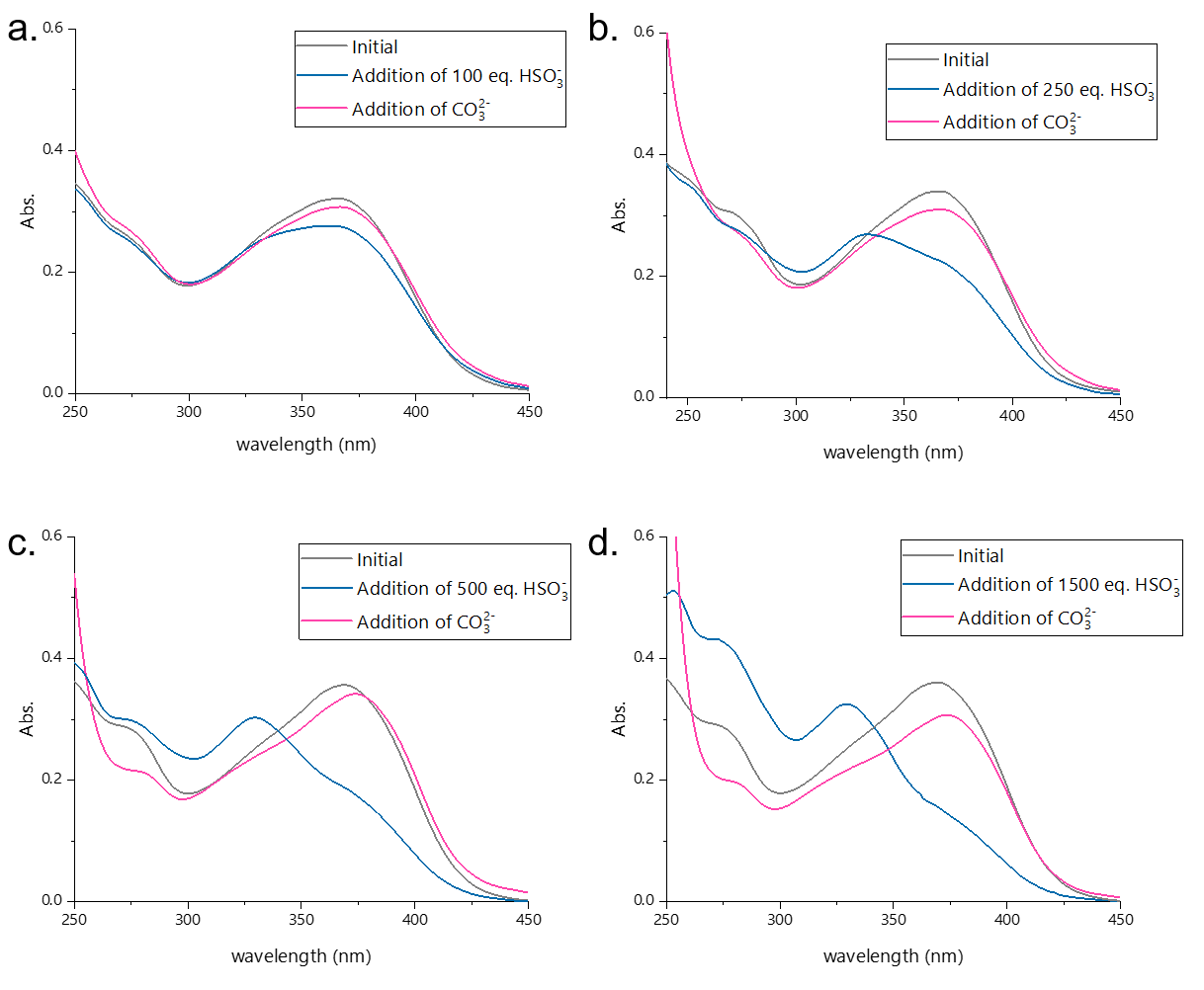


Figure **S32**. UV-vis absorption spectra of the initial ***Z***-**M1st** (20 μM in aq. solution), the corresponding sulfonate products of ***Z***-**M1st** upon the addition of NaHSO_3_ (a. 100 eq., b. 200 eq., c. 500 eq. and d. 1500 eq.) and the recovery products of ***Z***-**M1st**.


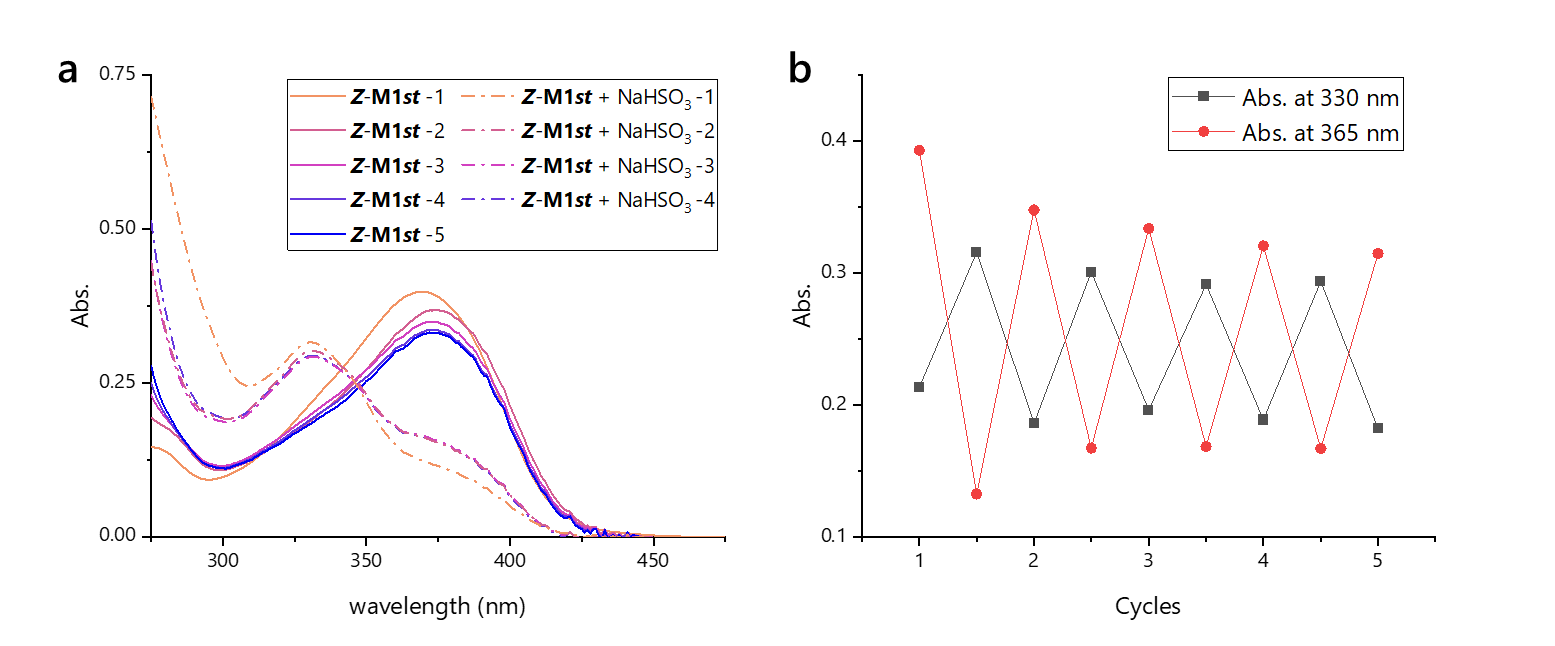


Figure **S33**. Fatigue study of ***Z***-**M1st** (20 μM in aq. solution) with bisulfite/base cycles. (a) UV-vis absorption spectra during the cycles, (b) absorbance signals after sequential bisulfite and base treatment of ***Z***-**M1st** solution in water at 330 and 365 nm.

1. **CD spectroscopic study of the reaction of molecular motors M1 with NaHSO_3_**

To further investigate the behavior of the supramolecular polymers, titration experiments were monitored by circular dichroism (CD) spectroscopy to track changes in both molecular chirality and supramolecular chiral transformations during the self-assembly process. The same parameters used in the UV-Vis measurements were applied to ensure better comparability.


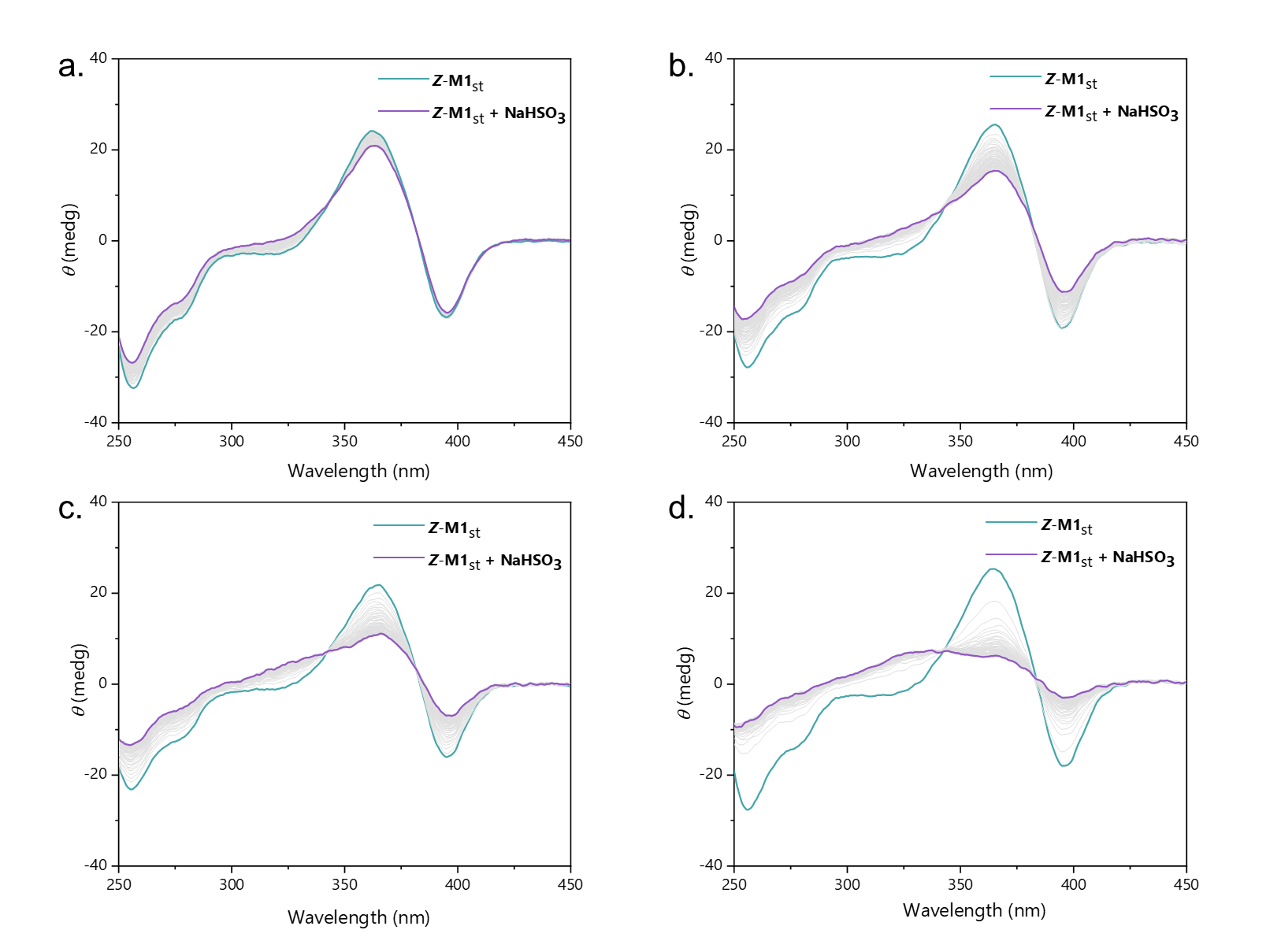


Figure **S34**. CD spectra of the ***Z***-**M1st** (20 μM in aq. solution) upon the addition of NaHSO_3_ (a. 100 eq., b. 200 eq., c. 500 eq. and d. 1500 eq.).


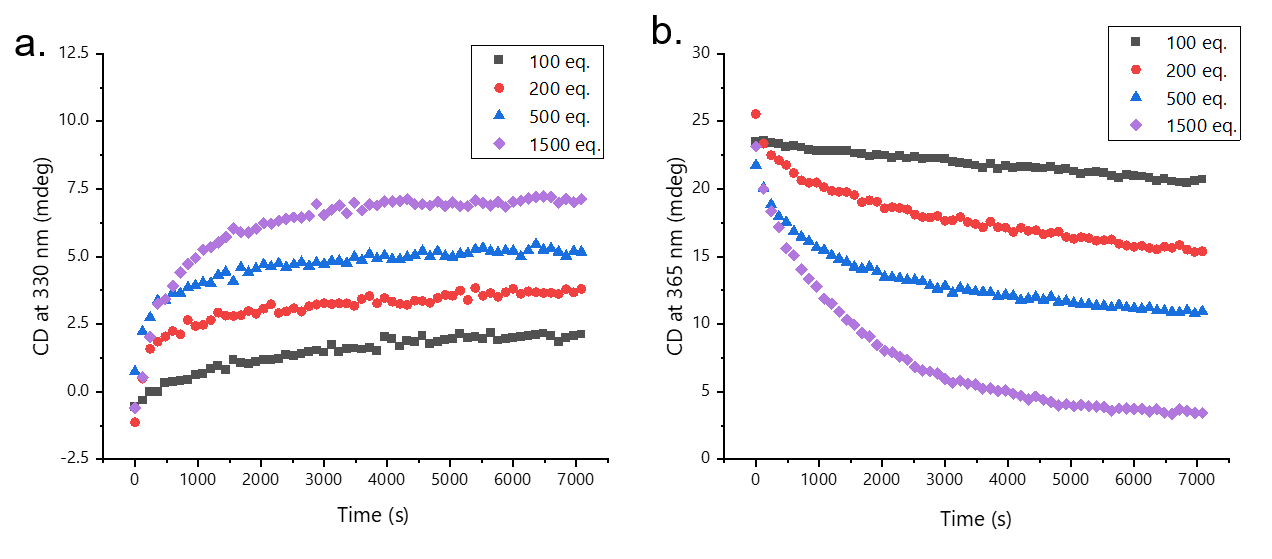


Figure **S35**. Kinetic analysis of the transformation of ***Z***-**M1st** (20 μM) upon reaction with NaHSO_3_ in H_2_O (monitored by CD spectroscopy at (a) 330nm and (b) 365 nm).

1. **CD spectroscopic study of the recovery of molecular motors M1 with Na_2_CO_3_**


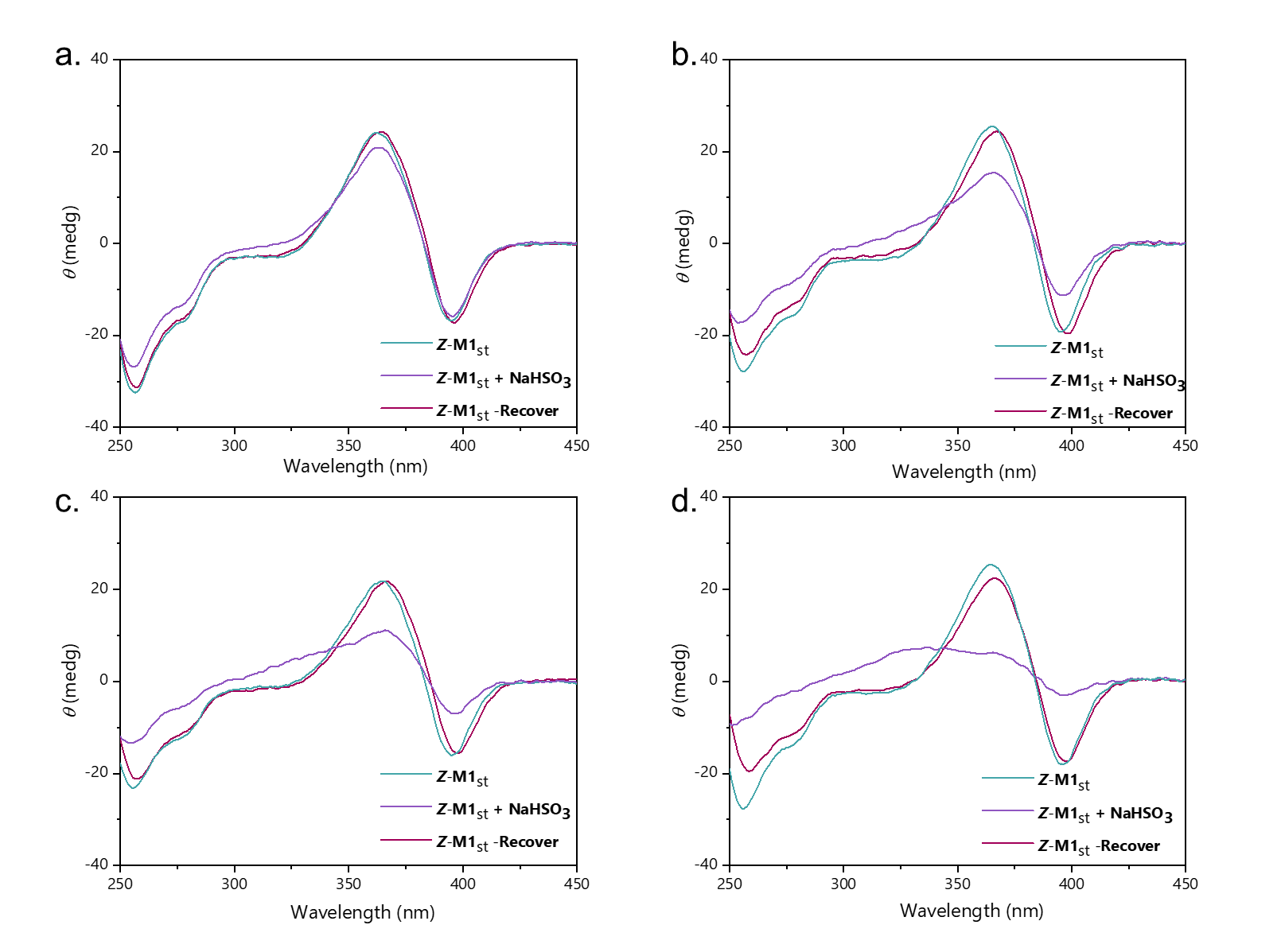


Figure **S36**. CD spectra of the initial ***Z***-**M1st** (20 μM in aq. solution), the corresponding sulfonate products of ***Z***-**M1st** upon the addition of NaHSO_3_ (a. 100 eq., b. 200 eq., c. 500 eq. and d. 1500 eq.) and the recovery products of ***Z***-**M1st**.

1. **Rotary cycle of *Z-*M1_st_+NaHSO_3_ upon irradiation**

To further investigate the photoisomerization process of ***Z***-**M1*_st_***+NaHSO_3_, UV/vis and CD spectroscopy were applied to track changes during the irradiation process. Sample preparation: To a solution of ***Z***-**M1_st_** in water was added 1000 eq. of the NaHSO_3_ (0.1M in water). After stirring for 6 h, the reaction reached equilibrium with the formation of hydroxy sulfonate motors, confirmed by the UV-vis spectra.

For the **hydroxy sulfonate motors**, when applied with 340 nm LED, the characteristic absorption band at 320-340 nm decreased and an absorption band at 350-400 nm appeared with an isosbestic point at 339 nm, which might indicate the selective photochemical interconversion of ***Z***-**M1*_st_***+NaHSO_3_ to the photostationary state, named PSS_340nm_-***E***-**M1*_mst_***+NaHSO_3_ (Figure S37a and Figure S38a, step1). In the CD spectra, the positive signal of ***Z***-**M1*_st_***+NaHSO_3_ at 300-350 nm decreased with the generation of a negative band at 350-400 nm upon irradiation, which also reflects the formation of PSS_340nm_-***E***-**M1*_mst_***+NaHSO_3_ with opposite chirality (Figure S38b, green line). Subsequently kept the PSS_340nm_-***E***-**M1*_mst_***+NaHSO_3_ resulted in the generation of THI-***E***-**M1*_st_***+NaHSO_3_ with an isosbestic point at 333nm in the UV/vis spectra and the inversion of the CD signals from the negative band at 360nm to the positive peak form 300 to 350 nm (Figure S37b and S38b, blue line).

For the secondary photoisomerization step, only subtle changes were observed in the UV/vis spectra after irradiation, showing a decrease at 300-340 nm and an increase in the signal at 370nm, with an isosbestic point at 345 nm. In the CD spectra, a new negative band generated at 370nm, suggesting the photoisomerization step involving inverted chirality (Figure S37c). However, the persistent positive signal belong to the THI-***E***-**M1*_st_***+NaHSO_3_ indicated that the ***E*** to ***Z*** photoisomerization is no efficiently happening upon irradiation (Figure S38b, orange line). The reason for this poor photoisomerization process could be that the conversion of the aldehyde to the hydroxy sulfonate group removes the strong electron-withdrawing effect, thus resulting in the photoisomerization behavior resembling that of the non-aldehyde motors with less efficient performance under irradiation. Furthermore, in step4, which typically requires a high temperature, we attempted to warm the aqueous solution to 60 °C, but recovery of the initial state could not be achieved. During the warming process, continuous babbling was observed and we believed it could be attributed the decomposition of the instability of NaHSO_3_ at elevated temperature. Consequently, the photoisomerization is significantly influenced as hydroxy sulfonate motor in aqueous media.


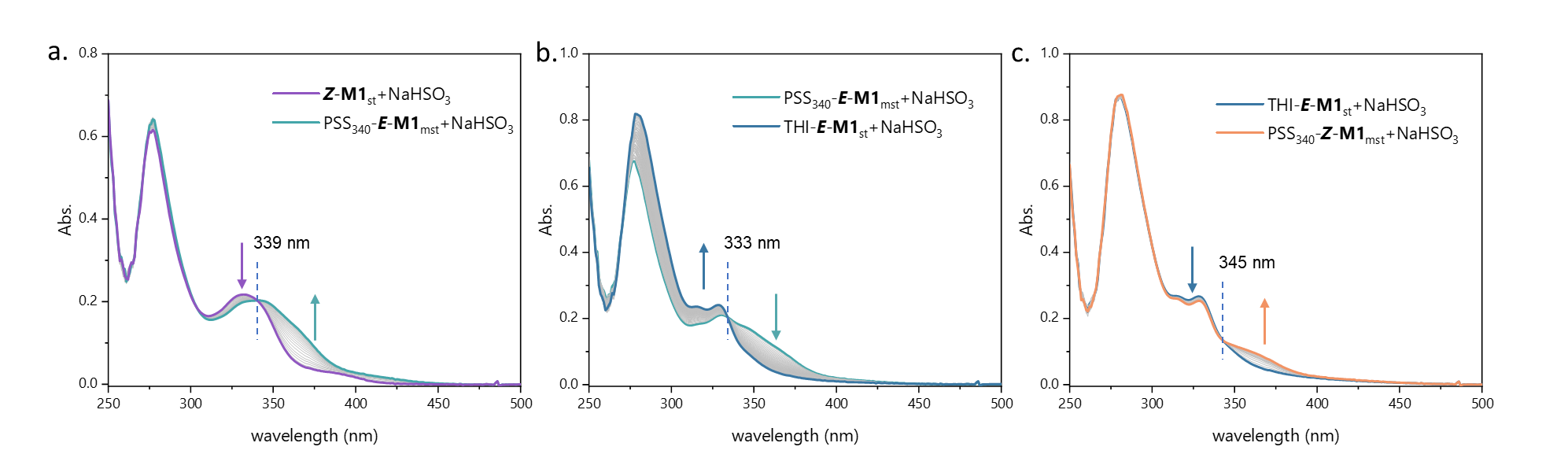


Figure **S37**. Changes in the UV-vis absorption spectra of ***Z***-**M1_st_**+NaHSO_3_ (15 μM, mixed with 1000 eq. NaHSO_3_ for 6h before measurement) in water. (a) upon 340 nm light irradiation for 5 min at 5 ºC to form PSS_340_-***E***-**M1_mst_**+NaHSO_3_, (b) maintaining the solution in the dark at 5 ºC for 2 h to reach THI-***E***-**M1_st_**+NaHSO_3_, (c) subsequent irradiating with 365 nm light at 5 ºC for 10 min to yield PSS_340_-***Z***-**M1_mst_**+NaHSO_3_.


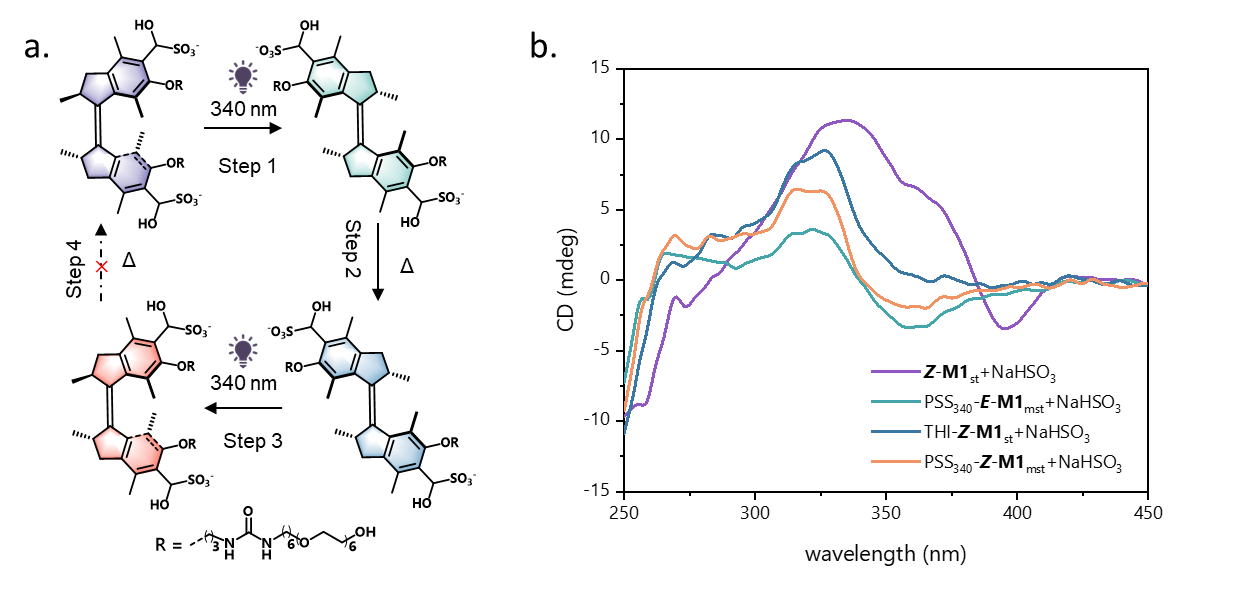


Figure **S38**. (a). Structural changes during the four-step unidirectional isomerization of the hydroxy sulfonate motors. (b). Changes in the CD spectra of ***Z***-**M1_st_**+NaHSO_3_ (15 μM in water, mixed with 1000 eq. NaHSO_3_ for 6h before measurement) during photochemcial reaction and thermal helix inversion process.

1. **Cryo-TEM analysis of *Z*-M2_st_ and recovery**


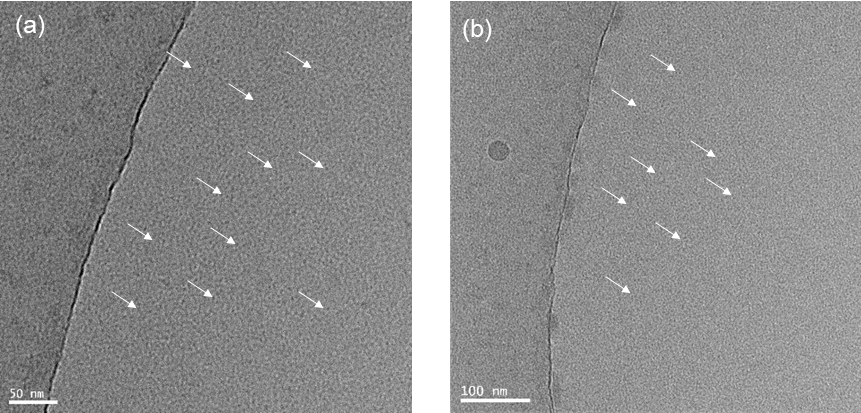


Figure **S39**. Cryo-TEM images of ***Z***-**M2_st_**, obtained after adding the NaHSO_3_ (1 M in water) to ***Z***-**M1_st_** (1 mg/ml) and aging for 1 d in the dark, showing the disappear of the chiral fibers formed by ***Z***-**M1_st_** and the generation of small micelles. Micelles are pointed out with arrows for clearance, and not all are indicated. Morphology aggregates not indicated with white arrows are also micelles.


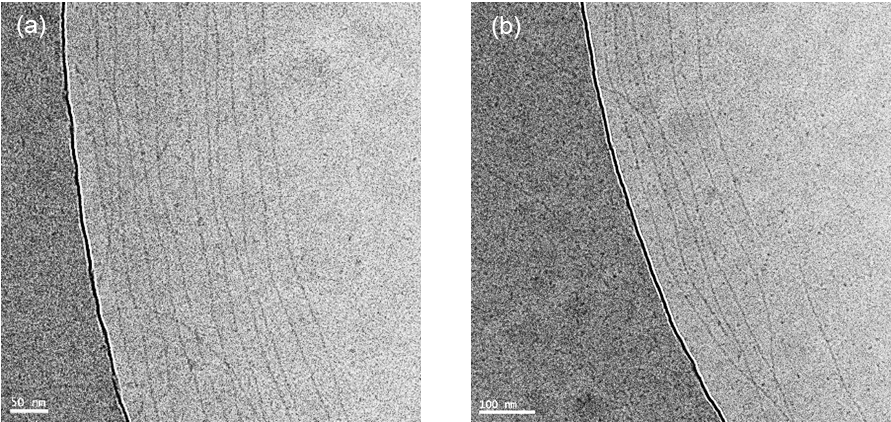


Figure **S40**. Cryo-TEM images after the recovery process from ***Z***-**M2_st_** to ***Z***-**M1_st_**, obtained by adding Na_2_CO_3_ (2 M in water) to ***Z***-**M2_st_** (generated above, showing micelles structures) and after aging for 1 d in the dark, showing the recovery of the chiral fibers formed by ***Z***-**M1_st_**.

1. **Model reaction of MOMe with NH_2_OH**

Figure **S41**. Scheme of the reaction between **MOMe** and NH_2_OH

We have also applied the model reaction between small aldehyde motor **MOMe** and water-soluble hydroxylamine to investigate the process of imine formation. ^[13-14]^

General Method: To a suspension of **MOMe** (40.4 mg, 0.1 mmol) in ethanol (5 mL) at was slowly added an aqueous solution of NH_2_OH (593.4 µL, 50 wt%, 10 mmol). The mixture was stirring at room temperature for 1 day and then quenched with water. The aqueous phase was extracted with EtOAc (3*25 mL) and the combined organic layer was washed with brine, dried over Na_2_SO_4_ and concentrated under vacuo. The crude product was purified by column chromatography (SiO_2_, pentane:EtOAc = 20:1 to 10:1) to afford compound **MOMe-NH_2_OH** (24.6 mg, 0.06 mmol, 60%) as a yellow solid. ^1^H NMR (400 MHz, CDCl_3_) δ 8.45 (s, 2H), 8.10 (s, 2H), 7.53 (s, 2H), 3.72 (s, 6H), 3.37 (p, *J* = 6.7 Hz, 2H), 3.19 (dd, *J* = 14.7, 6.3 Hz, 2H), 2.48 (d, *J* = 14.7 Hz, 2H), 1.51 (s, 6H), 1.08 (d, *J* = 6.7 Hz, 6H). ^13^C NMR (101 MHz, CDCl_3_) δ 156.17, 147.14, 144.93, 142.18, 141.93, 129.12, 123.42, 119.60, 61.29, 42.73, 39.88, 19.99, 14.56.

From the ^1^H-NMR analysis, the characterized aldehyde absorption disappeared with the formation of two single absorption at 8.45 and 8.10 ppm, which could be identified as the oxime analogues.

1. **UV-vis spectroscopic study of the reaction of molecular motors M1 with NH_2_OH**

General Method for the reaction between ***Z***-**M1st** and NH_2_OH: To the 1cm quartz cell with ***Z***-**M1st** (20 μM, 2.5 mL in aq. Solution with 1 mm stirring bar) was added NH_2_OH (1M, 50 to 150 μL), and the reaction was monitored using the UV-Vis spectroscopy every 120 s over a period of 5 h.


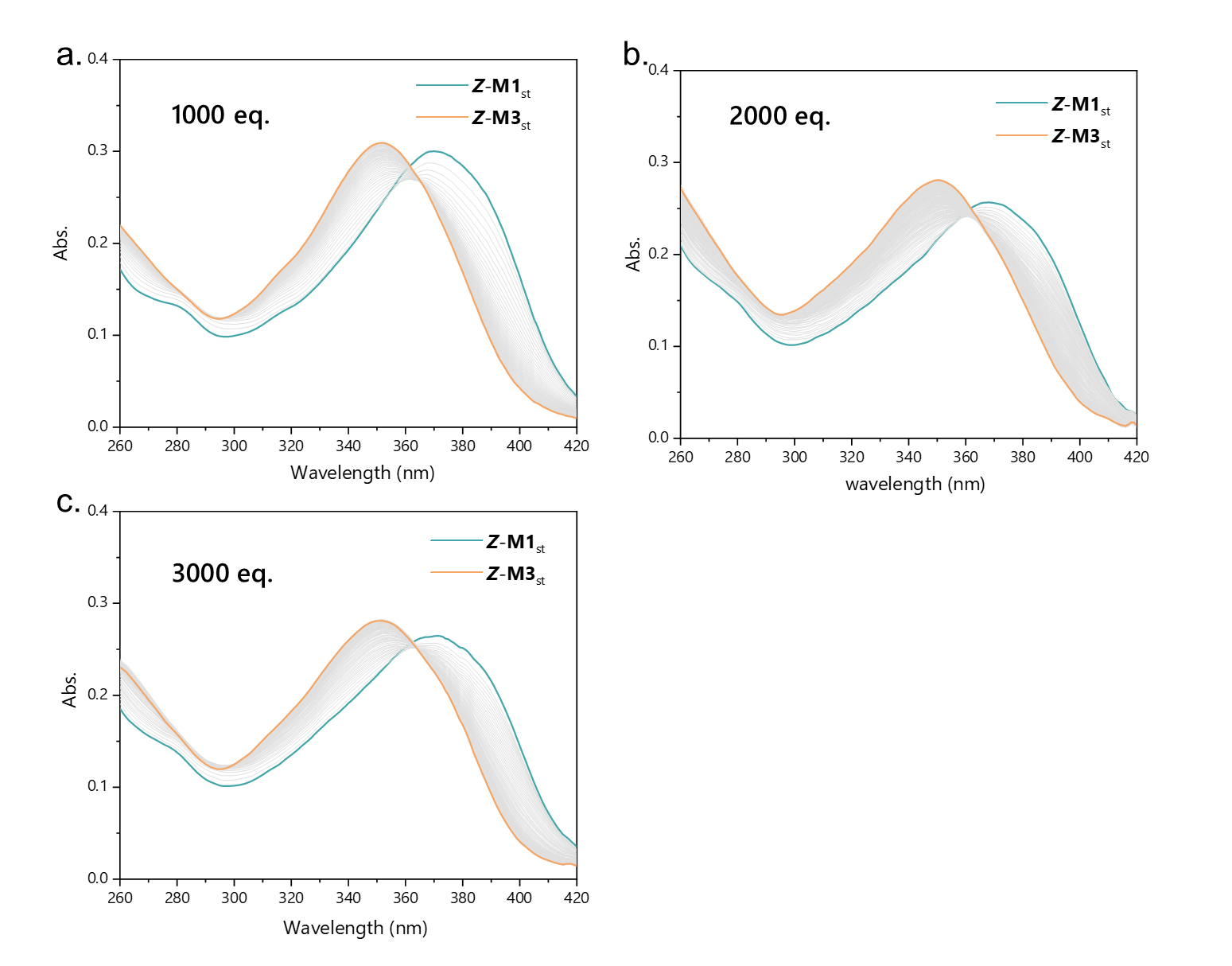


Figure **S42**. UV-vis absorption spectra of the ***Z***-**M1st** (20 μM in aq. solution) upon the addition of NH_2_OH (a. 1000 eq., b. 2000 eq., and c. 3000 eq.).

1. **CD spectroscopic study of the reaction of molecular motors M1 with NH_2_OH**


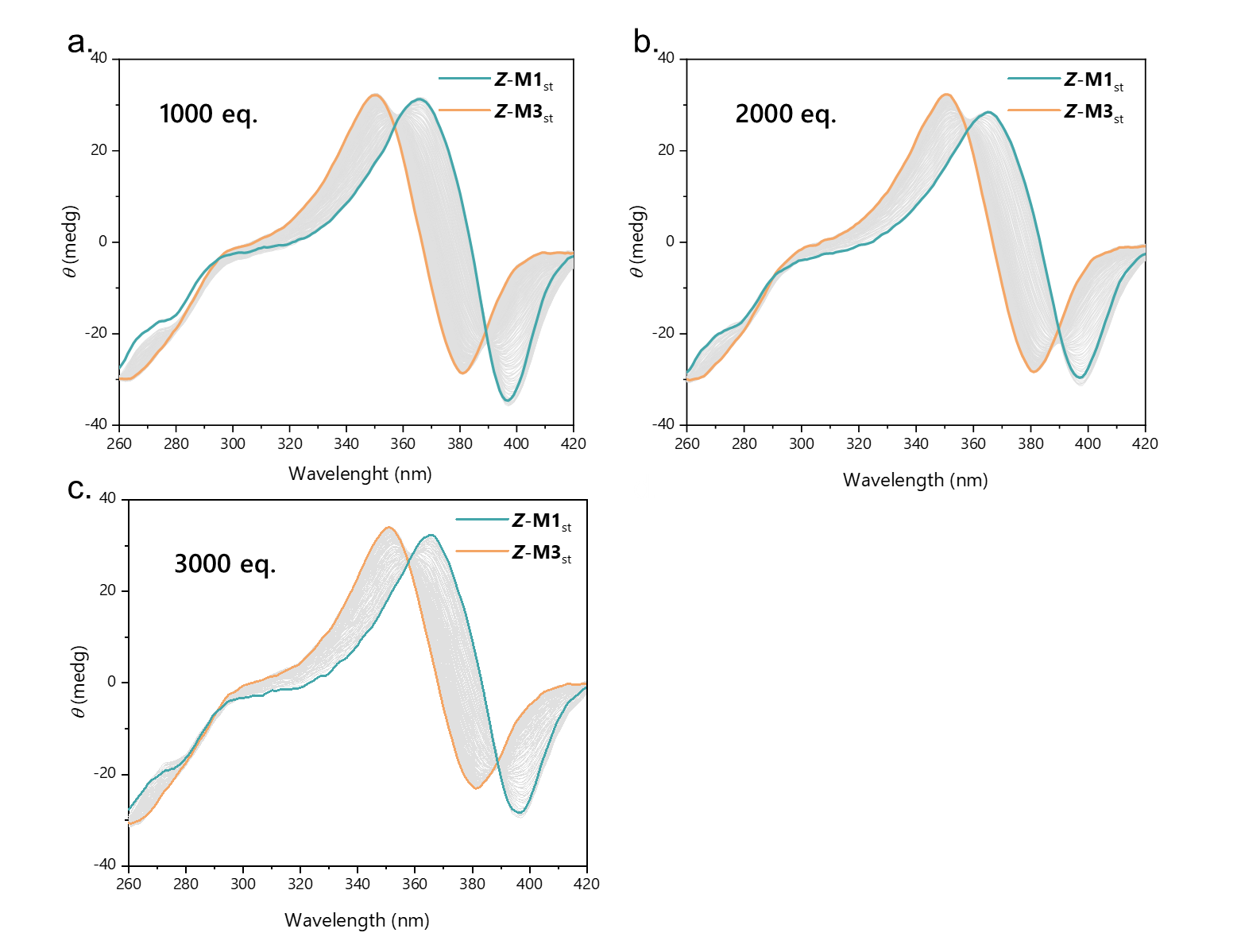


Figure **S43**. CD spectra of the ***Z***-**M1st** (20 μM in aq. solution) upon the addition of NH_2_OH (a. 1000 eq., b. 2000eq., and c. 3000 eq.).

1. **Fourier transform infrared spectroscopic study of *Z*-M1st and *Z*-M3st**

Supramolecular polymers of ***Z***-**M1st** and ***Z***-**M3st** were measured on a PerkinElmer Spectrum 400. Samples were characterized after freeze-drying from water.


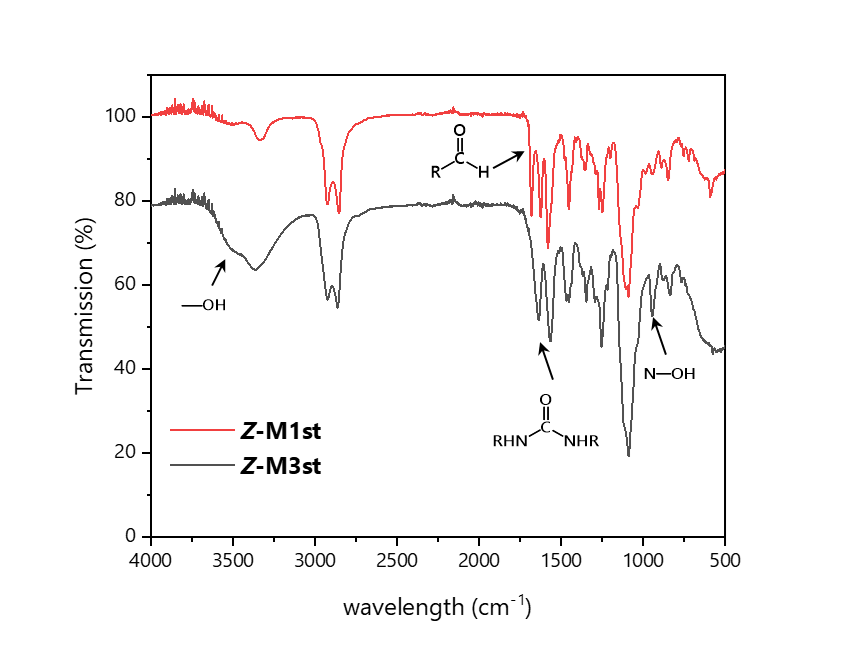


Figure **S44**. FTIR spectra of ***Z***-**M1st** and ***Z***-**M3st**.

1. **Rotary cycle of *Z-*M3_st_ upon irradiation**

To further investigate the photoisomerization process of oxime motors ***Z*-M3_st_**, UV/vis, CD spectroscopy and Cryo-TEM were applied to track changes during the irradiation process. Sample preparation: To a solution of ***Z***-**M1_st_** in water was added 1000 eq. of the hydroxylamine (0.1M in water), after stirring for 6 h, the conversion form aldehyde to oxime is finished followed by the UV/vis and CD spectra.

For the **oxime motors**, upon irradiation with 340 nm light for 5 min, the characteristic absorption band of ***Z***-**M3*_st_*** at 355 nm decreased with the increase of an absorption band at 375 nm, showing a clear isosbestic point at 360 nm and generation of PSS_340_-***E***-**M3*_mst_*** (Figure S45a). Additionally, the negative Cotton effect attributed to the supramolecular polymer structures disappeared while a negative signal appeared at 350-400 nm in the CD spectra, in accordance with the selective photochemical interconversion of ***Z***-**M3*_st_*** to PSS_340nm_-***E***-**M3*_mst_*** (Figure S46, green line). Subsequently keeping the sample in the dark for 2 h resulted in the THI process and the formation of THI-***E***-**M3*_st_***, displaying the inverted CD signal from a negative band to a positive band at 300-370 nm as well as the blue-shift of the maximum absorption band to 340 nm (Figure S45b and Figure S46b, blue line). After irradiation and subsequently warming to reach THI-***E***-**M3*_st_***, the fiber formed by ***Z***-**M3*_st_*** disappeared and large aggregated particles were observed in the cryo-TEM images (Figure S50b).

Next, upon irradiation at 340 nm for 10 min, the absorption band and positive CD signal at 300–370 nm of the THI-***E***-**M1*_st_*** decreased with the generation of a bathochromic absorption band and negative CD signal at 350–400 nm, reflecting the photoirradiation process from THI-***E***-**M3*_st_*** to PSS_340_-***Z***-**M3*_mst_*** (Figure S46b, orange line). Furthermore, upon warming the aqueous sample of PSS_340_-***Z***-**M3*_mst_*** at 343 K for 5 h, the absorption band PSS_340_-***Z***-**M3*_mst_*** in the 380–440 nm region decreased, accompanied by an increase in intensity in the 300–365 nm region. After cooling and aging process, the negative Cotton effect reappeared in the CD spectra, indicating the recovery of Z-M3mst and the reformation of supramolecular chirality (Figure S45d). The slight inconsistency in intensity can be attributed to incomplete photoisomerization.


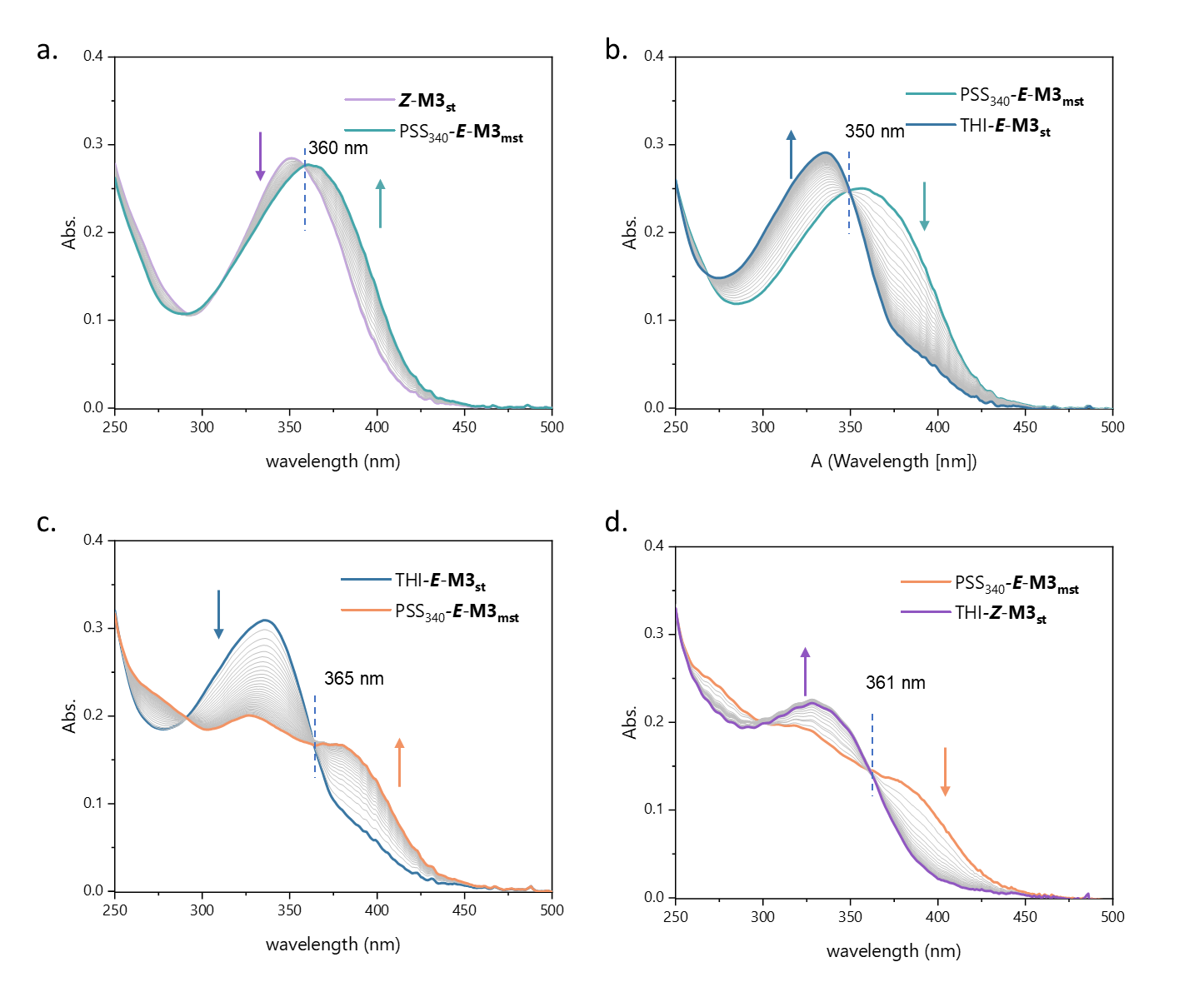


Figure **S45**. UV-vis absorption spectra of ***Z***-**M3_st_** (20 μM, in water) (a) upon 340 nm light irradiation for 5 min at 5 ºC to form PSS_340_-***E***-**M3_mst_**, (b) maintaining the solution in the dark at 5 ºC for 2 h to reach THI-***E***-**M3_st_**, (c) subsequent irradiating with 340 nm light at 5 ºC for 10 min to yield PSS_340_-***Z***-**M3_mst_**, (d) finally keeping in the dark at 70 ºC for 5 h to recover THI-***Z***-**M3_st_**.


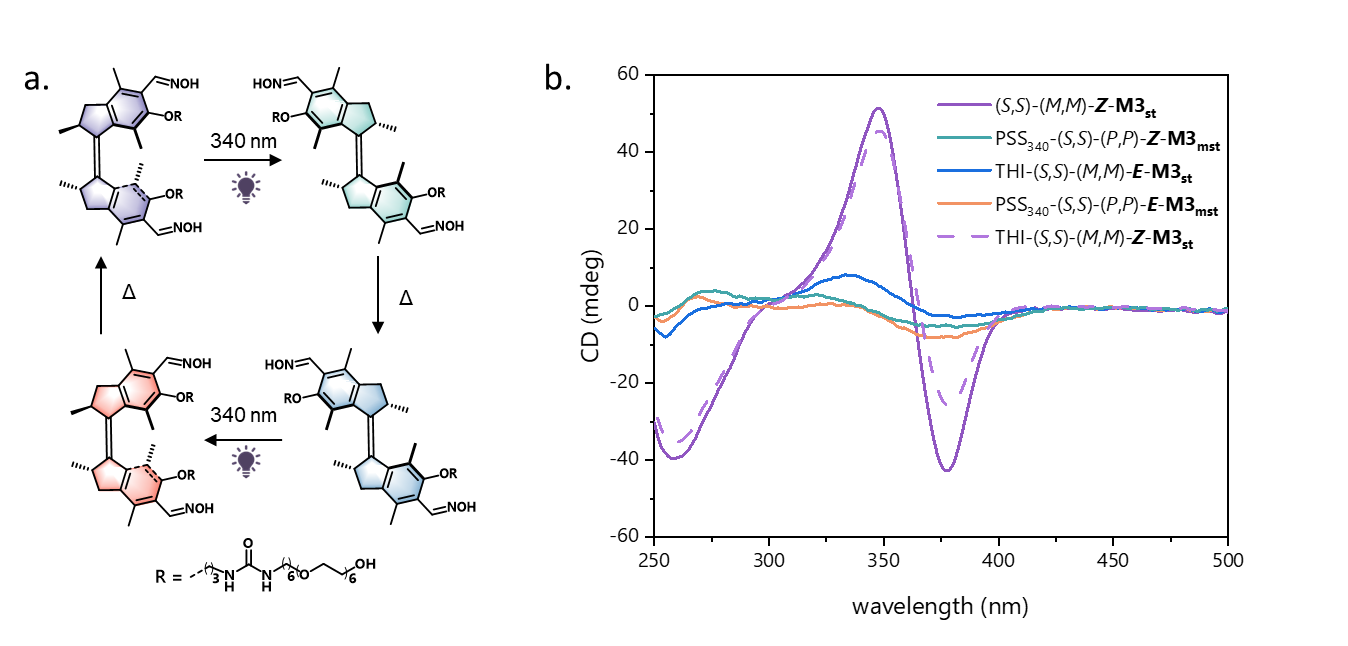


Figure **S46**. (a). Structural changes during the four-step unidirectional isomerization of the hydroxy sulfonate motors. (b). Changes in the CD spectra of ***Z***-**M3_st_** during photochemical reaction and thermal helix inversion process.

1. **Rotary cycle of *Z-*M3_st_ upon irradiation**

Demonstrating the reversibility of oxime formation is essential for establishing the broader concept of reversible aldehyde modification in our system. To address this, we performed acid-catalyzed hydrolysis of the oxime-functionalized motor in both the monomeric state (methanol/water mixture) and the aggregated supramolecular state (pure water), and monitored the process using UV–vis and CD spectroscopy.

**1. Monomeric state hydrolysis (****MeOH/H₂O mixture)**
Before hydrolysis, the motor ***Z***-**M3_st_** displayed a maxim absorption band at 357 nm with a positive CD signal 310-280 nm, showing the monomeric state chirality in MeOH/H₂O mixture. Upon addition of HCl (2M) and stirring overnight at 20 °C, we observed a clear red-shit of the spectra in both UV–vis and CD, consistent with regeneration of the aldehyde motor. The maxima shift back to the characteristic absorption of the aldehyde species confirms that the oxime bond undergoes efficient hydrolysis in monomeric state even at room temperature (Figure S47).


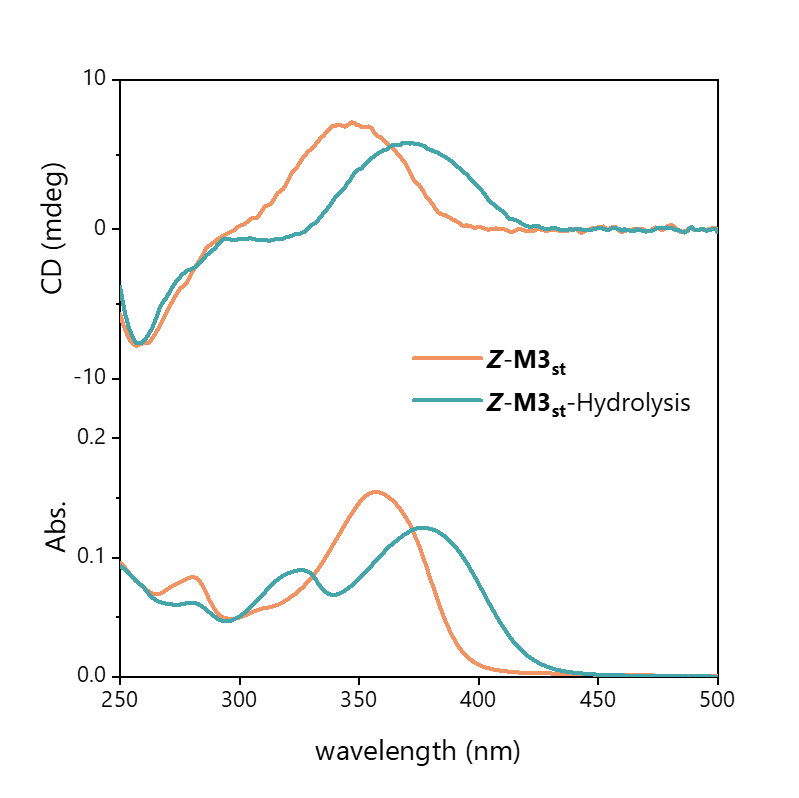


Figure **S47**. Changes in the UV/vis absorption (bottom part) and CD spectra (upper part) of ***Z***-**M3_st_** (10 µM, in a 1/1 solution of 2M HCl and MeOH mixture) before (orange line) and after hydrolysis in MeOH/H₂O mixture (green line).

**2. Aggregated state hydrolysis**
In contrast, when the supramolecular polymers were treated with 2 M HCl in water at room temperature, no significant changes were detected in either UV–vis or CD spectra. This suggests that within the assembled fibers, oxime hydrolysis is strongly disfavored, likely due to restricted accessibility of the reactive site in the crowded supramolecular environment.

**
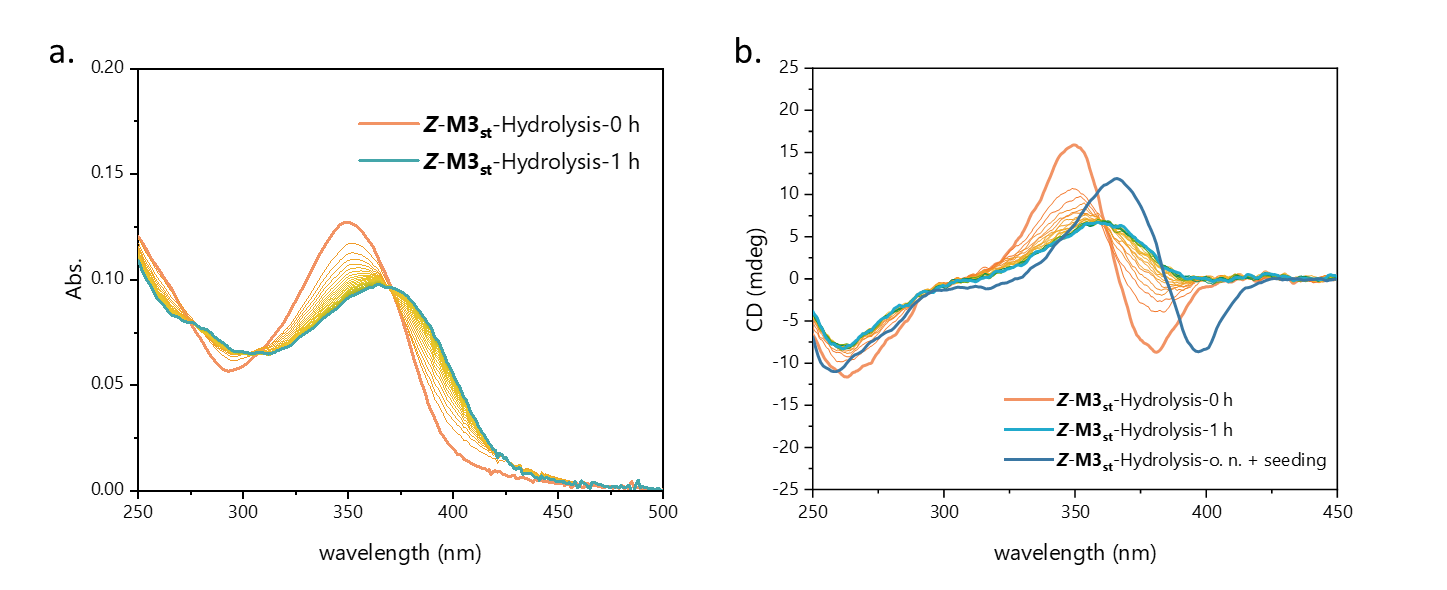
**

Figure **S48**. (a). Changes in the UV/vis absorption spectra of ***Z***-**M3_st_** (10 µM, in a solution of 2M HCl) during the hydrolysis process (from orange to green line) in water at 60 °C. (b). Changes in the CD spectra of ***Z***-**M3_st_** (10 µM, in a solution of 2M HCl) during the hydrolysis process (from orange to green line) in water at 60 °C and recovery of ***Z***-**M3_st_** after aging overnight and seeding.

When the samples were heated to 60 °C, under which the fibers partially disassemble, we observed the red shifts in both UV/vis and CD, which is consistent with oxime hydrolysis and reformation of the aldehyde motor (Figure S48). This indicates that reversibility can be achieved once the supramolecular packing is disrupted. However, after aging the hydrolyzed samples at room temperature overnight, the supramolecular chirality was not observed in the recovered aldehyde motor solution. This outcome may be attributed to the relatively low concentration or the acidic conditions, which could influence the nucleation process of ***Z***-**M1_st._** Subsequent seeding with a solution of ***Z***-**M1_st_** (10 µM in water, 50 µL) restored the characteristic negative cotton effect from the self-assembled aldehyde motor (Figure S48b).

1. **Cryo-TEM analysis of *Z*-M3_st_**


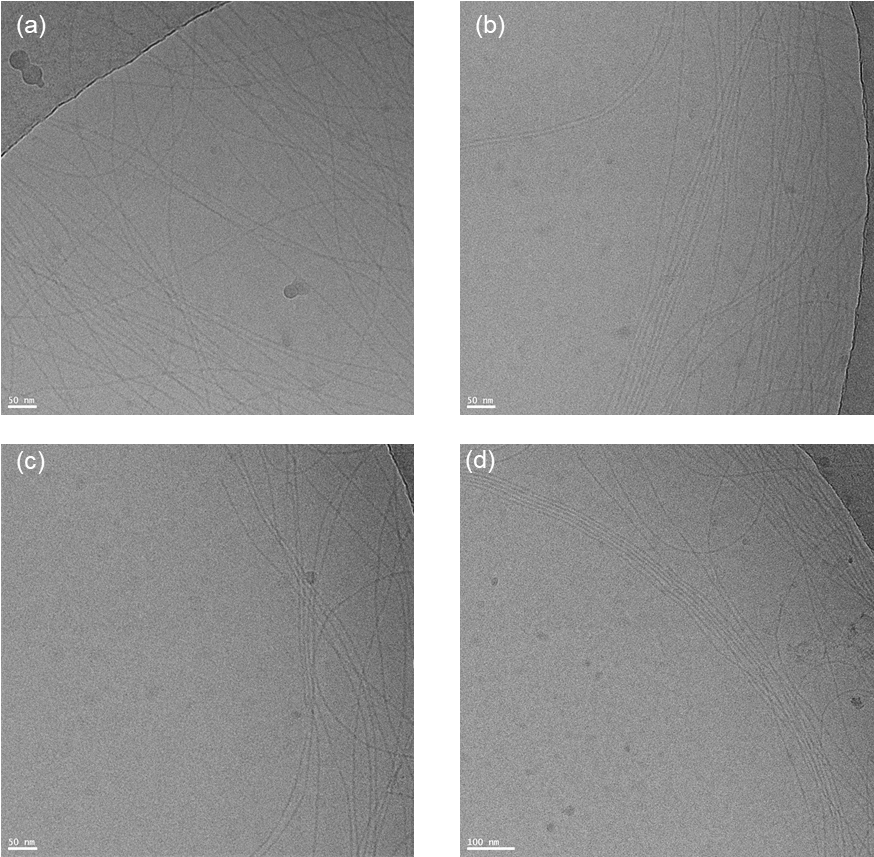


Figure **S49**. Cryo-TEM images of ***Z***-**M3_st_**, obtained after adding NH_2_OH (1 M in water, 1000 eq.) to an aq. solution of ***Z***-**M1_st_** (1 mg/ml) and after aging for 1 d in the dark, showing the chiral fibers.


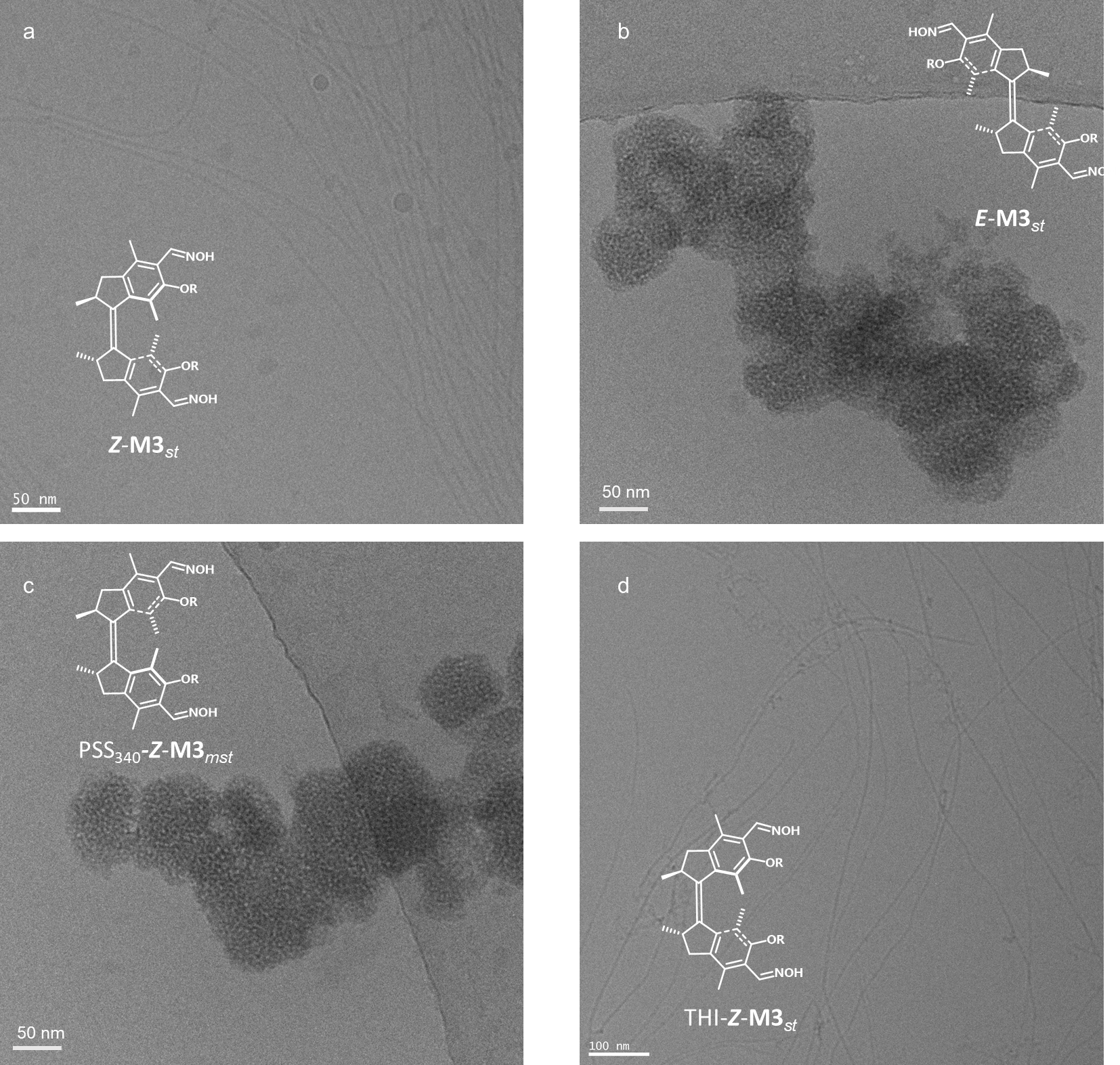


Figure **S50**. Cryo-TEM images of (a). ***Z***-**M3_st_**, obtained by adding NH_2_OH (1 M in water, 1000 eq.) to an aq. solution of ***Z***-**M1_st_** (1 mg/ml) and after aging for 1 d in the dark, showing the chiral fibers. (b). ***E***-**M3_st_**, obtained by irradiation of ***Z***-**M3_st_** at 340 nm for 5min, and subsequently kept in the dark overnight at room temperature. (c). PSS_340_-***Z***-**M3_mst_**, obtained by irradiation of ***E***-**M3_st_** at 340 nm for 10min, and subsequently kept in the dark overnight at room temperature. (d). THI-***Z***-**M3_st_**, obtained by warming of PSS_340_-***Z***-**M3_mst_** at 343 K for 5h, and subsequently cooling at room temperature and kept in the dark overnight.

1. **NMR and HRMS data**


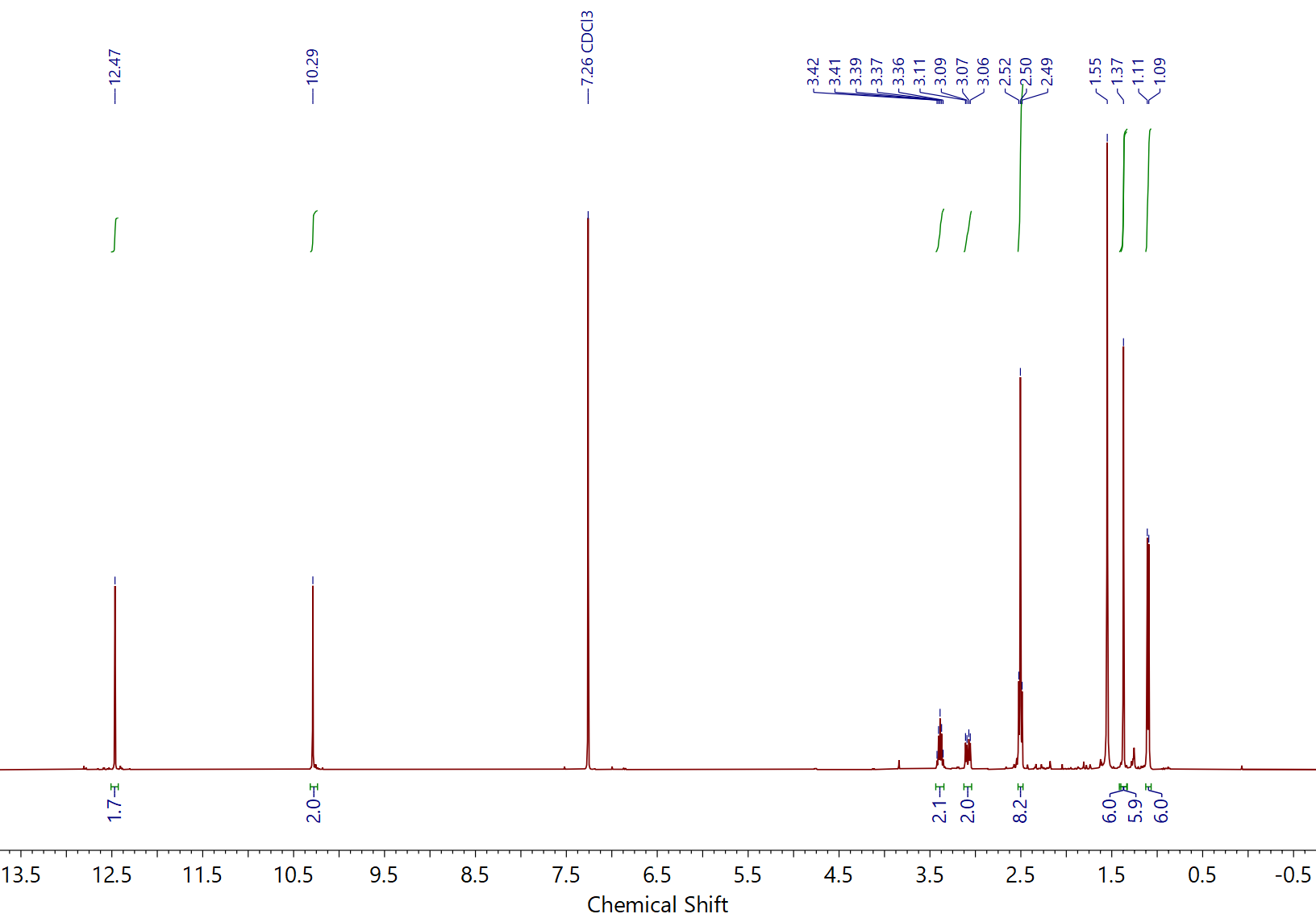


Figure **S51**. ^1^H NMR spectrum of (*S*,*S*)-(*M*,*M*)-***Z***-**2** (400 MHz, CDCl_3_, 298 K).


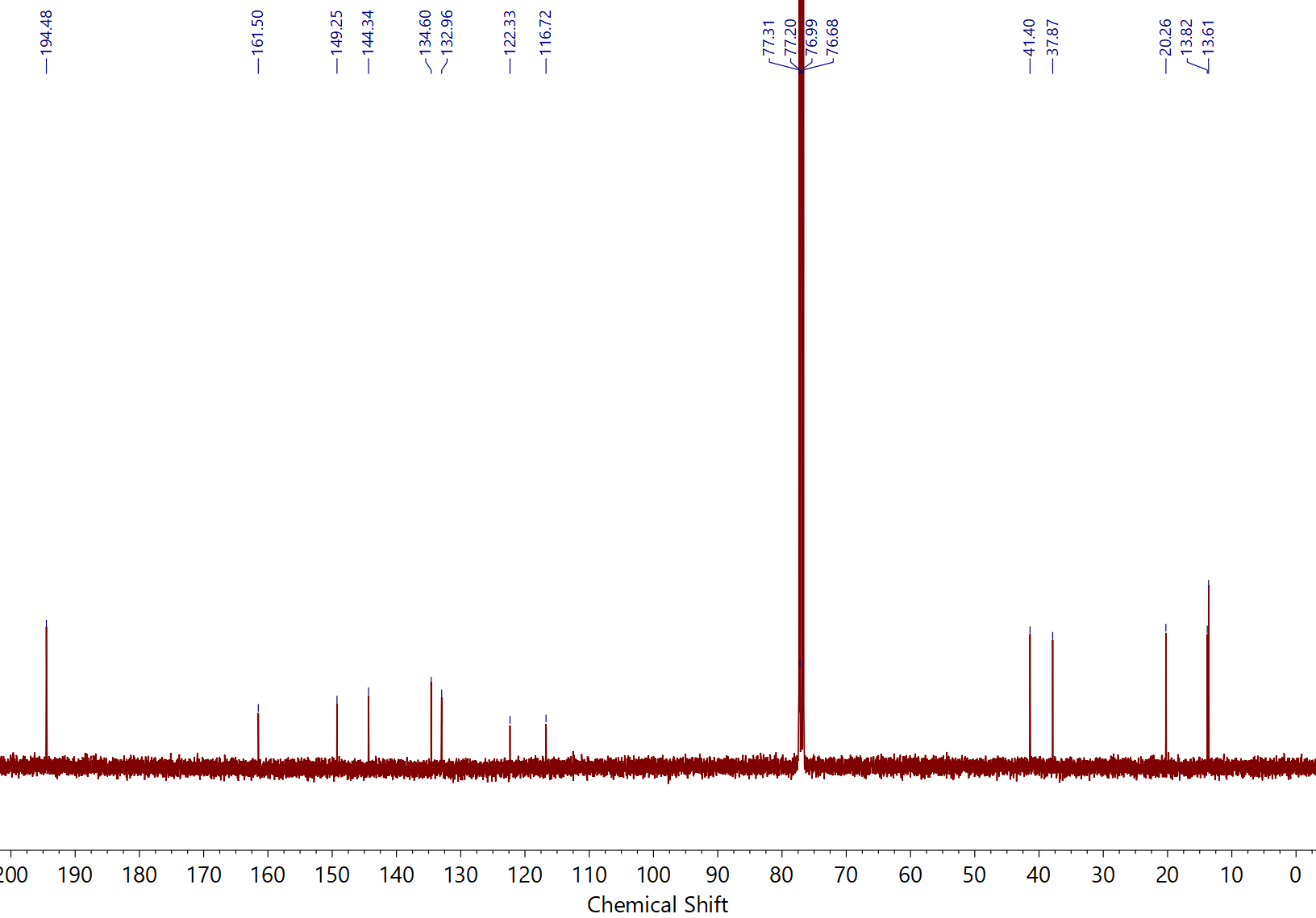


Figure **S52**. ^13^C{^1^H} NMR spectrum of (*S*,*S*)-(*M*,*M*)-***Z***-**2** (101 MHz, CDCl_3_, 298 K).


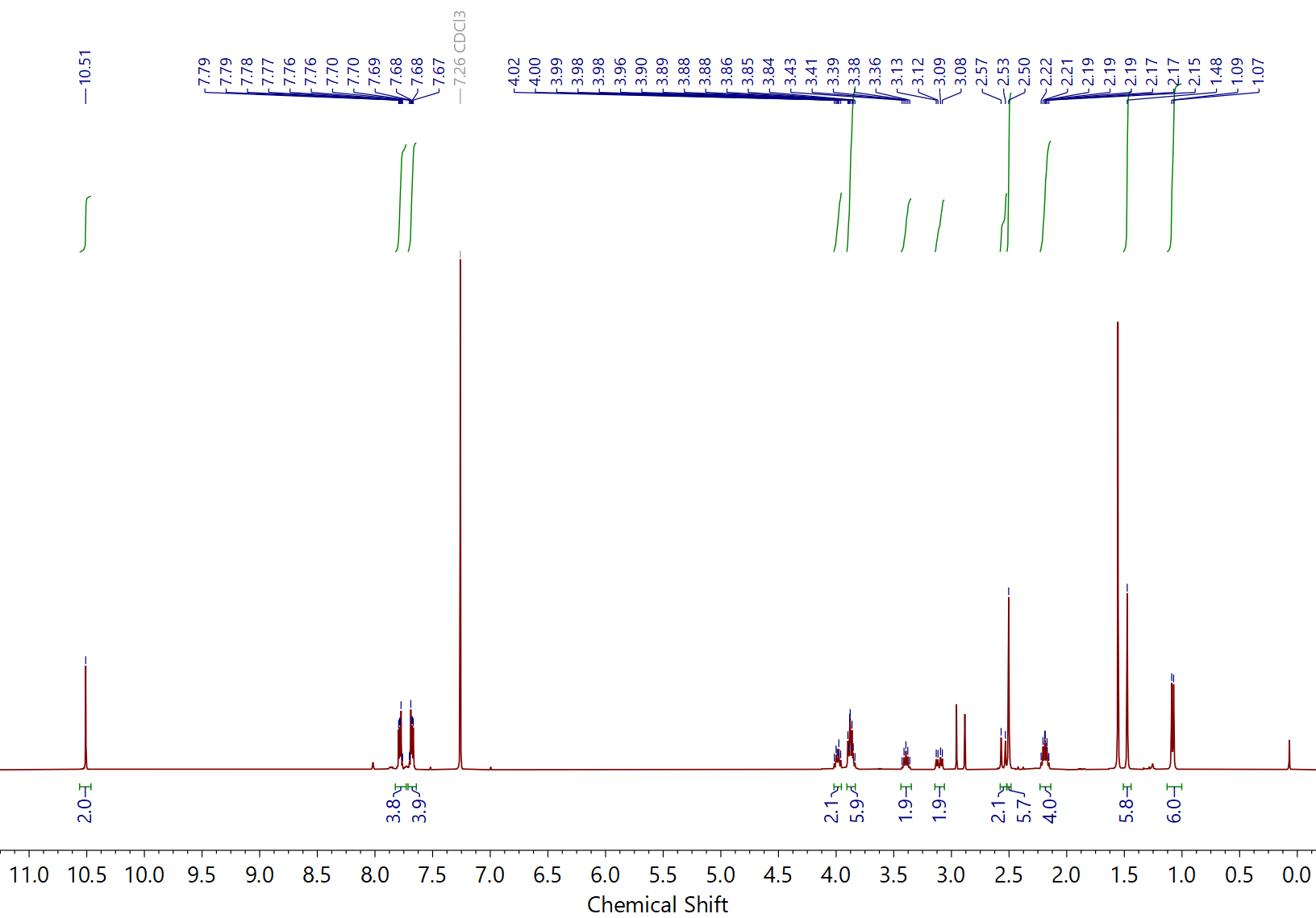


Figure **S53**. ^1^H NMR spectrum of (*S*,*S*)-(*M*,*M*)-***Z***-**3** (400 MHz, CDCl_3_, 298 K).


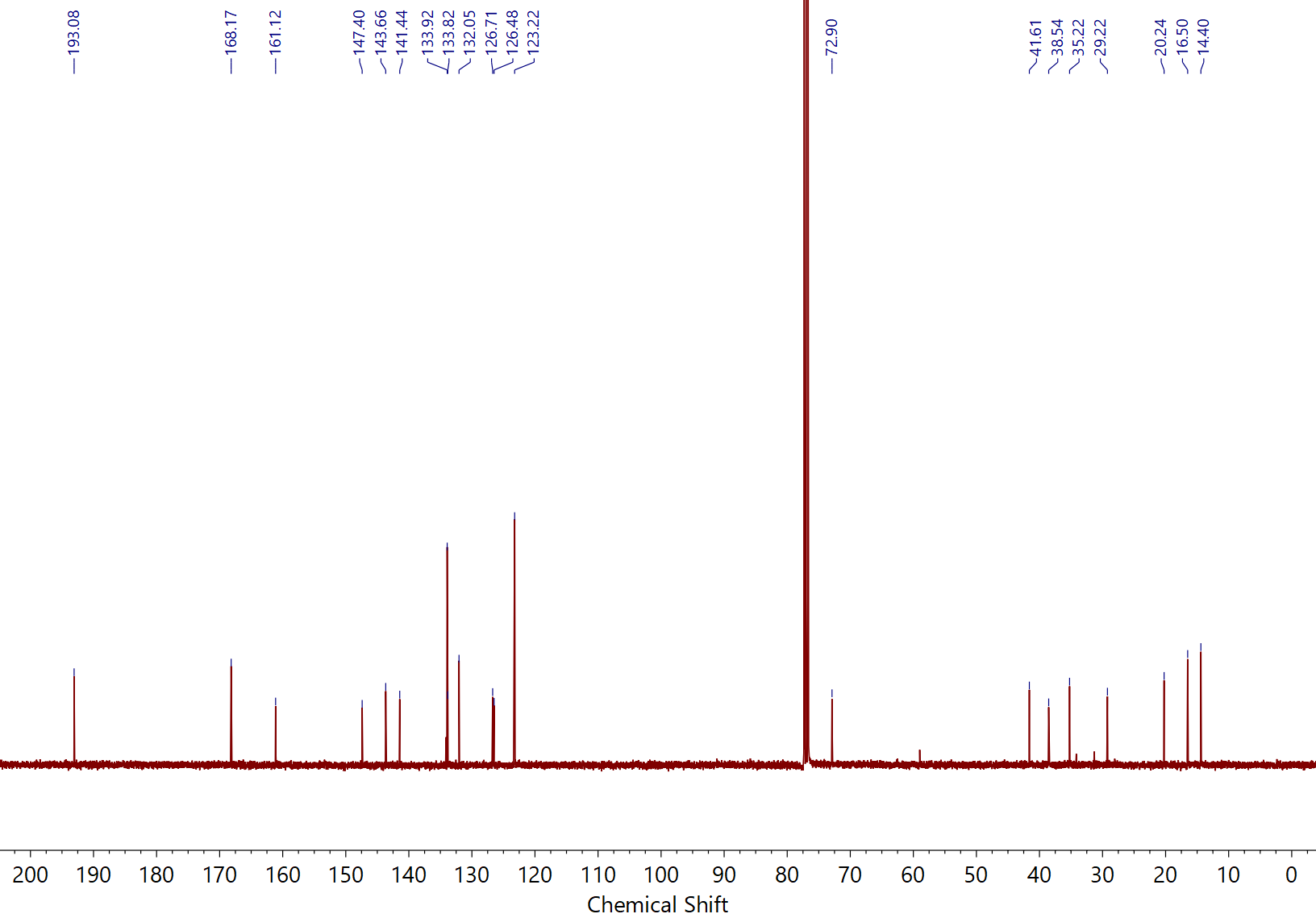


Figure **S54**. ^13^C{^1^H} NMR spectrum of (*S*,*S*)-(*M*,*M*)-***Z***-**3** (101 MHz, CDCl_3_, 298 K).

Figure **S55**. ^1^H NMR spectrum of (*S*,*S*)-(*M*,*M*)-***Z***-**4** (400 MHz, CD_2_Cl_2_, 298 K).

Figure **S56**. ^13^C{^1^H} NMR spectrum of (*S*,*S*)-(*M*,*M*)-***Z***-**4** (101 MHz, CD_2_Cl_2_, 298 K).


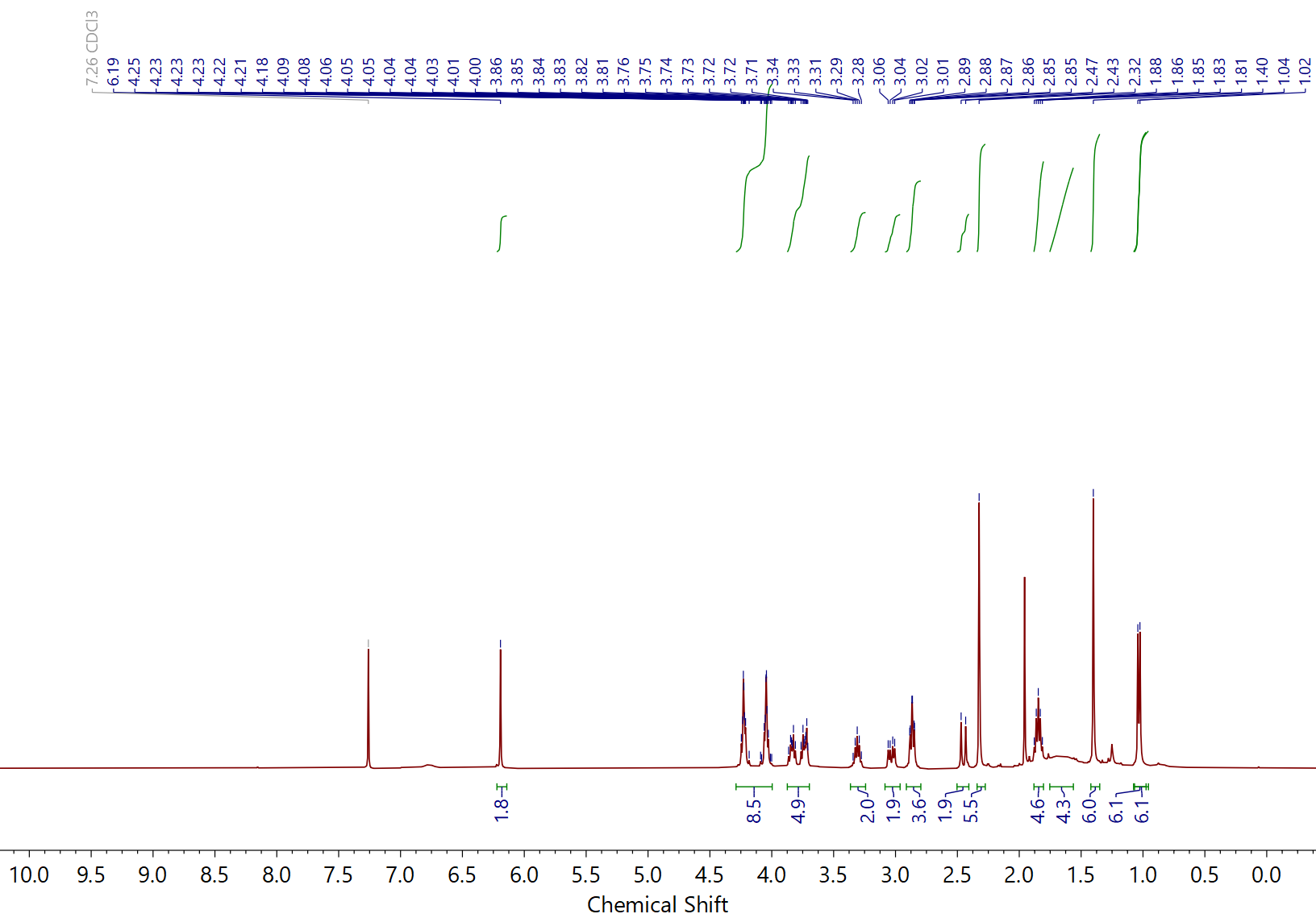


Figure **S57**. ^1^H NMR spectrum of (*S*,*S*)-(*M*,*M*)-***Z***-**5** (400 MHz, CDCl_3_, 298 K).


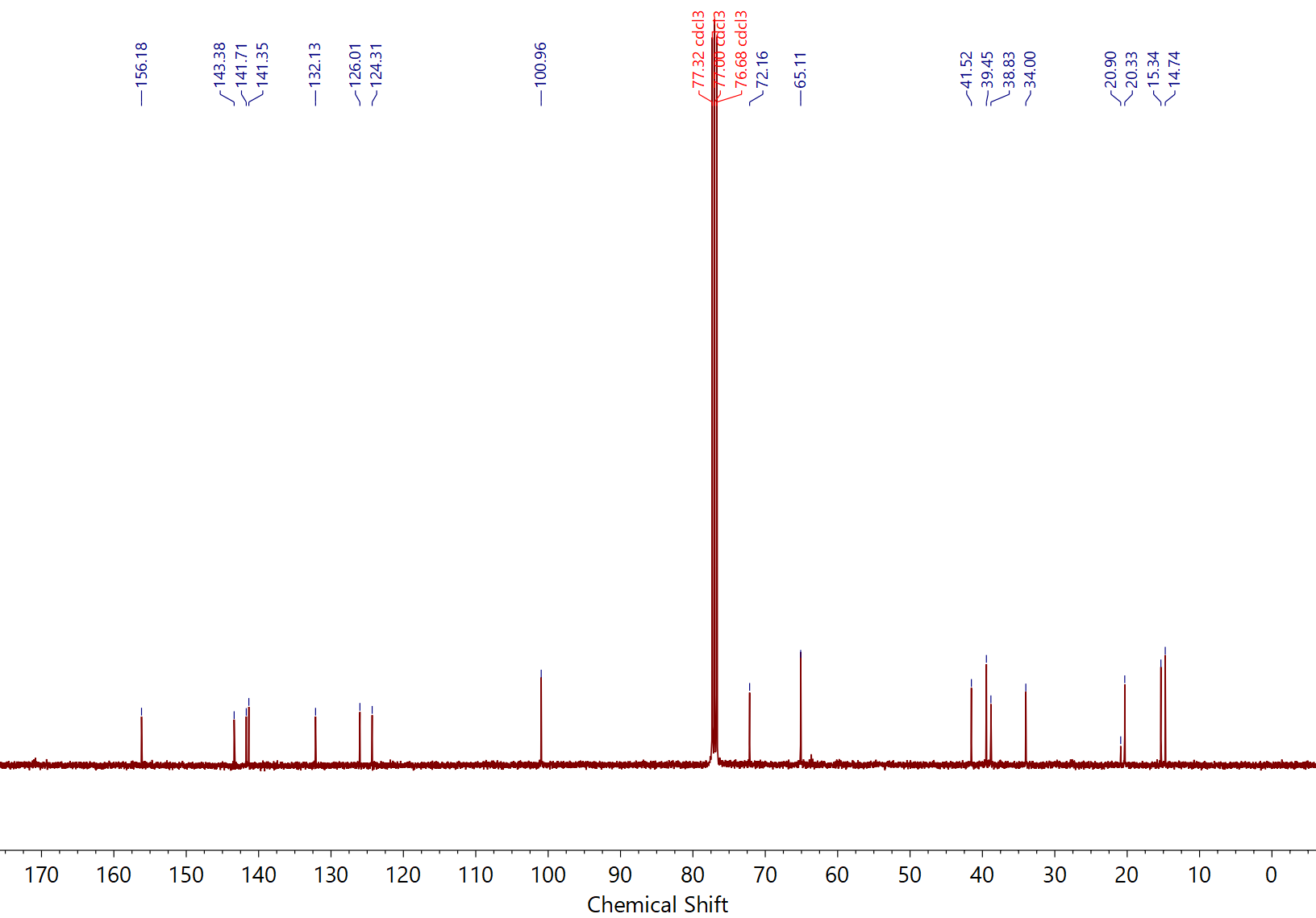


Figure **S58**. ^13^C{^1^H} NMR spectrum of (*S*,*S*)-(*M*,*M*)-***Z***-**5** (400 MHz, CDCl_3_, 298 K).

Figure **S59**. ^1^H NMR spectrum of (*S*,*S*)-(*M*,*M*)-***Z***-**6** (400 MHz, CD_2_Cl_2_, 298 K).

Figure **S60**. ^13^C{^1^H} NMR spectrum of (*S*,*S*)-(*M*,*M*)-***Z***-**6** (400 MHz, CD_2_Cl_2_, 298 K).

Figure **S61**. ^1^H NMR spectrum of (*S*,*S*)-(*M*,*M*)-***Z***-**M1** (400 MHz, CD_2_Cl_2_, 298 K).

Figure **S62**. ^13^C{^1^H} NMR spectrum of (*S*,*S*)-(*M*,*M*)-***Z***-**M1** (400 MHz, CD_2_Cl_2_, 298 K).

Figure **S63**. ^1^H NMR spectrum of **MOMe-NH_2_OH** (400 MHz, CDCl_3_, 298 K).

Figure **S64**. ^13^C{^1^H} NMR spectrum of **MOMe-NH_2_OH** (400 MHz, CDCl_3_, 298 K).


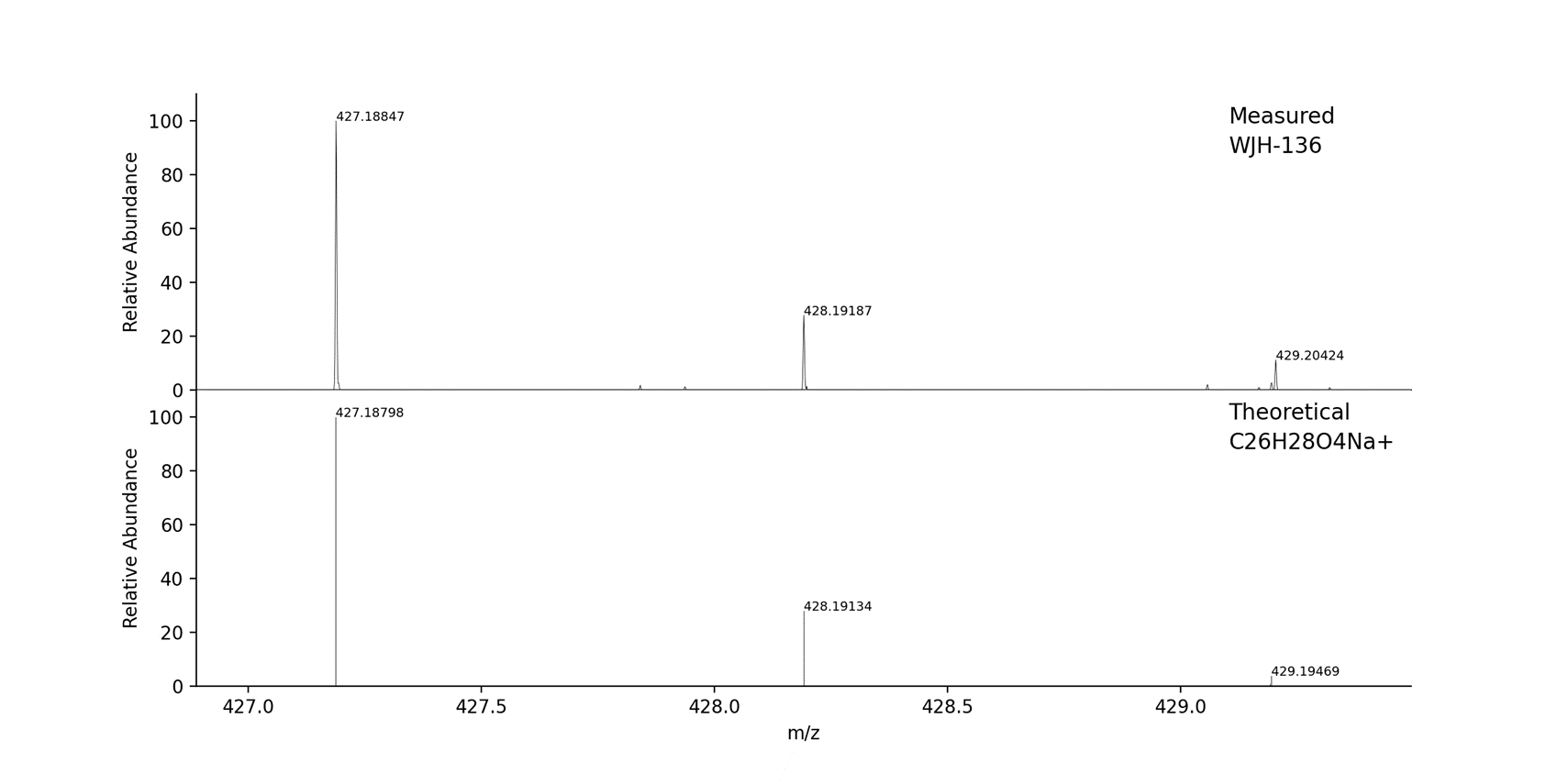


Figure **S65**. HRMS (ESI) spectra of (*S*,*S*)-(*M*,*M*)-***Z***-**2** (top: measured, bottom: theoretical).


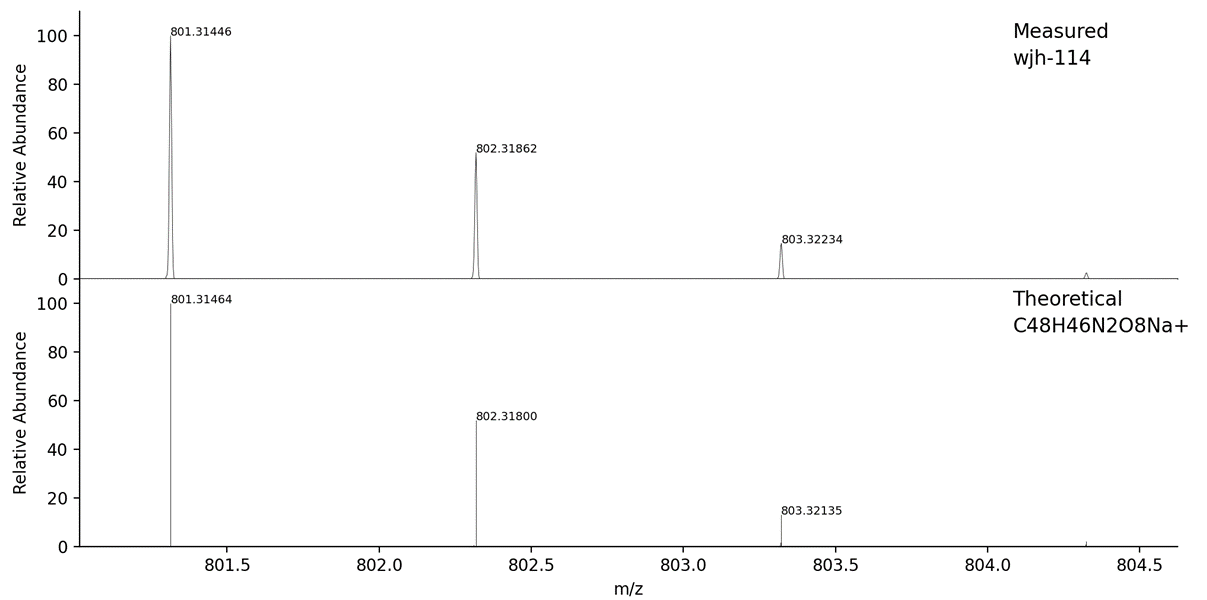


Figure **S66**. HRMS (ESI) spectra of (*S*,*S*)-(*M*,*M*)-***Z***-**3** (top: measured, bottom: theoretical).

**
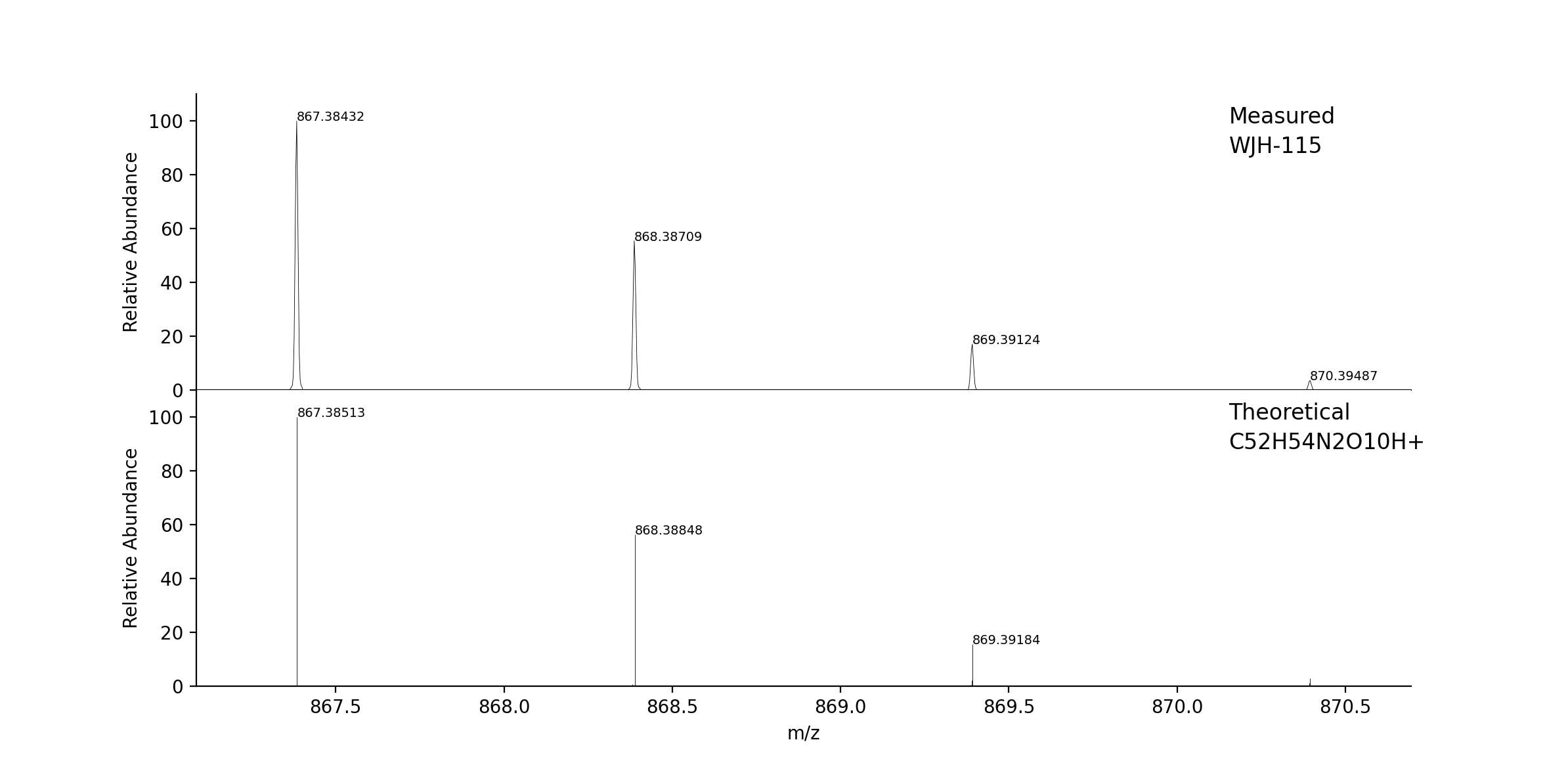
**

Figure **S67**. HRMS (ESI) spectra of (*S*,*S*)-(*M*,*M*)-***Z***-**4** (top: measured, bottom: theoretical).

**
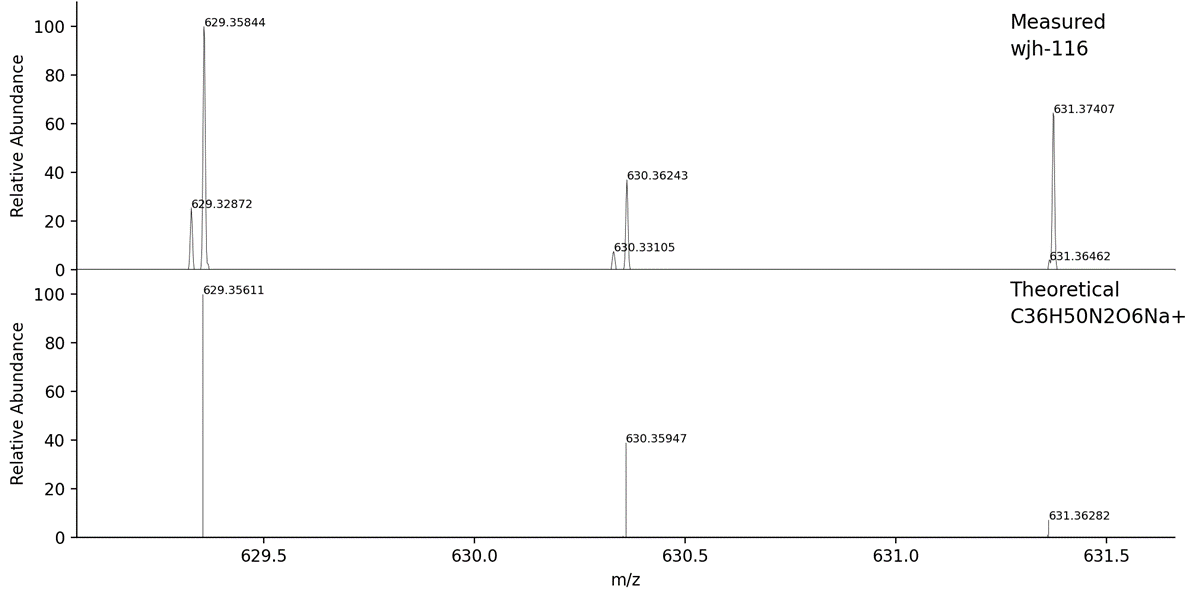
**

Figure **S68**. HRMS (ESI) spectra of (*S*,*S*)-(*M*,*M*)-***Z***-**5** (top: measured, bottom: theoretical).

**
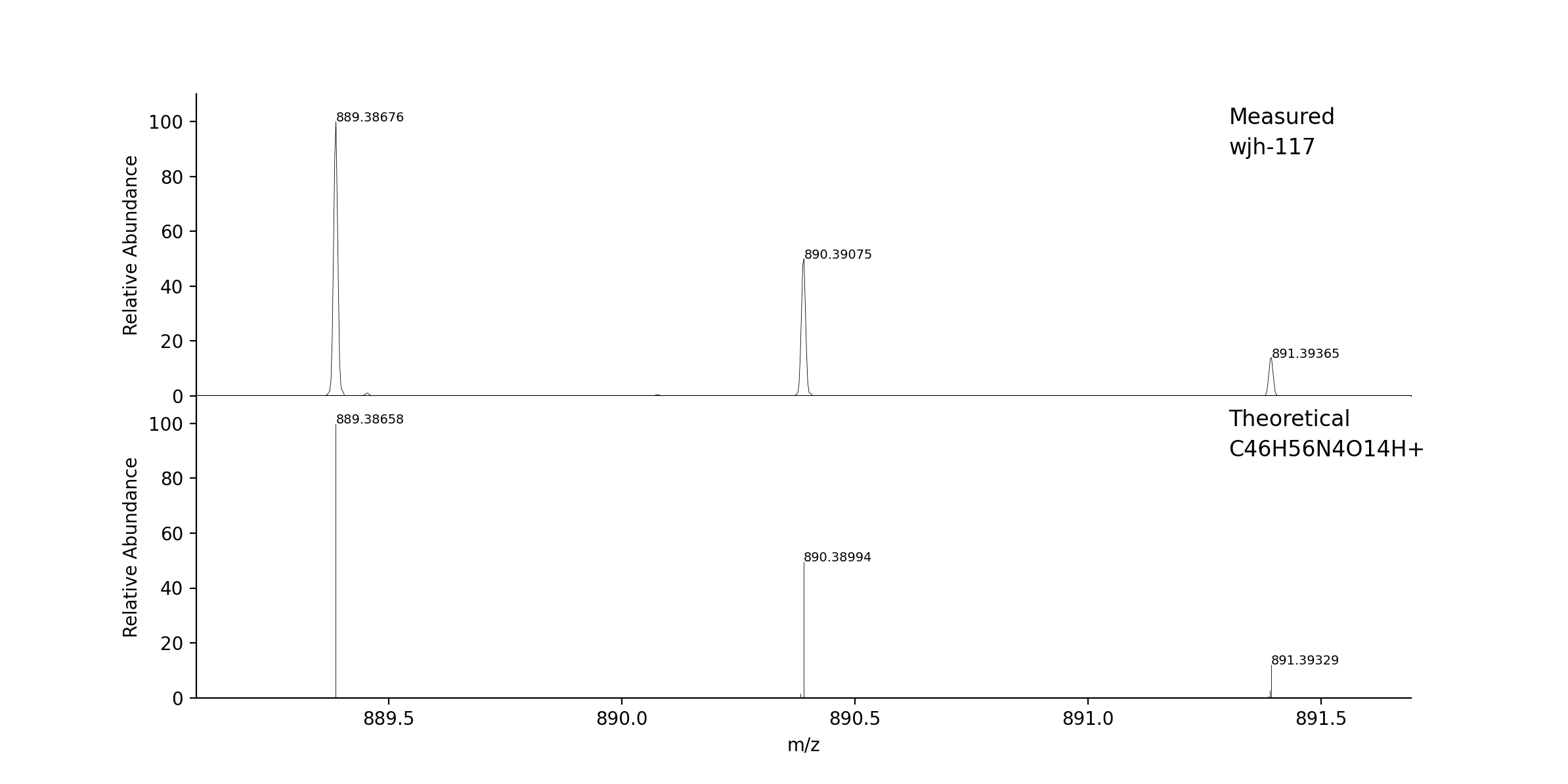
**

Figure **S69**. HRMS (ESI) spectra of (*S*,*S*)-(*M*,*M*)-***Z***-**6** (top: measured, bottom: theoretical).

**
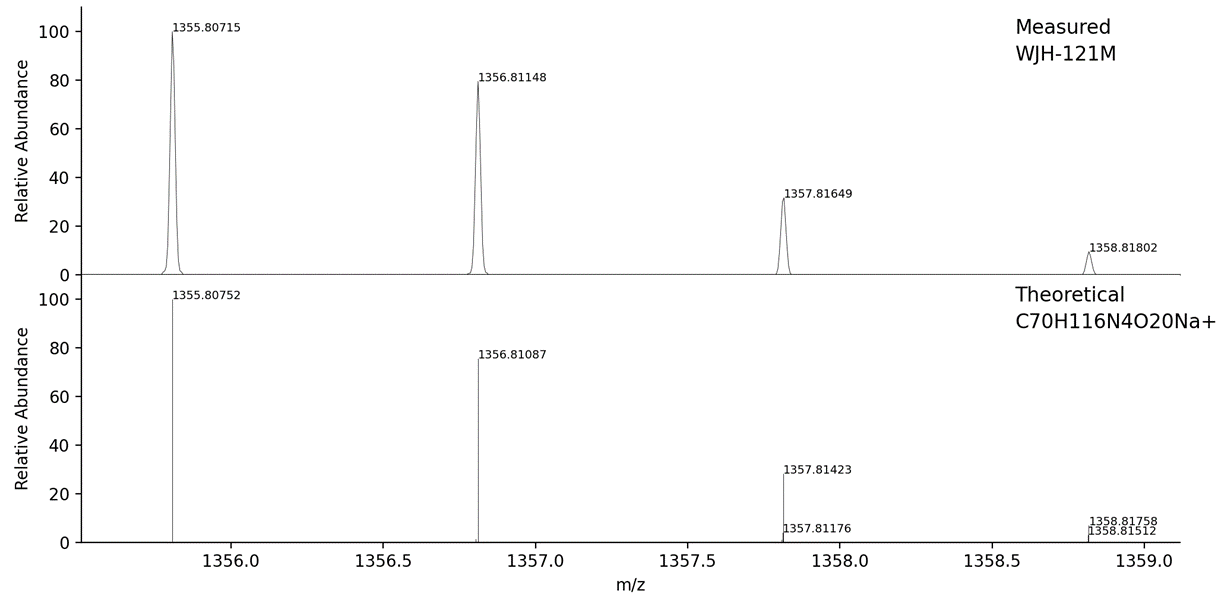
**

Figure **S70**. HRMS (ESI) spectra of (*S*,*S*)-(*M*,*M*)-***Z***-**M1** (top: measured, bottom: theoretical).

1. **Reference**

[1] J. Sheng, W. Danowski, A. S. Sardjan, J. Hou, S. Crespi, A. Ryabchun, M. P. Domínguez, W. Jan Buma, W. R. Browne, B. L. Feringa, *Nat. Chem.* **2024**, *16*, 1330-1338.

[2] F. Xu, S. Crespi, G. Pacella, Y. Fu, M. C. A. Stuart, Q. Zhang, G. Portale, B. L. Feringa, *J. Am. Chem. Soc.* **2022**, *144*, 6019-6027.

[3] J. C. M. Kistemaker, S. F. Pizzolato, T. van Leeuwen, T. C. Pijper, B. L. Feringa, *Chem. Eur. J.* **2016**, *22*, 13478-13487.

[4] T. F. A. De Greef, M. M. J. Smulders, M. Wolffs, A. P. H. J. Schenning, R. P. Sijbesma, E. W. Meijer, *Chem. Rev.* **2009**, *109*, 5687-5754.

[5] M. M. J. Smulders, M. M. L. Nieuwenhuizen, T. F. A. de Greef, P. van der Schoot, A. P. H. J. Schenning, E. W. Meijer, *Chem. Eur. J.* **2010**, *16*, 362-367.

[6] M. M. J. Smulders, A. P. H. J. Schenning, E. W. Meijer, *J. Am. Chem. Soc.* **2008**, *130*, 606-611.

[7] K. Stranius, K. Börjesson, *Scientific Reports* **2017**, *7*, 41145.

[8] H. J. Kuhn, S. E. Braslavsky, R. Schmidt, *Pure Appl. Chem.* **2004**, *76*, 2105-2146.

[9] M. Montalti, A. Credi, L. Prodi, M. T. Gandolfi, *Handbook of photochemistry*, CRC press, **2006**.

[10] S. Hoops, S. Sahle, R. Gauges, C. Lee, J. Pahle, N. Simus, M. Singhal, L. Xu, P. Mendes, U. Kummer, *Bioinformatics* **2006**, *22*, 3067-3074.

[11] N. Singh, B. Lainer, G. J. M. Formon, S. De Piccoli, T. M. Hermans, *J. Am. Chem. Soc.* **2020**, *142*, 4083-4087.

[12] N. Singh, A. Lopez-Acosta, G. J. M. Formon, T. M. Hermans, *J. Am. Chem. Soc.* **2022**, *144*, 410-415.

[13] S. Amano, T. M. Hermans, *J. Am. Chem. Soc.* **2024**, *146*, 23289-23296.

[14] Z. Yang, F. Esteve, C. Antheaume, J.-M. Lehn, *J. Am. Chem. Soc.* **2024**, *146*, 15438-15445.
